# Blood flow patterns in mice are regulated by interpericyte tunneling nanotubes connecting functionally-opposite neuronal areas

**DOI:** 10.1038/s41467-026-71804-2

**Published:** 2026-04-13

**Authors:** Jesse Gardner-Russell, Mahmoud Haddara, Anna Y. M. Wang, Deborah Villafranca-Baughman, Peter van Wijngaarden, Bang Bui, Keith R. Martin, Adriana Di Polo, Luis Alarcon-Martinez

**Affiliations:** 1 Ophthalmology, Department of Surgery, University of Melbourne, Melbourne, VIC Australia; 2 Centre for Eye Research Australia, Royal Victorian Eye and Ear Hospital, Melbourne, VIC Australia; 3 Department of Neuroscience, Université de Montréal, Montreal, QC Canada; 4 Centre de recherche du Centre Hospitalier de l’Université de Montréal (CRCHUM), Montreal, QC Canada; 5 The Florey Institute of Neuroscience and Mental Health, Melbourne, VIC Australia; 6 Department of Optometry & Vision Sciences, The University of Melbourne, Melbourne, VIC Australia

**Keywords:** Retina, Neuro-vascular interactions

## Abstract

Functioning of the central nervous system is critically dependent on precise blood delivery achieved through the formation of blood flow patterns or differences in flux between vessels. Nevertheless, how these blood flow patterns are generated is a fundamental question that is incompletely understood. Here, we show, in live imaging experiments on mice, that specialized pericytes connected via tunneling nanotubes regulate flux patterns by modulating the response of different capillaries in opposing ways, homogenizing blood flow, which is vital to supply functionally-opposite neuronal areas of the retina. We also identify that this capacity for stimulus-induced flow homogenization is lost after the ablation of interpericyte tunneling nanotubes via laser or during ischemia-reperfusion injury, a pathological condition that leads to widespread disruption of interpericyte tunneling nanotubes. These findings contribute to understanding neurovascular coupling and blood delivery in the retina.

## Abstract (web summary)

Although central nervous system’s function depends on blood flow patterns, the mechanism behind their formation is unknown. Here the authors show that specialized pericytes connected by tunneling nanotubes control flux patterns in the mouse retina.

## Introduction

Pioneering work in vascular physiology by Roy and Sherrington^1^ and Krogh^2,3^ established the basis for the study of neurovascular function. Initially, Roy and Sherrington described that local variations of functional activity in the brain could evoke changes in the cerebral vascular supply^1^. Then, Krogh proposed that capillaries were active elements in this process via “*capillary recruitment*”, where closed capillaries without blood flow open to increase oxygen availability when higher metabolic support was required^2,3^. Although modern techniques of functional neurovascular imaging have disproven the *capillary recruitment* hypothesis, we currently know that some capillaries show lower blood flow than others, which changes during neuronal activity. These local differences between capillaries lead to the formation of blood flow patterns^4–7^.

Blood flow patterns are a physiological phenomenon vital for the appropriate function of the central nervous system (CNS)^4–7^. Thus, it has been suggested that adequate blood flow patterns enhance the extraction of oxygen from blood vessels by surrounding neurons via the local redistribution of blood independently of general blood flow increases^5,6^. Consequently, the delivery of oxygen and energy substrates from the bloodstream depends on these flux disparities between capillaries, whereby a homogeneous capillary pattern (i.e., similar blood flow between microvessels) could potentially provide a better oxygen supply into the extracellular space than heterogeneous flux (i.e., different blood flow between microvessels)^5,6^. Consistent with this, the retina^8^ and brain^7,9^ exhibit heterogeneous blood flow and glucose consumption during unstimulated conditions^10,11^, shifting to a homogeneous pattern after neuronal stimulation (e.g., light stimuli or forepaw stimulation)^4,6,7,12–15^. The ability for focal redistribution of capillary blood flow during neuronal activity implies a different stimulus-dependent response between microvessels: blood flow in faster capillaries must decrease, and flow in slower capillaries must increase during flow homogenization^4,6,16–18^. Blood flow patterns are part of neurovascular coupling, a physiological process by which neurons communicate with surrounding blood vessels to acquire the oxygen and nutrients needed to produce adenosine triphosphate (ATP)^19–22^. Although this is a concept widely known, the precise mechanism by which blood flow patterns are generated in the CNS is a fundamental question that remains largely unsolved.

The recently discovered interpericyte tunneling nanotubes (IPTNTs), the first tunneling nanotube (TNT) to be visualized in living animals^23^ and human retina^24^, provide a structural basis for the sophisticated mechanism of neurovascular coupling seen in the retina and brain. IPTNTs are tubular structures that connect pericytes—cells located at the capillary wall that regulate vessel diameter and blood flow^23,25,26^. In contrast to string capillaries, IPTNTs emerge from pericytes, contain organelles^23,27^, their diameter is ~500nm^23,28–30^, and can conduct calcium signaling between pericytes of distal capillaries, which leads to opposing vascular changes (i.e., dilation/constriction) during light stimulation^23^. Accordingly, IPTNTs may serve as key mediators of intercapillary blood flow differences in the CNS. Here, we demonstrate that the retinal blood flow patterns are regulated by IPTNTs that connect pericytes located at capillaries supplying functionally-opposite neuronal areas (i.e., neurons responding to light—ON cells vs. neurons responding to the absence of light—OFF cells). We also report that retinal ischemia-reperfusion, a condition that leads to widespread IPTNT dysfunction and rupture^23^, results in a loss of this regulation. Our findings uncover the mechanism for the formation of blood flow patterns in the retina, which are likely to be of importance in other parts of the CNS.

## Results

### IPTNTs couple pairs of capillaries with different blood flow

**Fig. 1:**
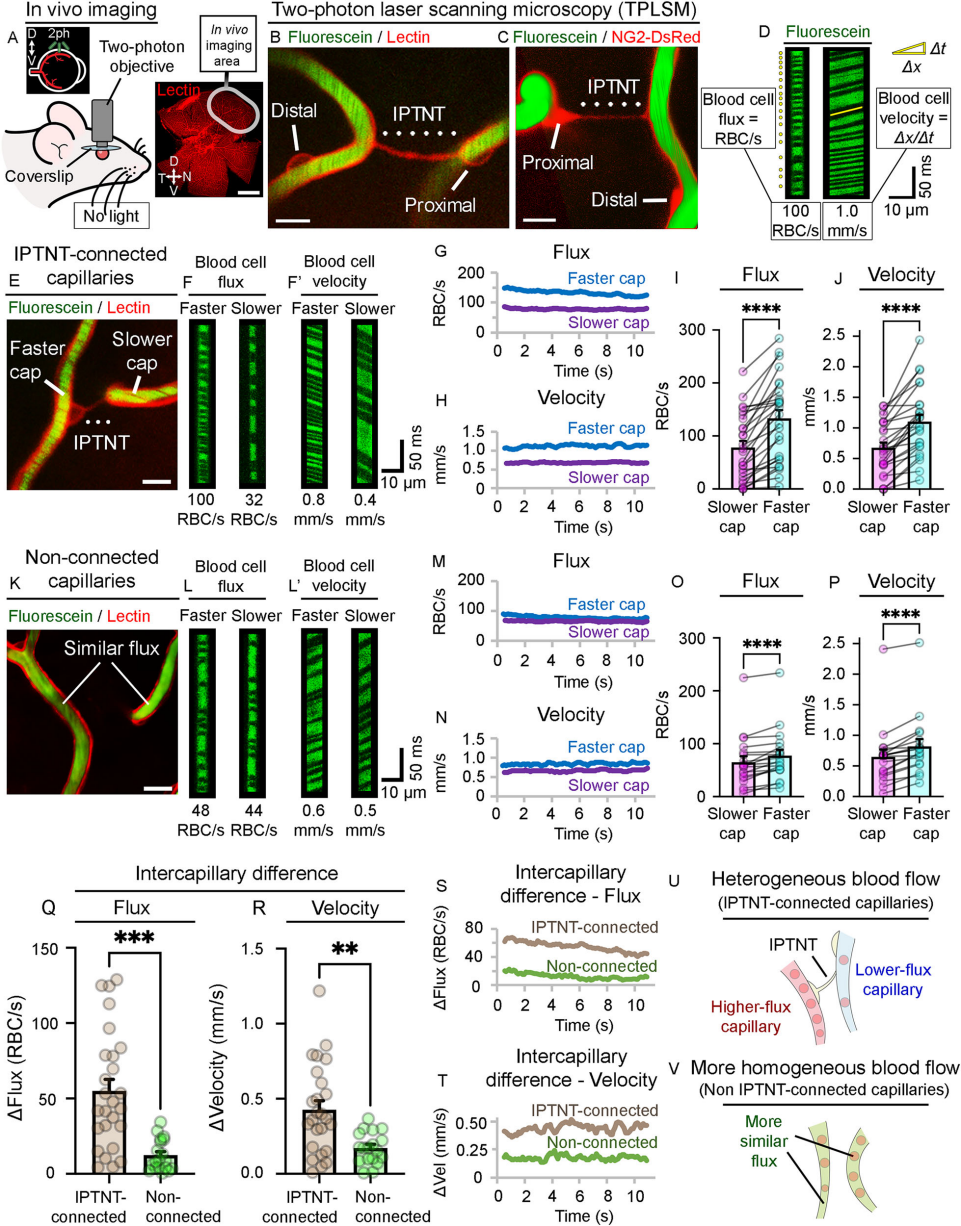
IPTNTs connect capillaries with different blood flow. **A** In vivo setup used to visualize the mouse retina, identified after in flat-mounted retinas (D dorsal, V ventral, T temporal, N nasal, three biological replications). **B**, **C** Capillaries were labeled with intravascular fluorescein (green) and IPTNTs/pericytes with intravitreal TRITC-lectin (red) (**B**) or by using NG2-DsRed mice (**C**, three biological replications). IPTNTs connect pericytes located at adjacent capillaries. **D** Quantification method (see “Methods”) for blood cell flux (left) and velocity (right), where we identify red blood cells (RBCs) as shadows (yellow dots). **E**–**J** IPTNT-connected vessels (**E**) and their line-scan recordings (**F**, **F’**) used to calculate flux (average of 26 capillaries per trace, *N* = 4 mice) (**G**) and velocity traces (average of 25 capillaries per trace, *N* = 3 mice) (**H**). IPTNTs connect pairs of capillaries with different flux (*n* = 52 capillaries, *N* = 4 mice, linear mixed-effect model analysis, ****p < 0.0001) (**I**) and velocity (*n* = 50 capillaries, *N* = 3 mice, linear mixed-effect model analysis, ****p < 0.0001) (**J**). **K**–**P** Non-connected vessels (**K**) and their line-scan recordings (**L**, **L’**) used to calculate flux (average of 19 capillaries per trace, *N* = 5 mice) (**M**) and velocity traces (average of 18 capillaries per trace, *N* = 5 mice) (**N**). Capillaries without IPTNTs showed more similar, but still significantly different blood flow (*n* = 38 capillaries, *N* = 5 mice, linear mixed-effect model analysis, ****p < 0.0001) (**O**) and velocity (*n* = 36 capillaries, *N* = 5 mice, linear mixed-effect model analysis, ****p < 0.0001) (**P**). **Q**–**T** The difference between pairs of capillaries is significantly larger in IPTNT-connected vessels compared to non-connected ones for flux (same capillaries in (**G** and **M**), *n* = 26 pairs of IPTNT-connected capillaries, *N* = 4 mice, *n* = 19 pairs of non-connected capillaries, *N* = 5 mice, linear mixed-effect model analysis, ***p = 0.0001) (**Q**, **S**) and velocity (same capillaries in (**H** and **N**), *n* = 25 pairs of IPTNT-connected capillaries, *N* = 3 mice, *n* = 18 pairs of non-connected capillaries, *N* = 5 mice, linear mixed-effect model analysis, **p = 0.002) (**R**, **T**). **U**, **V** Drawings showing blood flow patterns in IPTNT-connected (**U**) and non-connected (**V**) capillaries. **B**, **C**, **E**, and **K** are the sum of multiple frames. Scale bar in (**A**) = 1 mm, in (**B**, **C**, **E**, and **K**) = 10 μm. All data are presented as mean values +/− SEM. Source data are provided as a Source Data file.

To interrogate the role of pericytes and IPTNTs in the regulation of blood flow patterns, we performed in vivo two-photon laser scanning microscopy (TPLSM) to visualize the retinal IPTNTs and the vasculature of living mice (Fig. 1A). We labeled the retinal microvasculature with an intraperitoneal (IP) injection of fluorescein and the IPTNTs and pericytes with an intravitreal injection of TRITC-lectin (Fig. 1B) or using transgenic mice expressing the DsRed fluorescent protein specifically in pericytes (NG2-DsRed) (Fig. 1C)^23,25^. As vessels located at different sites within the vascular tree show different blood flow and velocity due to basal anatomical disparities^31^, we quantified the blood cell flux or flow (RBC/s) and blood cell velocity (mm/s) (Fig. 1D) in nearby capillaries with pericytes paired via IPTNTs, hereafter referred to as IPTNT-connected capillaries (Fig. 1E)^32^. Basal parameters of blood cell flux (RBC/s) and velocity (mm/s) were positively correlated (Supplementary Fig. 1A–C) and were not impacted by the placement of the coverslip to facilitate TPLSM imaging (Supplementary Fig. 1D). In the dark, one capillary in each IPTNT-coupled pair had faster blood cell flux and velocity (faster capillary) than the other (slower capillary) (Fig. 1F–J and Movie S1). Capillaries that were adjacent but not linked by IPTNTs (Fig. 1K) showed more similar blood cell flux and velocity (Fig. 1L–P) and, therefore, a reduced difference in blood flow/velocity compared to IPTNT-connected vessels (Fig. 1Q–T). This suggests that IPTNTs generate a more heterogeneous blood flow in connected vessels than in capillaries without IPTNTs (Fig. 1U, V).

### IPTNTs connect pericytes in vessels supplying functionally-opposite neuronal areas

**Fig. 2:**
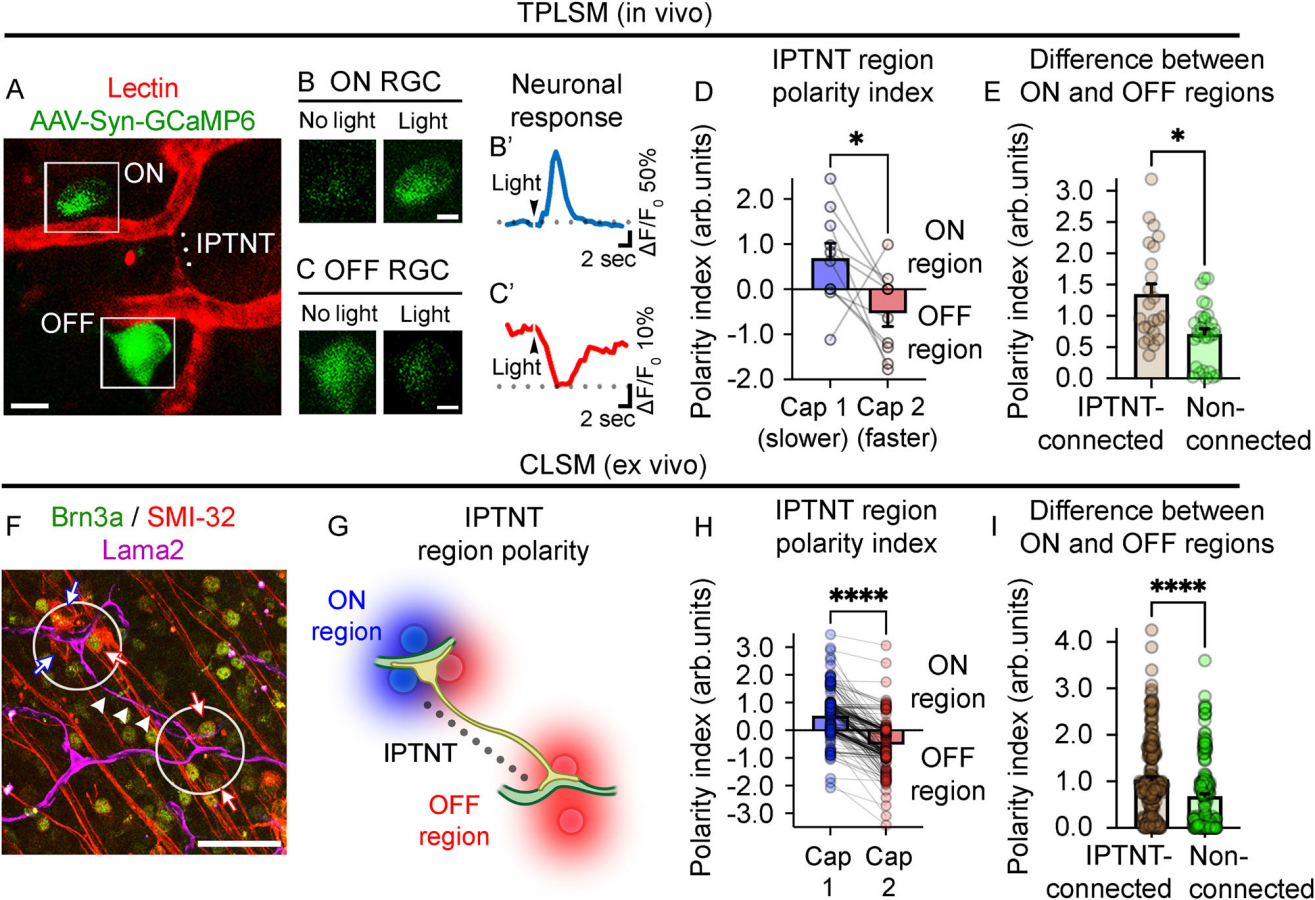
Blood flow patterns in IPTNT-connected capillaries support functionally-opposite neuronal areas. **A**–**C** In vivo IPTNT labeled with intravitreal TRITC-lectin (red, dotted line) surrounded by RGCs expressing the calcium indicator GCaMP6f (green) (**A**). An ON (**B**) and an OFF RGC (**C**) identified after assessing their light-evoked calcium response (**B’**, **C’**). **D**, **E** Simultaneous in vivo recordings of IPTNTs, blood flow, and RGC responses show that neuronal regions around IPTNT-connected capillaries present opposite responses to light identified by their polarity index, which indicates how ON (positive) or OFF (negative) the area around each capillary is. ON and OFF areas are surrounded by slower and faster IPTNT-connected capillaries, respectively (*n* = 20 areas/capillaries, *n* = 51 cells, *N* = 5 mice, linear mixed-effect model analysis, *p = 0.025) (**D**). The difference in polarity index between areas is reduced around capillaries without IPTNTs compared to areas surrounding IPTNT-connected vessels (*n* = 46 areas, *n* = 107 cells, and *N* = 8 mice in IPTNT-connected group; *n* = 60 areas, *n* = 131 cells, and *N* = 3 mice in non-connected group, linear mixed-effect model analysis, *p = 0.020) (**E**). **F**, **G** Ex vivo retina visualized with confocal laser scanning microscopy (CLSM) and labeled with antibodies against Brn3a (green), SMI-32 (red), and Lama2 (purple), used to identify IPTNTs (arrowheads), α-ON (blue arrows) and α-OFF RGCs (red arrows) as previously^35,38^ around IPTNT-connected capillaries (circles in (**F**)). Drawing of (**F**), depicting an IPTNT (dotted line) connecting vessels and the type of RGCs (red, OFF RGCs; blue, ON RGCs) around capillaries (i.e., circles in (**F**)) (**G**). **H**, **I** Ex vivo polarity-index graph shows IPTNTs connecting opposite-functional neuronal areas along the whole retina calculated after identifying ON and OFF RGCs as previously (α-ON = Brn3a−/SMI-32+; α-OFF = Brn3a+/SMI-32+; F-ON = Foxp1+/Foxp2+; F-OFF = Foxp1−/Foxp2+)^35,38,40^ (*n* = 452 areas, *n* = 759 cells, *N* = 7 mice/group, linear mixed-effect model analysis, ****p < 0.0001) (**H**), which was reduced around capillaries without IPTNTs compared to areas surrounding IPTNT-connected vessels (*n *= 452 areas, *n* = 759 cells/group, *N* = 7 mice in IPTNT-connected group; *n* = 440 areas, *n* = 531 cells/group, *N* = 6 mice in non-connected group, linear mixed-effect model analysis, ****p < 0.0001) (**I**). Scale bars in (**A**) = 10 μm; in (**B**, **C**) = 5 μm, in (**F**) = 50 μm. All data are presented as mean values +/− SEM. Source data are provided as a Source Data file. Arb.units arbitrary units.

As IPTNTs connect faster and slower flowing capillaries, we considered whether this flux pattern might support a particular neuronal organization. In the retina, there are cells that are affected by different visual stimuli. Particularly, ON and OFF retinal ganglion cells (RGCs) are neurons activated by light and the absence of light, respectively. For this purpose, we analyzed the light-evoked calcium response of the RGCs surrounding each IPTNT-connected capillary in living mice expressing the genetically encoded calcium indicator GCaMP6f, and at the same time that we monitored the blood flow patterns. Selective expression of the calcium indicator in RGCs was achieved via intravitreal injection of an AAV-syn-GCaMP6f (Fig. 2A and Movie S2)^25^. Previous studies in the retina^33^ and brain^34^ have shown that a higher number of neuronal spikes is associated with a higher calcium-dependent signal. Accordingly, when light is presented, we found that ON RGCs showed an increase of calcium (examples, Fig. 2B, B’ and Supplementary Fig. 2A). On the other hand, OFF RGCs showed a high calcium signal during the absence of light (examples, Fig. 2C, C’ and Supplementary Fig. 2B). Importantly, the decrease of spikes evoked by light is represented as a decrease in the calcium signal (red traces in Fig. 2C’ and Supplementary Fig. 2B). Consequently, we simply classified each neuron as an ON RGC (i.e., responding to light) or an OFF RGC (i.e., responding to the absence of light) as previously described^35^ (Fig. 2B, C). We then calculated a region polarity index for each vessel, which indicates the contribution of ON and OFF RGCs to the area (~ 0.002 mm^2^) around where the capillary was located. For this, we measured the distance from each RGC soma to each capillary and calculated the RGC’s contribution based on the diffusion coefficient through the extracellular space of glutamate. ON-RGC-related values were represented by positive values, and OFF-RGC-related values by negative ones. The contribution value for all RGCs within an area was summed to obtain a polarity index for each region. The difference between the values of both IPTNT-connected areas provided us with an ON/OFF polarity index associated with a pair of capillaries (see “Methods”). Thus, by simultaneous monitoring of light-evoked neuronal responses and blood flow, we found that each capillary of an IPTNT-connected pair supplied regions of opposite polarity indexes or functionally-opposite (ON versus OFF) areas, with faster capillaries supplying areas with a predominance of OFF cells and slower capillaries feeding areas with a predominance of ON cells (Fig. 2D). Moreover, the difference in polarity index between areas was lower in regions surrounding non-IPTNT connected capillaries compared to areas around IPTNT-connected capillaries (Fig. 2E). To confirm our in vivo results, we identified functionally-opposite RGCs in ex vivo retinas through the detection of proteins expressed specifically by certain subtypes of RGCs via immunohistochemistry. Thus, while some antibodies label the overall RGC population^36^, combinations of several antibodies allow us to identify RGC subtypes^37–39^. Accordingly, the consistency of the neuronal organization around IPTNT-connected capillaries across the retina was confirmed by ex vivo analysis (Fig. 2F–I) with cell-specific markers for ON and OFF RGCs (Fig. 2F and Supplementary Fig. 3A)^35,38,40^. We confirmed that the reported difference in the polarity index between ON and OFF areas was not influenced by the same RGCs in both regions since only 2% of the RGCs analyzed contribute equally to both areas (i.e., cells located at the same distance from both area centers, see “Methods”). To assess whether this region-specific neuronal organization around IPTNTs varied for different neuronal subtypes, we analyzed separately ON/OFF F-RGCs (Supplementary Fig. 3A–C)^40^ and ON/OFF α-RGCs (Supplementary Fig. 3D–E)^35,38^, finding a larger polarity-index difference between IPTNT-connected areas for α-RGCs compared to F-RGCs (Supplementary Fig. 3F). To our knowledge, ON and OFF areas have not been previously reported in the retina. Nevertheless, it has been shown that the mosaic of ON-RGCs is regularly spaced from the mosaic of OFF-RGCs throughout the retina^41^. Thus, it is plausible that IPTNTs connect ON and OFF RGC mosaics at certain points where the polarity index between mosaics becomes larger. Accordingly, we found that 81% (35 out of 43) of pairs of RGC areas with a large ON/OFF difference (i.e., ≥ the mean of the ON/OFF difference between non-IPTNT connected areas + SD) were bridged by IPTNTs, suggesting that large opposite neuronal areas are equipped with IPTNTs to regulate differential blood flow. To confirm whether this functionally-opposite neuronal organization around the IPTNTs may imply a global principle, we analyzed another functional RGC type: the ON-OFF direction-selective RGCs (ooDSGCs) (Supplementary Fig. 3G–I)^37,39^. Due to the limited number of immunohistochemistry protocols for the study of functionally-opposite RGCs, we used RNAscope-specific probes for the detection of mRNA expression in flat-mounted retinas^42^ and to identify ooDSGCs responding to opposite directions (i.e., dorsal direction: RBPMS^+^/Col25a1^+^/Calb1^−^; ventral direction: RBPMS^+^/Col25a1^+^/Calb1^+^)^43^. Accordingly, we found a smaller polarity-index difference between IPTNT-connected areas for ventral/dorsal ooDSGCs compared to ON/OFF RGCs (Supplementary Fig. 3J). Overall, these results support the idea that IPTNTs preferentially regulate capillaries that feed neuronal areas with larger functional opponency.

### Light evokes blood flow homogeneity in IPTNT-connected capillaries

**Fig. 3:**
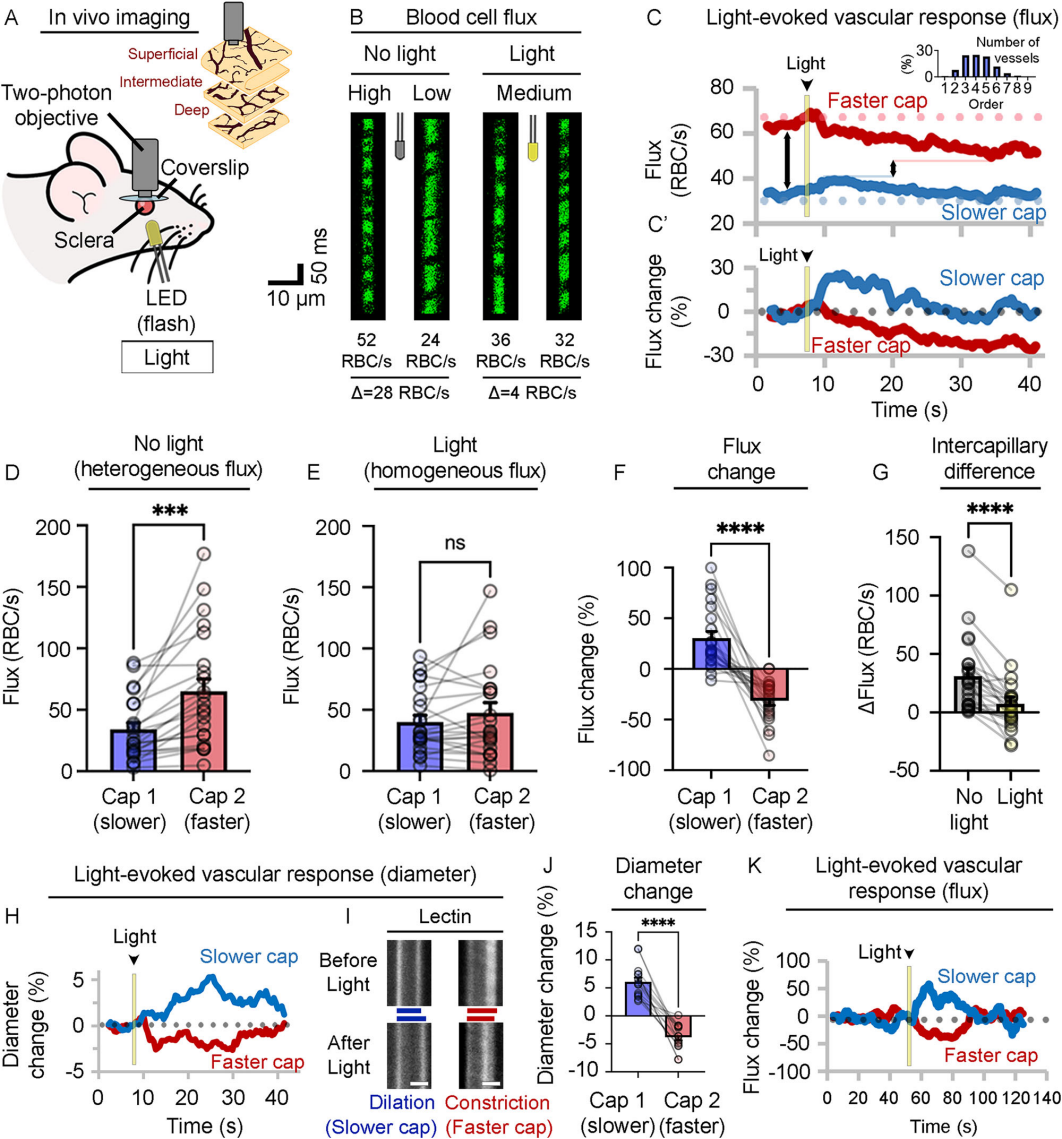
Light stimulation modifies blood flow patterns in IPTNT-connected capillaries. **A** In vivo setup for visualization of the retinal superficial plexus and light stimulation. **B**, **C** Examples of line-scan recordings before (no light) and after light stimulation (light) in IPTNT-connected vessels (**B**) used to calculate blood cell flux (**C**) and flux change traces (**C’**) (average of 22 capillaries per trace, *N* = 7 mice; large double-headed arrow indicates the intercapillary difference before light; small double-headed arrow shows the intercapillary difference after light calculated from the point of maximum increase in the slower capillary to the point of largest decrease in the faster capillary; arrowhead indicates light stimulus). The histogram in (**C**) shows the order of the capillaries where the IPTNTs were located in naive animals. **D**, **G** Maximum response graphs show that, prior to light, IPTNT-connected vessels have different flux or heterogeneity (*n* = 44 capillaries, *N* = 7 mice, linear mixed-effect model analysis, ***p = 0.0002) (**D**). After light, flux difference disappears, leading to flux homogenization (*n* = 44 capillaries, *N* = 7 mice, linear mixed-effect model analysis, ns not significant) (**E**) as slower and faster capillaries increase and decrease their flux, respectively (*n* = 44 capillaries, *N* = 7 mice, linear mixed-effect model analysis, ****p < 0.0001) (**F**). Accordingly, intercapillary flux difference is significantly reduced after stimulation compared to values before light (*n* = 44 capillaries, *N* = 7 mice, two-tailed Wilcoxon matched-pairs signed-rank test, ****p < 0.0001) (**G**). **H**–**J** Light selectively induced dilation and constriction in slower (blue trace) and faster (red trace) IPTNT-connected capillaries, respectively (**H**), identified in this figure (**C**–**G**) (average of 15 capillaries per trace, *N* = 7 mice; arrowhead indicates light). Example of two IPTNT-connected capillaries showing opposite light-evoked responses (**I**, blue and red lines represent the diameter of the capillaries before and after light; see also Movie S4 for another example). After light, slower and faster capillaries increase and decrease their diameter, respectively (*n* = 30 capillaries, *N* = 7 mice, linear mixed-effect model analysis, ****p < 0.0001) (**J**). **K** Longer recordings of IPTNT-connected capillaries (blue trace, slower capillary; red trace, faster capillary; average of 3 capillaries per trace, *N* = 3 mice; arrowhead indicates light stimulus). Scale bars in (**I**) = 5 μm. All data are presented as mean values +/− SEM. Source data are provided as a Source Data file.

**Fig. 4:**
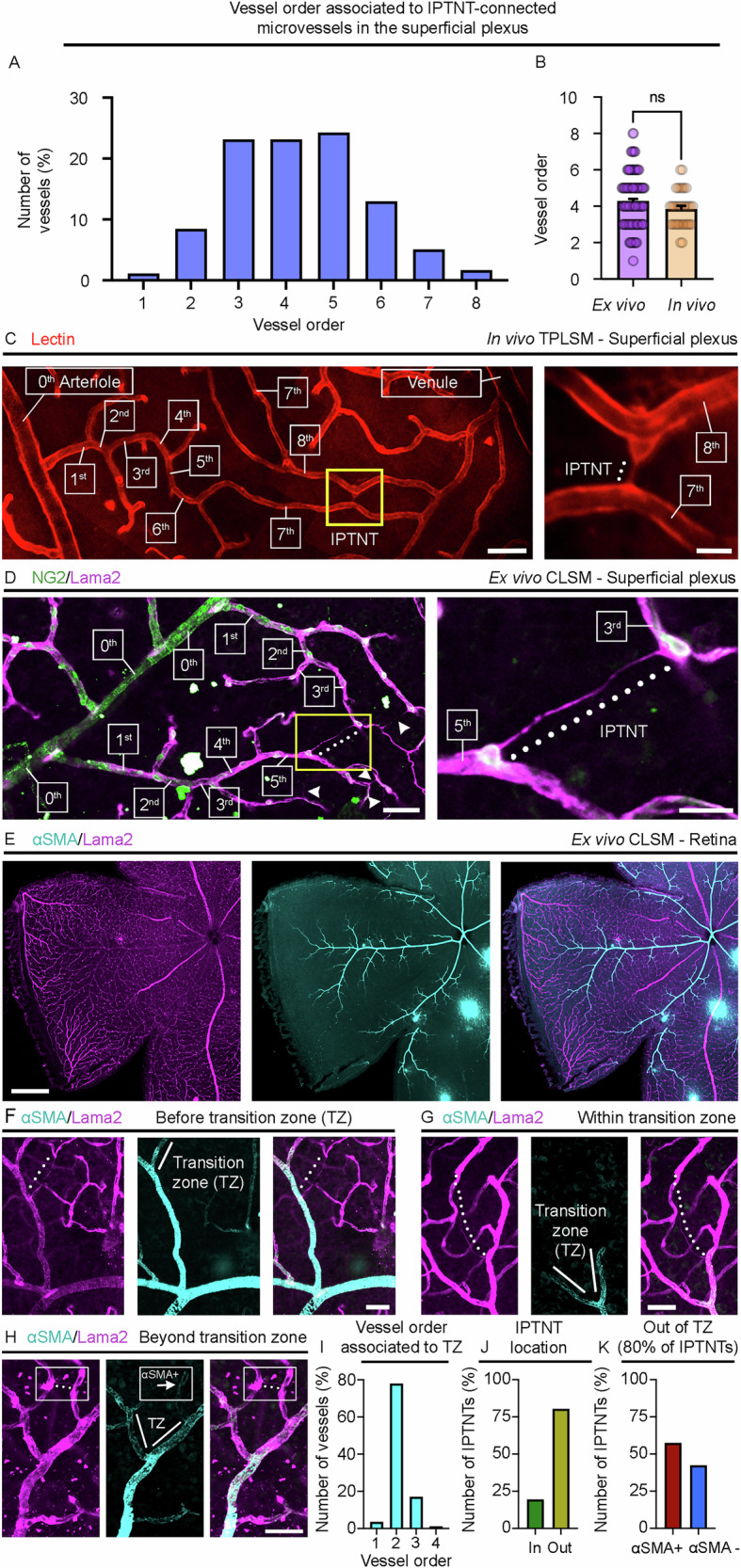
Location of the IPTNTs in the retinal superficial plexus. **A**–**D** IPTNTs located in the retinal superficial plexus connect 1st–8th-order capillaries (**A**, *n* = 177 capillaries, *N* = 4 mice) with an average of ~4th order in vivo (**B**, *n* = 33 capillaries, *N* = 3 mice, two-tailed Mann–Whitney *U* test, ns not significant, data are presented as mean values +/− SEM; **C** tiling montage with two-photon laser scanning microscopy, TPLSM, after an intravitreal injection of lectin; replicated four times in two mice) and ex vivo (**B**, *n* = 177 capillaries, *N* = 4 mice; **D** tiling montage acquired with confocal laser scanning microscopy, CLSM, of the superficial plexus labeled with NG2, green, and Lama2, purple). Numbers depict the vessel’s order, and dotted lines show an IPTNT. Arrowheads indicate other examples of IPTNTs. **E** One example of a flat-mounted retina labeled with antibodies against αSMA (cyan) and Lama2 (purple) (replicated in 4 mice). **F**–**I** Examples of an arteriole-capillary transition zone (TZ) identified as the zone where αSMA expression changes from high to low levels, showing IPTNTs (dotted line) located before (**F**), within (**G**), and beyond the TZ (**H**; arrow points to expression of αSMA in an IPTNT-connected pericyte located beyond the TZ). TZ was located in 1st–4th-order capillaries (**I**, *n* = 82 capillaries, *N* = 4 mice). **J**, **K** IPTNTs were mainly located beyond the transition zone (**J**, *n* = 66 pericytes beyond the transition zone, out; *n* = 16 pericytes before/within the transition zone, in, *N* = 4 mice), and 57% of those presented low αSMA expression (**K**, *n* = 38 αSMA+ pericytes, *n* = 28 αSMA- pericytes, *N* = 4 mice). Scale bar in (**C**, **D**, **F**–**H**) = 50 μm; in (**E**) = 500 μm; in (**C**) inset = 10 μm; and in (**D**) inset = 20 μm. Source data are provided as a Source Data file.

As IPTNTs connect functionally-opposite neuronal areas, with slower capillaries supplying to ON RGCs, which are inactive in the dark, and with faster capillaries supplying to OFF cells, which are activated by the absence of light, we wondered whether the change of the neuronal responses via light stimulation could modify the blood flow pattern. Accordingly, we presented a flash light stimulus and quantified blood cell flux and velocity in IPTNT-connected retinal capillaries of the superficial plexus where the RGCs are located, in living mice (Fig. 3A and Movie S3). IPTNTs present a lower density in this plexus compared to the intermediate and deep one, probably because of the lower neuronal density in this retinal layer (~ 50,000 RGCs) related to the inner (~ 500,000 bipolar cells) or the outer nuclear layer (~ 7 million photoreceptors)^26,44^. Before light stimulation, IPTNT-connected capillaries showed significant differences in blood flow (Fig. 3B–D) and blood cell velocity (Supplementary Fig. 4A–C), with one faster and one slower flow capillary in most pairs. Light stimulation induced a selective decrease of blood cell flux/velocity and constriction in most faster capillaries and an increase of flux/velocity and dilation in most slower capillaries (Fig. 3E–J, Supplementary Fig. 4D–F, and Movie S4). This specific light-evoked change in the blood flow pattern was different in non-connected capillaries, with an overall increase in their blood flow and velocity, similar to the global functional hyperemia previously reported in the retina (Supplementary Fig. S5A–H)^45^. Generally, slower capillaries responded to light more rapidly than faster capillaries (Supplementary Fig. 6A, B). This led to a reduction of the intercapillary blood flow/velocity difference (Fig. 3G and Supplementary Fig. 4F) and a homogenization of blood flow/velocity patterns after light stimulation (Fig. 3E and Supplementary Fig. 4D), which subsequently returned to baseline values following stimulation (Fig. 3K). Importantly, blood flow changes did not result from random variations as vascular changes were not detected in control experiments without light (Supplementary Fig. 6C, D). The pericyte soma and the IPTNT endfoot were not particularly associated with faster/slower capillaries (Supplementary Fig. 6E, F). Due to the reported differences in vascular responses depending on the pericyte position within the vascular tree and the expression of α-smooth muscle actin (αSMA)^32,46^, we analyzed the location of pericytes expressing tunneling nanotubes as well as their αSMA expression. Importantly, rapid fixation methods are essential for proper αSMA detection post-mortem in the retina due to the quick actin depolymerization after death^24,47–49^. Hence, differences between fixation protocols may introduce a large variability in αSMA expression between studies in the retina^24,47–50^. In our study, we performed quick fixation via transcardial perfusion (see “Methods”). The retinal superficial plexus was formed by 0th-order radial retinal arterioles branching from the central retina artery (CRA) and 1st–8th-order microvessels (Fig. 4A–E). IPTNTs were primarily located beyond the arteriole-capillary transition zone (TZ), identified as the zone where αSMA expression changes from high to low levels (Fig. 4F–H; see “Methods”). Thus, 20% of IPTNTs were located before/within the TZ (Fig. 4F, G, I, J) and 80% of IPTNTs were located beyond the TZ (Fig. 4H–J). We found that 57% of these IPTNTs beyond the TZ connect pericytes with at least one of them expressing some level of αSMA (Fig. 4K). This is in accordance with previous studies, where 70% of the IPTNTs connect at least one αSMA-positive pericyte in the superficial plexus^24^. The proportion of IPTNTs to capillary segments in the whole retina was ~1 to 4 (174 IPTNT/mm^2^ vs. 770 segments/mm^2^, Supplementary Fig. 7A; ~1 to 6 in the superficial plexus). In most pairs, the IPTNT connected capillaries fed by different vessels (different feeding vessel 87% vs. same feeding vessel 13%, Supplementary Fig. 7B) and those fed by the same vessel were arranged more in-parallel (in-parallel 61% vs. in-series 39%, Supplementary Fig. 7C, D). IPTNTs are present in the brain, but they are less abundant and shorter than in the retina (Supplementary Fig. 7E–G; please note the volume units in Supplementary Fig. 7F). Overall, these results suggest that pericytes connected via IPTNTs could be involved in the regulation of light-evoked changes in blood flow patterns.

### IPTNTs regulate blood flow patterns

**Fig. 5:**
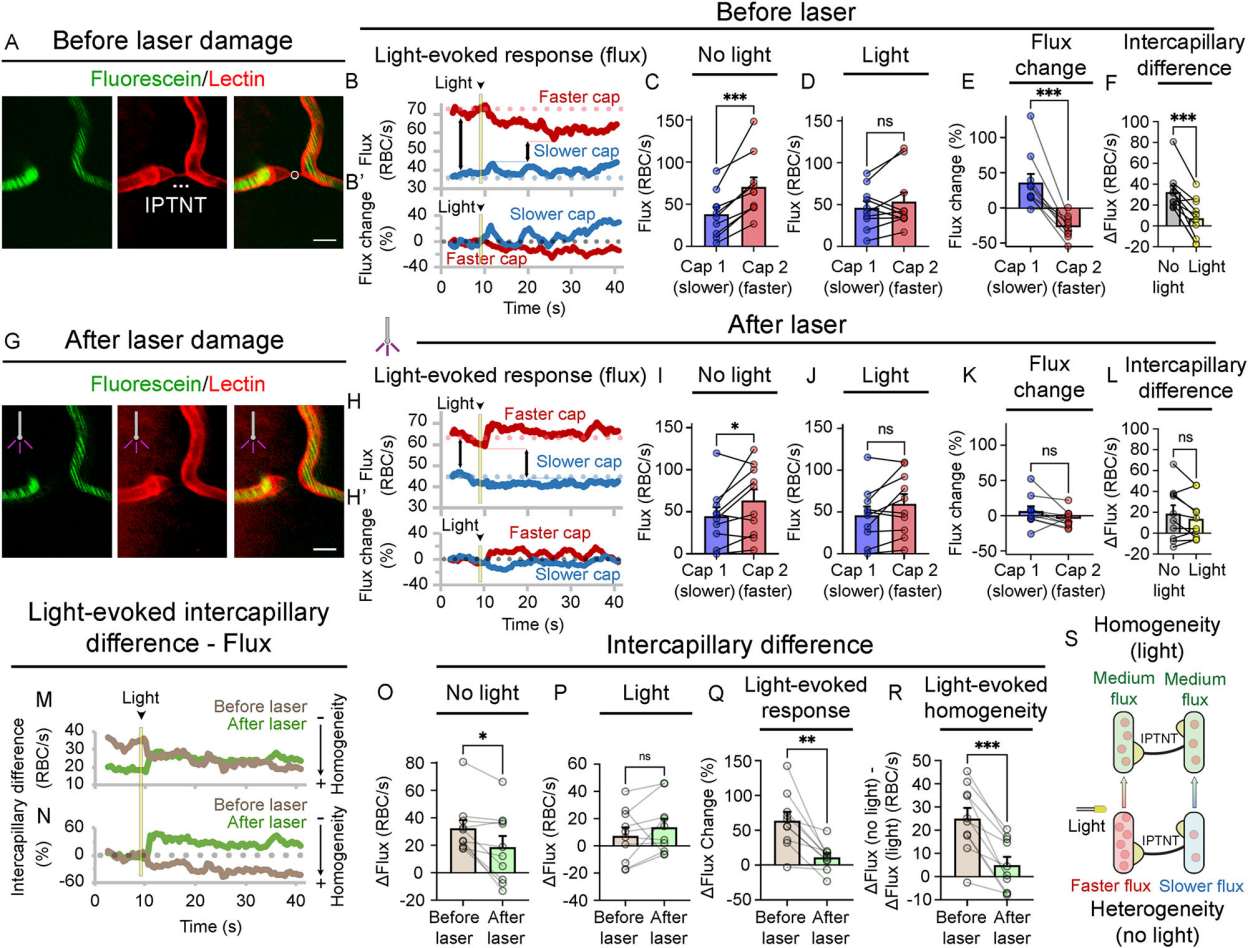
Targeted IPTNT ablation disrupts blood flux patterns. **A** IPTNT before laser ablation (circle; replicated 14 times in 9 mice). **B**, **B’** Light-evoked flux response (**B**) and change (**B’**) of slower (blue) and faster capillaries (red) (arrowheads indicate light) (average of 10 capillaries per trace, *N* = 6 mice; double-headed arrows indicate intercapillary difference). **C**–**F** Maximum response graphs show that, before light, IPTNT-connected vessels presented a significant flux difference (**C**), which disappeared after light (**D**) (*n* = 20 capillaries, *N* = 6 mice, linear mixed-effect model analysis, ***p = 0.0004, ns not significant) by selective flux increase in slower capillaries and decrease of flux in faster vessels (*n* = 20 capillaries, *N* = 6 mice, linear mixed-effect model analysis, ***p = 0.0007) (**E**). Intercapillary difference shows light-evoked homogeneity (*n* = 20 capillaries, *N* = 6 mice, two-tailed paired Student’s *t*-test, ***p = 0.0004) (**F**). **G**–**H’** IPTNT ablation (**G**) eliminated the ability of homogenization after light (**H**, **H’**, average of 10 capillaries per trace, *N* = 6 mice; double-headed arrows indicate intercapillary difference). **I**–**L** IPTNT ablation reduced the flux difference between connected capillaries before light (**I**) with no difference after light (**J**) (*n* = 20 capillaries, *N* = 6 mice, linear mixed-effect model analysis, *p = 0.044, ns: not significant), and eliminated the ability of slower capillaries to increase their flux and faster capillaries decrease it after light (*n* = 20 capillaries, *N* = 6 mice, linear mixed-effect model analysis, ns not significant) (**K**). Accordingly, intercapillary difference was similar before and after light (*n* = 20 capillaries, *N* = 6 mice, two-tailed paired Student’s *t*-test, ns not significant) (**L**). **M**, **N** Graphs comparing flux difference (**M**) and percent-of-change difference (**N**) between connected capillaries before (brown) and after (green) IPTNT ablation. IPTNT damage eliminated flux homogenization after light (average of 10 capillaries per trace, *N* = 6 mice). **O**–**R** IPTNT ablation led to a reduction of the intercapillary flux difference before light (**O**) but not after light (**P**), and to a reduced light-evoked response (**Q**) and homogeneity (**R**) (*n* = 20 capillaries, *N* = 6 mice, two-tailed paired Student’s *t*-test, *p = 0.011, **p < 0.002, ***p = 0.0001, ns not significant). **S** Drawing summarizing light-evoked changes in blood flow patterns. **A** and **G** are the sum of multiple frames. Scale bars in (**A**), (**G**) = 10 μm. All data are presented as mean values +/− SEM. Source data are provided as a Source Data file.

**Fig. 6:**
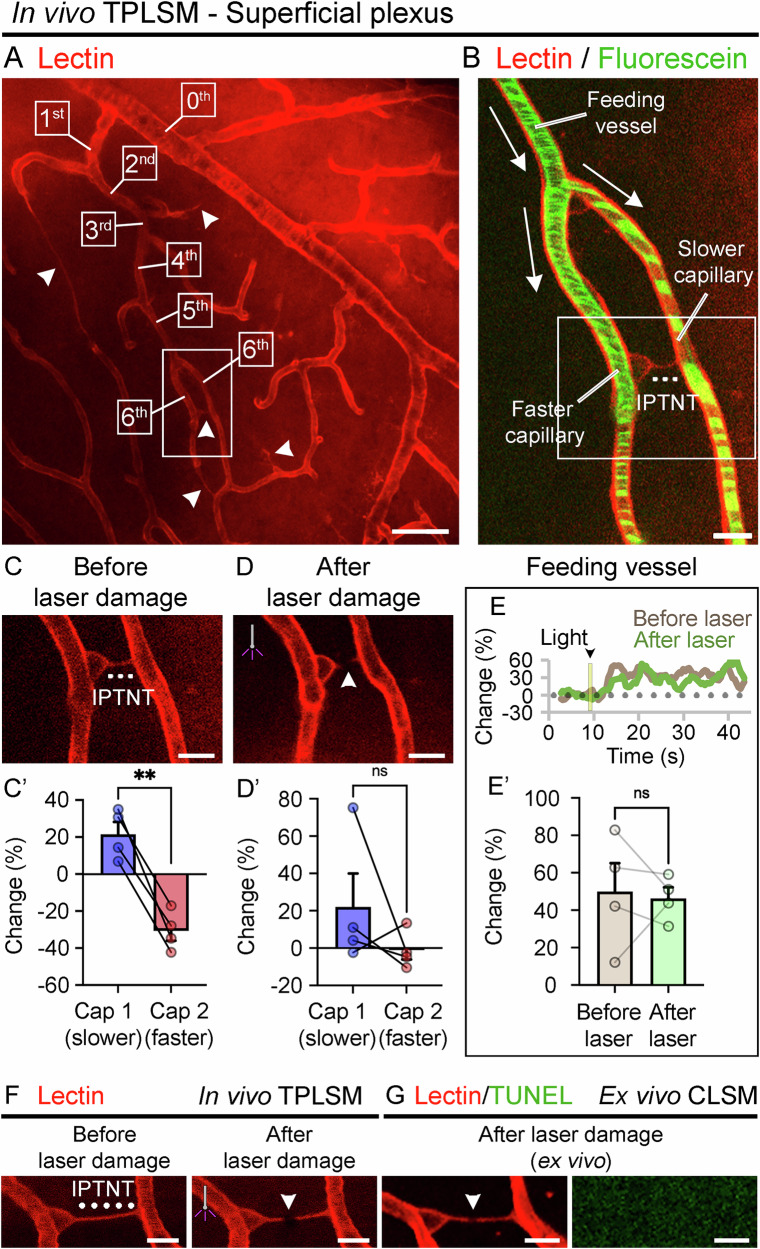
IPTNT-laser ablation does not affect the upstream feeding-vessel function. **A** In vivo TPLSM imaging of a retina labeled with intraocular injection of TRITC-lectin. Numbers depict the vessel’s order, and arrowheads show IPTNTs. **B** Inset in (**A**) of two capillaries connected by an IPTNT (dotted line) and fed by a common upstream vessel or feeding vessel (arrows indicate blood flow direction, labeled with an IP injection of fluorescein, green). Replicated 4 times in 3 mice. **C**–**D** Before (**C**, **C’**) and after (**D**, **D’**) laser damage of the IPTNT in (**B**) (arrowhead points out the rupture site in (**D**)). Laser-induced IPTNT damage eliminated the ability of flux homogenization after light stimulation (*n* = 8 capillaries, *N* = 3 mice, linear mixed-effect model analysis, **p = 0.003, ns not significant). **E** The feeding vessels were not dysfunctional after IPTNT depletion, presenting similar light-evoked functional hyperemia (**E**) before and after IPTNT-laser ablation (**E’**, *n* = 4 vessels, *N* = 3 mice, two-tailed paired Student’s *t*-test, ns not significant). **F**, **G** In vivo ablation of an IPTNT labeled with intravitreal lectin (red) by laser (**F**, left, before ablation; right, after ablation; arrowhead points out the rupture site) does not induce apoptosis in pericytes identified with a TUNEL assay in ex vivo flat-mounted retinas (**G**, green; same IPTNT as in (**F**); arrowhead points out the rupture site). Replicated 7 times in 4 mice. Scale bar in (**A**) = 50, (**B**–**D** and **F**, **G**) = 10 μm. All data are presented as mean values +/− SEM. Source data are provided as a Source Data file.

To confirm if the formation of blood flow patterns is truly regulated by IPTNTs, we analyzed light-evoked responses in the same capillary pairs before (Fig. 5A–F and Supplementary Fig. 8A–E) and after damaging their associated IPTNTs using a laser (Fig. 5G–L and Supplementary Fig. 8F–J). Before damage, IPTNT-connected capillaries displayed heterogeneous blood flow and velocity prior to light stimulation (Fig. 5B, C and Supplementary Fig. 8A, B). Faster and slower capillaries showed light-evoked decreased and increased flow/velocity, respectively (Fig. 5B–E and Supplementary Fig. 8A–D), which led to less intercapillary difference (Fig. 5F and Supplementary Fig. 8E). Laser-ablation of IPTNTs (Fig. 5G) reduced heterogeneity before light (Fig. 5H, I and Supplementary Fig. 8F, G) and impaired the ability of faster capillaries to decrease their flow/velocity and slower capillaries to increase their flow/velocity after light stimulation (Fig. 5H, H’, J, K, Supplementary Fig. 8F, F’, and Supplementary Fig. 8H, I). Indeed, there was no light-evoked change in intercapillary difference (Fig. 5L and Supplementary Fig. 8J). We found that IPTNT-laser ablation led to abnormal intercapillary flow and velocity patterns (Fig. 5M, N and Supplementary Fig. 8K, L), with a reduced flux heterogeneity prior to light stimulation (Fig. 5O and Supplementary Fig. 8M), a reduced light-evoked response (Fig. 5P, Q and Supplementary Fig. 8N, O), and a loss of the ability to homogenize blood cell flux and velocity after stimuli (Fig. 5R and Supplementary Fig. 8P; green trace vs. brown trace in Fig. 5M, N and Supplementary Fig. 8K, L), which suggests IPTNTs as regulators of flow homogenization during light stimulation (Fig. 5S). IPTNT-laser ablation mainly changed the formation of blood flow patterns with less effect on light-evoked net changes, including the number of capillaries with light-evoked increases in blood flux/velocity (i.e., functional hyperemia; before IPTNT damage: 10/20 capillaries for blood flux and 8/22 capillaries for velocity; after IPTNT damage: 9/20 capillaries for blood flux and 11/22 capillaries for velocity) (Supplementary Fig. 9A–L). Accordingly, individual capillaries still responded to light after laser damage, but slower capillaries did not specifically increase their blood cell flux and velocity (net sum ~0; individual points in Fig. 5K and Supplementary Fig. 8I, blue bar) and faster ones did not always decrease it (net sum ~0; individual points in Fig. 5K and Supplementary Fig. 8I, red bar), which resulted in the formation of abnormal blood flow patterns. Importantly, IPTNT-laser ablation did not affect functional hyperemia in upstream vessels (Fig. 6A–E) or damage pericytes (Fig. 6F, G). Collectively, these results demonstrate that IPTNTs are essential regulators of blood flow patterns in the retina and play a role in controlling flow homogenization during light stimulation.

### Ischemia-reperfusion injury leads to disruption of blood flow patterns

**Fig. 7:**
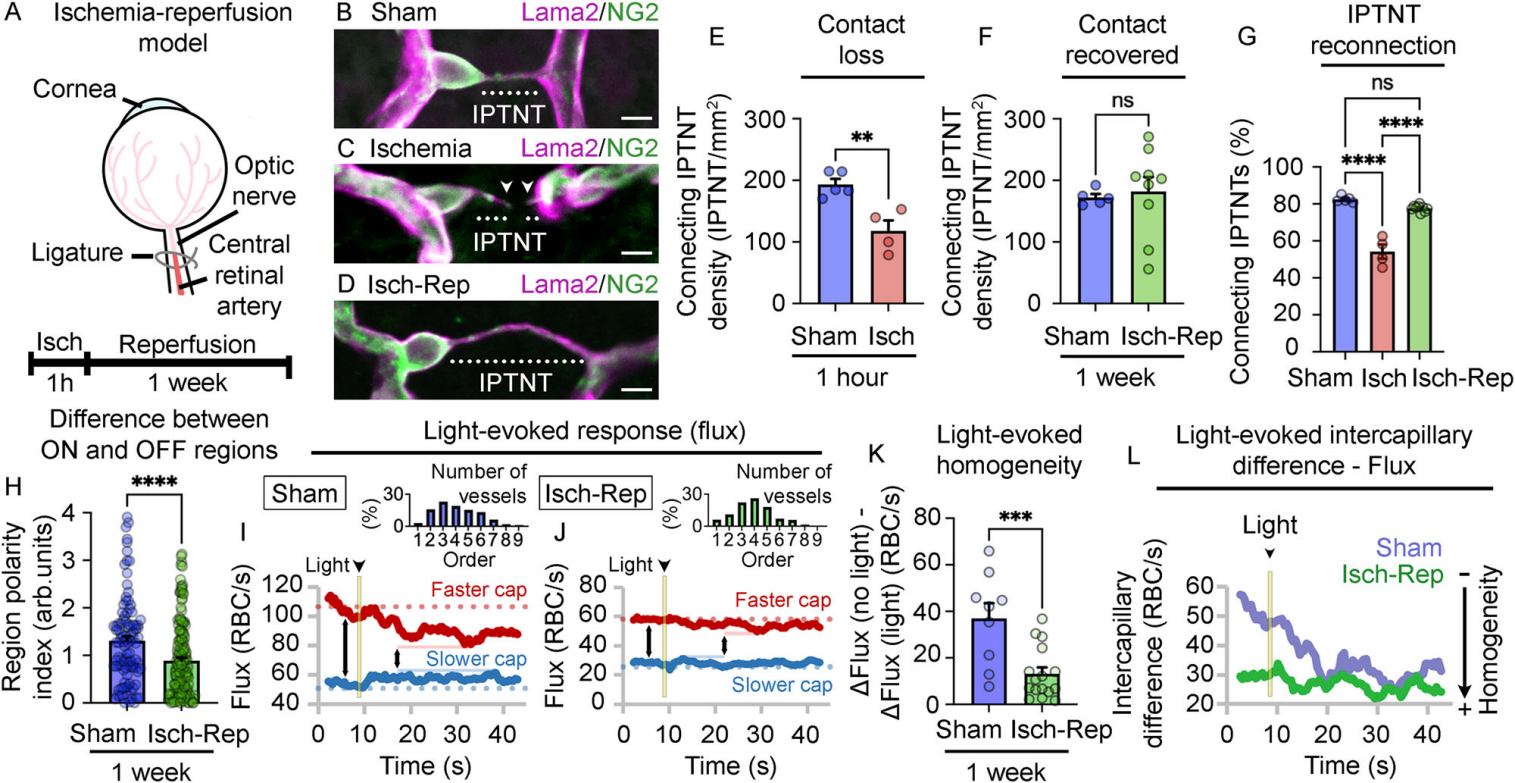
Ischemia-reperfusion injury leads to the loss of light-evoked flux homogeneity. **A** Ischemia-reperfusion model to transiently interrupt the retinal blood supply. **B**–**D** Flat-mounted retinas labeled with antibodies against Lama2 (purple) and NG2 (green) showed more ruptured IPTNTs (**C**, arrowheads) after 60-min ischemia (**C**) than sham retinas (**B**). IPTNTs connect distal vessels after one-week reperfusion (Isch-Rep) (**D**). **E**–**G** Density analysis confirmed a decrease in the number of IPTNTs connecting capillaries in ischemic retinas relative to sham-operated controls (*n* = 2939 IPTNTs, *N* = 4 ischemia mice; *n* = 4152 IPTNTs, *N* = 5 sham-operated mice, two-tailed Student’s *t*-test, **p = 0.004) (**E**). One-week reperfusion re-established the total number of connecting IPTNTs compared to sham-operated controls (*n* = 7319 IPTNTs, *N* = 9 Isch-Rep mice; *n* = 4102 IPTNTs, *N* = 5 sham-operated mice, two-tailed Student’s *t*-test, ns not significant) (**F**). Percent graph confirms a reperfusion-dependent IPTNT reconnection (*n* = 4152 IPTNTs, *N* = 5 1hr-sham-operated mice; *n* = 2939 IPTNTs, *N* = 4 1hr-ischemia mice; *n* = 7319 IPTNTs, *N* = 9 Isch-Rep mice, one-way ANOVA Tukey’s test for multiple comparisons, ****P < 0.0001, ns not significant) (**G**). **H** Ex vivo analysis demonstrated a reduction of the difference of polarity index between areas during Isch-Rep relative to sham-operated controls (*n* = 202 areas, *n* = 364 α-cells, *N* = 6 mice in sham group; *n* = 332 areas, *n* = 555 α-cells, *N* = 6 mice in isch-rep group, two-tailed Mann–Whitney *U* test, ****p < 0.0001). **I**, **J** Light-evoked capillary flux changes for sham (**I**) and ischemia-reperfusion (**J**) mice. Ischemia-reperfusion eliminated the ability of slower capillaries to increase their flux (blue) and of faster capillaries to decrease it (red) after light (arrowhead) (average of 16 capillaries per trace, *N* = 4 mice) compared to sham mice (average of 9 capillaries per trace, *N* = 3 mice; double-headed arrows indicate intercapillary difference). Histograms show the order of the capillaries where IPTNTs were located for each condition. **K**, **L** Light-evoked homogeneity graph (**K**) and flux difference (**L**) between connected capillaries show a reduced ability for ischemia-reperfusion mice to homogenize flow of IPTNT-connected capillaries after light (green) relative to sham controls (blue) (*n* = 18 capillaries, *N* = 3 mice in sham group; *n* = 32 capillaries, *N* = 4 mice in isch-rep group, two-tailed Student’s *t*-test, ***p = 0.0007). Scale bars in (**B**–**D**) = 5 μm. All data are presented as mean values +/− SEM. Source data are provided as a Source Data file. Arb. units arbitrary units.

Next, we interrogated whether broad rupture of IPTNTs modifies retinal blood flow patterns. For this purpose, we used a retinal ischemia-reperfusion injury model previously shown to induce widespread IPTNT damage^23^. One hour of retinal ischemia was followed by one week of reperfusion (Fig. 7A). Transient ischemia promoted IPTNT rupture, assessed one hour after the beginning of the ischemic event (Fig. 7B, C and Supplementary Fig. 10A, B), and, unexpectedly, at one week following reperfusion, there was evidence of IPTNT reconnecting pericytes in distal capillaries (Fig. 7D–G and Supplementary Fig. 10A and C). Based on this, we considered whether IPTNTs reconnected appropriately (i.e., supporting functionally-opposite neuronal regions) during reperfusion by assessing opposite-functional neuronal areas surrounding IPTNT-connected capillaries. We found a reduction in the difference in polarity between opposite-functional areas surrounding IPTNTs across the retina (Fig. 7H), which was not due to changes in the number of ON and OFF RGCs around IPTNTs (Supplementary Fig. 10D, E). Nevertheless, ischemia-reperfusion injury led to a significant reduction in the number of RGCs in areas without IPTNTs (Supplementary Fig. 10F) as previously reported^51,52^. Reduction in the difference in polarity between opposite-functional areas was associated with a loss of light-evoked homogeneity response compared to sham-operated controls (Fig. 7I–L and Supplementary Fig. 11A–D; green trace vs. blue trace in Fig. 7L and Supplementary Fig. 11D).

**Fig. 8:**
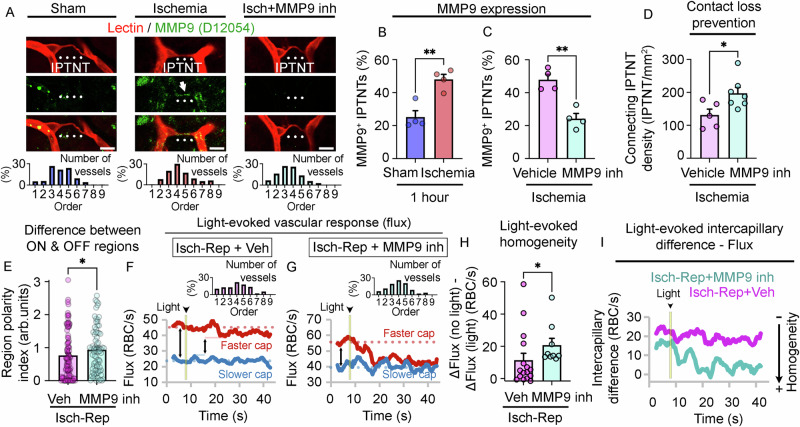
Preservation of IPTNTs during ischemia maintains light-evoked blood flow homogenization. **A** Flat-mounted retinas labeled with lectin (red) and MMP9-activation indicator D12054 (green) showing an MMP9 + IPTNT (arrow) after 60-min ischemia (middle) compared to a sham control (left). MMP9 activation during ischemia was prevented with an intravitreal injection of MMP9 inhibitor I (20 µM), thirty minutes before ischemia (right). All images have identical settings. Histograms show the order of the capillaries where the IPTNTs were located for each condition. **B**–**E** Quantitative analysis confirmed an increase in the number of MMP9+ IPTNTs in ischemic retinas relative to sham-operated controls (*n* = 179 IPTNTs, *N* = 4 ischemia mice; *n* = 182 IPTNTs, *N* = 4 sham-operated mice, two-tailed Student’s *t*-test, **p = 0.003) (**B**) and the prevention of MMP9 activation at IPTNTs via MMP9 inhibitor I (*n* = 179 IPTNTs, *N* = 4 ischemia + MMP9 inhibitor mice; *n* = 182 IPTNTs, *N* = 4 ischemia+vehicle mice, two-tailed Student’s *t*-test, **p = 0.002) (**C**), which prevented the density loss of IPTNTs connecting capillaries (*n* = 6622 IPTNTs, *N* = 7 ischemia + MMP9 inhibitor mice; *n* = 4932 IPTNTs, *N* = 5 ischemia + vehicle mice, two-tailed Student’s *t*-test, *p = 0.029) (**D**) and preserved the polarity index between IPTNT-connecting neuronal areas (*n* = 192 areas, *n* = 298 α-cells, *N* = 5 mice in vehicle group; *n* = 154 areas, *n* = 236 α-cells, *N* = 4 mice in MMP9 inhibition group, two-tailed Mann–Whitney *U* test, *p = 0.042) (**E**). **F**, **G** MMP9 inhibition preserved the ability of faster capillaries to decrease their flux (red) and slower capillaries to increase it (blue) after light (**G**, average of 9 capillaries per trace, *N* = 4 mice) compared to Isch-Rep + vehicle mice (**F**, average of 16 capillaries per trace, *N* = 4 mice; double-headed arrows indicate intercapillary difference). Histograms show the order of the capillaries where the IPTNTs were located for each condition. **H**, **I** Light-evoked homogeneity graph (**H**) and flux difference (**I**) between connected capillaries show that the prevention of IPTNT rupture with MMP9 inhibitors (cyan) prevented the loss of the ability to homogenize flow after light (arrowhead) relative to vehicle controls (pink) (*n* = 32 capillaries, *N* = 4 mice in vehicle group; (*n* = 18 capillaries, *N *= 4 mice in MMP9 inhibition group, two-tailed Mann–Whitney *U* test, *p = 0.020). Scale bars in (**A**) = 10 μm. All data are presented as mean values +/− SEM. Source data are provided as a Source Data file. Arb. units arbitrary units.

To evaluate whether preventing ischemia-induced rupture of IPTNTs maintains coupling between capillary flow and RGC ON/OFF polarization, we applied the matrix metalloproteinase-9 (MMP9) inhibitor, C_27_H_33_N_3_O_5_S^53^. MMP9 is an enzyme produced by pericytes that is activated during ischemic conditions and can mediate the disruption of the vascular basal membrane^53^. As IPTNTs are formed from the pericyte basal membrane^23^, we expected that MMP9 inhibition could prevent the rupture of IPTNTs during an ischemic insult. To test this assumption, we used the fluorescent probe D12054 as an indicator of MMP9 activation (Fig. 8A)^53^. We found that ischemia increased MMP9 activation in IPTNTs compared to sham animals (Fig. 8A, B). The intravitreal injection of an MMP9 inhibitor 30 min prior to ischemia decreased MMP9 activation (Fig. 8A and C) and prevented IPTNT rupture in the retina when compared with vehicle injection (Fig. 8D and Supplementary Fig. 12A). Importantly, the preservation of IPTNTs during ischemia conserved coupling between capillary flow and RGC ON/OFF polarization (Fig. 8E), which was not due to changes in the number of ON and OFF RGCs around IPTNTs (Supplementary Fig. 12B, C). Areas around IPTNTs presented higher RGC densities than regions without IPTNTs (Supplementary Fig. 12D, E), suggesting localized neuroprotective effects that coincide with a maintained light-evoked blood flow homogenization (Fig. 8F–I and Supplementary Fig. 13A–D; pink trace vs. cyan trace in Fig. 8I and Supplementary Fig. 13D). These results suggest that widespread damage of IPTNTs leads to the loss of optimal blood flow patterns in the retina.

## Discussion

Although oxygen supply occurs primarily at the capillary level^54^, relatively little is known of single-capillary function and the interplay of this with neuronal activity. Consequently, there are major gaps in our understanding of the regulation of blood supply to the CNS. The retina is a developmental extension of the forebrain and shares many features in common with the brain, including in terms of neuronal organization and key characteristics of the vascular system. Accordingly, studies of retinal neurovascular function have provided valuable insights into the brain. In this study, we have demonstrated that pericytes, acting via IPTNTs, regulate blood flow patterns in the retina, coupling neuronal activity with supply. This finding may have important implications for blood flow regulation in the CNS.

Previous studies have reported the existence of blood flow patterns in the brain and retina^4,8,13,21,55–57^. Before stimulation, capillary blood flow is heterogeneous, with some vessels showing slower blood flow and others faster flow. After neuronal activation, the difference in blood flow between vessels is reduced, and a homogeneous pattern is established^4,6–8,13–15,17,18,55,56,58^. Although the purpose of heterogeneous blood flow patterns in the CNS is not well understood, in vivo experiments^5^ and computational models^6,59–61^ have suggested that homogeneous blood flow patterns may facilitate the delivery of oxygen and glucose during neuronal activation. However, it is important to note that, in the CNS, neuronal subtypes have varying levels of basal activity under unstimulated conditions. Accordingly, our results suggest that both blood flow homogeneity and heterogeneity are necessary to obtain the appropriate oxygen supply. A case in point is ON and OFF RGCs, which are activated by light and the absence of light, respectively. In this example, neurovascular coupling assumes that blood supply should match the demands of retinal neurons that are active *before* light stimulation, as well as neurons that are active *during* light stimulation. Our findings indicate that groups of OFF RGCs, which are activated by the absence of light, are supplied by faster capillaries than groups of ON RGCs, which are inactive in the dark. Light activates ON RGCs, leading to greater flux in their slow capillaries, and deactivates OFF RGCs, leading to a decreased flux in their fast capillaries. Further studies should confirm whether IPTNTs located in other retinal plexuses also regulate the blood supply of neuronal groups involved in transmitting a particular visual information.

The mechanisms by which this neurovascular coupling occurs have been elusive. Although basal anatomical differences in diameter between capillaries can account for the large difference in flow under unstimulated conditions^31^, they cannot explain active flow homogenization after neuronal stimulation. The current neurovascular model suggests that vasoactive products derived from neurons or glial cells can modify capillary diameter by activating receptors on pericytes^1,19,20,23,25,47,49,62–65^, changing intracellular calcium concentration and, subsequently, the contractile state of pericytes^66–70^. Electrically coupled endothelial cells and pericytes propagate signals upwards, leading to changes in smooth muscle cells and pericytes of feeding arterioles with larger contractile/relaxing machinery^50,71–78^. This model describes a system where the whole vascular tree would show increased blood flow after neuronal activity. However, it does not describe how stimuli may raise different responses in each vessel, leading to blood flow homogeneity. Accordingly, computational models have predicted a finer blood flow regulation at the capillary level, with some microvessels dilating and others constricting^4,55,56,59,60^. Our findings demonstrate that the generation of blood flow patterns in the retina resides in pericytes acting via IPTNTs on capillary pairs that supply functionally-opposite neuronal areas, thereby coupling neuronal activity with blood supply. Critically, further studies are needed to understand which substances derived from neurons and glial cells may activate potential receptors at the IPTNTs, modifying gap junction conductivity, and consequently, calcium traveling between pericytes. This is notably important for pericytes as their contractibility depends on intracellular calcium^47^. Particularly, the IPTNT endfoot is an important target as it shows abundant gap junctions as well as energy consumption suggested by the large number of mitochondria^23^. Gap junctions are active channels modulated by substances released during light stimulation, such as adenosine^79^, dopamine^79–81^, glutamate^82^, ATP^83^, or nitric oxide (NO)^74^, which may activate associated channels/G-protein coupled receptors (GPCRs) in IPTNT endfeet and pericytes^84,85^. GPCRs require energy for proper function^85^, and their activation will induce changes in the membrane potential and phosphorylation/nitrosylation of gap junctions and, consequently, their conductance as previously reported^86^. For example, cAMP-dependent phosphorylation and NO-dependent nitrosylation of gap junctions improve the electrical communication between retinal neurons^80^ and pericytes^74^, enhancing conductance. Accordingly, it is plausible that neuronal- and/or glial-derived substances may modify gap junctions at the IPTNT-endfoot, changing calcium communication and calcium concentration in IPTNT-connected pericytes, which would alter their contractility state and, consequently, blood flow patterns. Experiments with selective blockers and conditional knockout mice for receptors and their downstream pathways will be very valuable in elucidating the IPTNT-dependent mechanism that leads to the formation of blood flow patterns in the retina.

Our study focused on capillaries located in the retinal superficial plexus, which is formed by microvessels that include IPTNT-connected pericytes located before/within the transition zone (with robust αSMA expression; 20% of the IPTNTs) and beyond it (with low αSMA expression; 80% of the IPTNTs)^46,47^. Although pericytes located at different vessels show αSMA variability, previous studies in living mice have reported rapid vascular responses not only before or within the transition zone^50,62,64,87^, but also beyond it^64,88^. Accordingly, in an in vivo study where the vascular zones were differentiated, it was reported that thin-strand pericytes responded ~4 s after the beginning of the stimulus^88^. Moreover, Rungta et al. showed changes in calcium of mural cells and the RBC velocity of 6–8th-order capillaries ~3 s after the beginning of the stimulus^64^. These findings are in accordance with our results, where we recorded similar vascular responses across the superficial plexus of the retina in living mice after presenting light stimuli. Furthermore, we found that IPTNT-connected capillaries showed different response times. This disparity in time probably reflects differences in the mechanism regulating intracellular calcium changes in each pericyte, such as the redistribution of intracellular calcium between connected pericytes via gap junctions, or the activation/deactivation of membrane receptors by products released from the ON- and OFF-areas. Thus, it is plausible that a different combination of mechanisms regulates the intracellular calcium in each connected pericyte, modifying the proportion of activated myosin light chain kinase (MLCK) and myosin light chain phosphatase (MLCP), and leading to disparate response times^89^. Future studies should determine whether rapid vascular responses recorded at IPTNT-connected high-order capillaries in the retina of living mice are initiated in upstream arterioles/proximal pericytes with larger αSMA expression, and if they are also involved in the differences in the time of response found between slower and faster capillaries. Importantly, we should also consider the impact of the anesthetics on our results. Ideally, neurovascular coupling experiments should be conducted in awake animals to avoid potential neuronal inhibition and vascular alterations resulting from anesthetic^90^. However, due to eye movements and ethical concerns related to keeping the animals awake during TPLSM retinal imaging, it is challenging to perform these experiments in conscious mice. To reduce the anesthetic effects in our in vivo experiments, we used ketamine instead of isoflurane. Isoflurane is a potent vasodilator that may prevent further vasodilation during light stimulation^90^, as well as an inhibitor of RGC function^91^. On the other hand, although ketamine may alter metabolism in the somatosensory and auditory cortices^92^, it does not inhibit RGC function and induces minimal cardio-respiratory depression^90,91,93^. Additionally, it is important to mention that ketamine at high concentrations may block L-type calcium channels^94^. Although in our study we do not use these high concentrations^95^, and previous studies have shown functional L-type calcium channels in mice anesthetized with ketamine^88^, we may expect larger light-evoked responses in awake mice.

Previous studies have suggested that basal blood flow is sufficient to supply oxygen and nutrients during neuronal activation^96^. It is in accordance with our findings, where we show that no overall changes occurred after light stimulation, but instead, a redistribution of blood flow. Thus, precise regulation of blood flow patterns ensures that the right amount of oxygen is delivered to each group of neurons, homogenizing during functional activation and correlating with the stimulus intensity^13,26^. Therefore, if blood flow patterns do not change appropriately during neuronal activity, hypoxia may occur^6,97^. Paradoxically, it has been reported that very high blood flow rates are inefficient for oxygen delivery as erythrocytes do not have sufficient time to release oxygen to the tissue^98^. Thus, large heterogeneity in blood flow may cause harm as areas of under-perfused vessels receive insufficient oxygen and glucose, whilst neurons in areas of over-perfused capillaries receive an excess of oxygen and energy substrates^6,99^. Accordingly, alteration of blood flow patterns has been reported in neurodegenerative diseases such as Alzheimer’s disease^99–101^ and stroke^15,102,103^. This aligns with our results, which suggest that the disruption of blood flow patterns after ischemia may be due to the loss of IPTNTs in the acute stage and the abnormal connection of IPTNTs following reperfusion, and that protection of IPTNTs from ischemia injury with MMP9 inhibitors could preserve blood flow patterns. Here, we must also consider that our pharmacological experiments could preserve blood flow patterns via beneficial effects on other cells.

Additional research should verify if blood flow patterns are also regulated by IPTNTs connecting capillaries in the brain. In contrast to our findings in the retina, a recent study in the mouse barrel cortex concluded that hemodynamic changes of single vessels were not accurate indicators of surrounding neuronal activity^104^. The discrepancy between results may be due to the fact that our study focused on vessels connected with IPTNTs and their associated neuronal areas. On the other hand, Martineau et al. did not identify vessels connected by IPTNTs or functionally-opposite neuronal regions, which are important indicators for the location of IPTNTs^104^. Furthermore, it is also possible that not all regions of the brain present IPTNTs. Thus, specific zones of the CNS may be more convenient for the study of the IPTNTs. Particularly, the visual cortex and superior colliculus present orientation-selective neurons, which are preferentially activated by stimuli moving through one orientation, and inactivated when stimuli move orthogonally to it^105,106^. Similar to the retina, IPTNTs may connect vessels supplying groups of neurons with opposite orientation selectivity, which could be impaired in disease. In vivo studies with transgenic mice expressing fluorescent IPTNTs are needed to clarify whether there is a general IPTNT-related mechanism underlying blood flow patterns in the CNS.

Together, our findings indicate that IPTNTs regulate blood flow patterns in the retina. Here, we expand the existing neurovascular model to a system where blood flow is modulated at the capillary level to coordinate the supply of oxygen and energy substrates between adjacent neuronal areas with functional opponency. Accordingly, we consider that neurovascular coupling depends not only on the increase of blood flow but also on its decrease to achieve homogenization of blood flow. Future studies should investigate whether IPTNTs in the human retina^24^ and the brain^23^ also connect vessels with faster and slower blood flow to regions with functional opponency. Areas of the visual cortex or superior colliculus, such as the ocular dominance columns^107^ or orientation columns, are ideal candidates for study^106^. Assessment of blood flow patterns with different stimuli may lead to diagnostic and therapeutic approaches for neurodegenerative diseases.

## Methods

### Experimental animals

All animal procedures were approved by the ethical guidelines set by the animal ethics committees at St. Vincent’s Hospital (protocol #: 003.22; VIC, Australia) and The Florey Institute of Neuroscience and Mental Health (protocol #: 22-001-CERA; VIC, Australia), complying with the Prevention of Cruelty to Animals Act and the NHMRC Australian code. Experiments included adult female and male BALB/c mice and mice expressing the red fluorescent protein under control of the NG2 (*Cspg4*) promoter (NG2-DsRed) (008241, Jackson Laboratory, Bar Harbor, ME) of 2–5 months of age and 20–35 g. Animals were housed in 12 h light/12 h dark cyclic light conditions, with an average in-cage illumination level of 10–20 lux and fed ad libitum. All procedures were performed under general anesthesia with a mix of 100 mg/kg ketamine and 10 mg/kg xylazine or with 2–5% isoflurane + 98–95% O_2_ at 2–3 L/min. Isoflurane is a potent vasodilator, which may prevent further vasodilation after light stimulation^90^, and an inhibitor of the RGC function^91^. On the other hand, ketamine induces minimal cardio-respiratory depression, it does not inhibit RGC function, and only induces L-type calcium channel blockage at high-concentration doses, which are not used in this study^90,91,93–95^. Accordingly, all hemodynamic experiments were performed with ketamine/xylazine. Ischemia-reperfusion surgeries were performed with ketamine/xylazine, except for one-week reperfusion groups that were performed with isoflurane to allow a faster recovery after surgery. Intravitreal injections of AAV were performed with isoflurane.

### Intravitreal injections

Intravitreal injections were performed using a custom-made glass micropipette inserted through the superior sclera to deliver 2 µl of volume into the vitreous. Care was taken to avoid intraocular structures or retinal detachment. We made intravitreal injections of TRITC-lectin (5 µg/ml in phosphate-buffered saline, PBS; Sigma, St Louis, MO) for visualization of the vessel and IPTNT basement membrane^23,25^, intravitreal injection of AAV serotype 9 carrying GCaMP6f under the control of the synapsin promoter (AAV-Syn-GCaMP6f; 1 × 1013 particles per millilitre; Addgene, Watertown, MA) to measure RGC function in vivo, the matrix metalloproteinase-9 (MMP9) inhibitor I, C_27_H_33_N_3_O_5_S (20 µM, Merck-Millipore, Burlington, MA)^53^, or the DQ-Gelatin (D12054, Life Technologies, Carlsbad, CA) diluted to a concentration of 1 mg/ml in sterile PBS, a FITC-gelatin probe used to quantify MMP9 activation^53^.

### TPLSM live retinal imaging

TPLSM retinal imaging in living mice was performed as previously^23,25^. Anesthetised mice with a mix of ketamine and xylazine were placed on a custom-made mouse holder and setup that allowed simultaneous live retinal imaging and light stimulation. Mouse body temperature was monitored during imaging and set to 37 °C (Life Imagine Services, The Cube, Switzerland). The superior and inferior eyelids were opened, and a 6-0 suture was placed in the superior conjunctiva to gently rotate the eye. The conjunctiva over the imaging area was gently removed, exposing the underlying sclera. A 5-mm-diameter coverslip (Harvard Apparatus, Holliston, MA) was placed over the sclera, creating a planar surface necessary for retinal imaging (field of view ~400 × 400 µm) using a multiphoton microscope (Olympus FVMPERS, Tokyo, Japan; Zeiss LSM 780, Oberkochen, Germany) with a water objective (25×, NA = 1.05; 20×, NA = 1.00) controlled by FV30S-SW software (Olympus) or Zen software (Zeiss). We confirmed that the coverslip did not interfere with the blood flow by measuring the flux in the same vessels with minimal contact area (intraocular pressure without coverslip: 11 mmHg; intraocular pressure with minimal contact: 11 mmHg) and with our standard contact area (intraocular pressure with standard contact = 12 mmHg) (Supplementary Fig. 1D). For the excitation of fluorescein, a mode-locked Ti:sapphire (InSight; Spectra-Physics, Milpitas, CA; Chameleon Ultra, Coherent, Saxonburg, PA) was set to 920 nm; for the excitation of TRITC-lectin, the excitation wavelength was set to 1020 nm, with a mean laser power of 15–50 mW. Intraperitoneal (IP) injection of diluted fluorescein (5% in 100 µl, Retinofluor; Phebra, Australia) was performed to allow visualization of retinal blood flow. Line scans (~ 800 Hz) through individual retinal capillaries allow haemodynamic parameters to be measured (i.e., blood cell flux, velocity, and diameter). A line scan perpendicular to the vessel lumen produces distance(x; 0.331 µm per pixel)/time(y; ~1.2 ms per pixel) recordings with shadows against the fluorescent plasma that indicate individual RBCs over time as blood cells do not take up fluorescein, allowing flux (RBC/s) assessment (Supplementary Fig. 1A). A longitudinal line scan through the central part of the capillary produces another distance(x)/time(y) recordings showing the movement of single blood cells through the portion of the capillary scanned by the linear probe (Supplementary Fig. 1B). The angle of the oblique shadow with respect to the horizontal axis allowed RBC velocity to be calculated as speed = ∆x/∆t (Fig. 1D)^32^. IPTNT-connected vessels were identified with intravitreal-injected TRITC-lectin. Non-IPTNT-connected vessels were defined as lacking any IPTNT staining with lectin and classified as capillaries immediately adjacent and with a distance = ~IPTNT-length average (i.e., ~50 µm). For consistency with IPTNT experiments, no distinction was made from capillaries within or outside the same capillary tree. Each capillary was recorded over ~10–130 s. To assess light-evoked changes in blood flow patterns, a flash stimulus (10^2^ cd/m^2^, 1 s) was generated with a Powerlab unit (ADInstruments, Colorado Springs, CO) connected to a white light-emitting diode, located 5 mm away from the cornea. Recordings with large amplitude motion were discarded.

### Analysis of blood cell flux, velocity, diameter, and capillary dynamics

Immediately prior to TPLSM imaging, an IP injection of fluorescein was performed to label vessels. To visualize IPTNTs, we intravitreally injected TRITC-lectin 30 min before imaging. Line-scan recordings were analyzed in ImageJ/Fiji software (National Institute of Health: NIH, Bethesda, MD) with a stereological analysis, which consisted of 100 disectors of equal size placed along the whole recording with a systematic uniform random sampling. For blood cell flux, blood cells were manually identified per disector and computed as RBC per second (RBC/s) over time and as % of change related to values prior to the stimulus using a custom R-script (https://www.r-project.org). For blood cell velocity, oblique shadows corresponding to blood cells were measured using a line tool to assess the angle with the horizontal axis in one or two blood cells per disector (Fig. 1D), which allowed computation of velocity (i.e., v = *Δx/Δt*) in mm per second (mm/s) over time and as % of change related to values before the stimulus using an R-script. For capillary diameter, we confirmed that our system can resolve light-evoked diameter changes by quantifying the maximum/minimum responses for each vessel and validating that the change is larger than the spatial resolution of our system (1 pixel = 0.3 μm). The diameter was measured once per disector and computed as diameter over time and as % of change related to values before the stimulus using an R-script. For experiments without light stimulation, blood cell flux and velocity were calculated as the average of all disectors over time. For experiments with light stimuli, each IPTNT-connected vessel was classified as either faster or slower depending on its flux or velocity value prior to light stimulation. Blood cell flux, velocity, and diameter values before the stimulus were calculated as the average of the 20 disectors prior to light presentation. Accordingly, maximum (for slower capillaries) and minimum (for faster capillaries) responses after light were calculated as the maximum/minimum value of the average of 20 consecutive disectors after light stimulation. The difference between vessels or intercapillary difference denoted the degree of heterogeneity/homogeneity. The change of intercapillary difference after light stimulation denoted the capacity of homogenization (i.e., light-evoked homogeneity = intercapillary difference before light—intercapillary difference after light). To confirm that blood flow changes did not result from random vascular variations, control experiments without light were performed precisely in the same way, comparing before and after a ~ 10-s timepoint following the beginning of the recording. Polynomial parametric functions were fitted to individual vessel responses to obtain the time to peak vessel dilation/contraction.

### Retinal and brain preparation and immuno-histochemistry protocols

Mice were placed in deep anaesthesia with a mix of ketamine and xylazine, and transcardiac perfusion with 20 ml of PBS and then 20 ml of 4% paraformaldehyde (PFA) was performed to fix the retinal tissue. The globe, including the optic nerve, was gently removed using a scalpel and placed in 4% PFA for a further hour and then transferred to PBS to dissect whole flat retinas. Dissected retinas were permeabilised by washing in 0.5% PBS in Triton ×-100 (PBST) for 10 min and repeated three times. The retinas were placed in a −80 °C freezer for 15 min. After thawing, retinas were washed a further two times in 0.5% PBST. Retinas were incubated in blocking solution (2% normal donkey serum in 2% PBST) at 4 °C with the following primary antibodies: laminin-2 (Lama2, L0663, 5 days, 9.6 μg/ml, Merck-Millipore), non-phosphorylated anti-neurofilament H (SMI-32, 801701, 2 days, 4 μg/ml, Biolegend, San Diego, CA), conjugated-488 Brain-Specific Homeobox/POU Domain Protein 3 A (Brn3a, SC8429AF488, 2 days, 1 μg/ml, Santa Cruz Biotechnology, Dallas, TX), neuron glial 2 (NG2, AB5320, 5 days, 5 μg/ml, Merck-Millipore), Forkhead box protein P1 (Foxp1, ab16645, 3 days, 2 μg/ml, Abcam), Forkhead box protein P2 (Foxp2, ab1307, 3 days, 1 μg/ml, Abcam), and alpha smooth muscle actin (αSMA, A5228, 5 days, 10 μg/ml, Sigma). Retinas were subsequently washed (3×, 0.5% PBST, 10 min/each) and incubated overnight in 2% PBST with the following Fab fragments of secondary antibodies essential for proper penetration into the tissue (Jackson ImmunoResearch, West Grove, PA): for Lama2, donkey anti-rat 647 nm (6 μg/ml, 712-607-003), for SMI-32, donkey anti-mouse Cy3 (3 μg/ml, 715-167-003), for NG2, donkey anti-rabbit 488 nm (6.4 μg/ml, 711-547-003), for Foxp1, donkey anti-rabbit Cy3 (6 μg/ml, 711-167-003), for Foxp2, donkey anti-goat 488 nm (7.2 μg/ml, 705-547-003), and for αSMA, donkey anti-mouse 488 nm (7.2 μg/ml, 715-547-003). Retinas were then washed (3×, PBS, 10 min/each), applied the nuclear marker Hoechst 33342 (10 μg/ml, Life Technologies Corporation, Eugene, OR), and mounted on a glass slide with a #1.5 coverslip using SlowFade Diamond Anti-Fade mounting medium (Life Technologies Corporation). Importantly, a thin spacer was placed adjacent to the retina to preserve IPTNT integrity. Brain tissue was dissected by manually cutting a thin transverse section of the superior colliculus and visual cortex using an ophthalmic knife. Brain tissue was permeabilised and freeze/thawed in the same manner as the retina. Brain tissue was incubated in blocking solution at 4 °C with Lama2 (9.6 μg/ml, 5 days, Merck-Millipore) and platelet-derived growth factor receptor beta (PDGFRb, AF1042-SP, 0.8 μg/ml, 5 days, R&D Systems, Minneapolis, MN). Tissue was subsequently washed (3×, PBS, 10 min/each), applied the nuclear marker Hoechst 33342 (10 μg/ml, Life Technologies Corporation), and mounted on a glass slide with a #1.5 coverslip using SlowFade Diamond Anti-Fade mounting medium (Life Technologies Corporation).

### Detection of mRNA expression in flat-mounted retinas

Mouse eyes were enucleated and immersion-fixed for 1 h in 4% PFA. Retinas were processed using an RNAscope wholemount approach adapted from Kersigo et al.^42^, using reagents from the RNAscope™ Multiplex Fluorescent Reagent Kit v2 (ACD, Newark, CA). Following dissection, retinas were washed 3 times in 0.1% Tween 20 in PBS (PBSTween), dehydrated with a graded methanol series (50, 75, and 100% methanol in PBSTween) and subsequently rehydrated in 75% and 50% methanol in PBSTween. Retinas were washed 3 times in PBSTween and then incubated with Protease III for 7 min at room temperature. Retinas were then washed 3 times in PBSTween and incubated overnight at 40 °C in probe solution containing Mm-Calb1 (ADV428431), Mm-Col24a1-C2 (ADV538511C2), and Mm-RBPMS-C3 (ADV527231C3) diluted at a 40:1:1 ratio, with shaking (70 rpm). Retinas were then washed 3 times in RNAscope-wash buffer and fixed in 4% PFA for 10 min. To amplify the signal, retinas were washed 3 times in RNAscope-wash buffer and incubated at 40 °C with AMP1 for 35 min, AMP2 for 20 min, and AMP3 for 35 min; washing 3 times with RNAscope-wash buffer between each incubation. To fluorescently label the probes, retinas were incubated at 40 °C with horseradish peroxidase (HRP)-C1 for 35 min, tyramide signal amplification (TSA) vivid fluorophore (1:750 in multiplex TSA buffer) for 1 h, and HRP blocker for 35 min; washing 3 times with RNAscope buffer between each incubation. The same was repeated for HRP-C2 and HRP-C3. TSA fluorophores 520, 570, and 650 were used to detect HRP-C1, HRP-C2, and HRP-C3, respectively. After the RNAscope protocol was completed, retinas were incubated in blocking solution 2% normal donkey serum in 2% PBST at 4 °C with rat anti-laminin-2 (9.6 μg/ml, Merck-Millipore) for 2 nights. Retinas were washed 3 times in PBST, then incubated in donkey anti-rat 488 nm. Retinas were incubated in Hoechst 33342 (10 μg/ml, Life Technologies Corporation) and mounted onto a glass slide with a #1.5 coverslip using SlowFade Diamond Anti-Fade mounting medium (Life Technologies Corporation).

### In vivo calcium analysis of retinal ganglion cells

To evaluate calcium responses in RGCs, we intravitreally injected 2 µl of AAV-Syn-GCaMP6f (Addgene) at least 3 weeks prior to TPLSM imaging to induce the expression of the genetically encoded calcium indicator, GCaMP6f, in RGCs^25^. Lectin diluted in PBS was injected intravitreally to identify IPTNTs and vessels. Calcium changes for RGCs expressing GCaMP6f nearby vessels connected by IPTNTs were longitudinally recorded by TPLSM (excitation: 920 nm). Calcium signals were calculated as Δ*F/F* = (*F-F*_*0*_)*100/*F*_*0*_, where *F*_*0*_ is the fluorescence baseline and *F* is the fluorescence at time *t* calculated based on signal extraction methods^108^. Frame data (512 × 512; ~14 Hz) was collected. Recordings consisted of at least 5 s of pre-stimulus condition to establish baseline calcium spontaneity. Responses were recorded for ~10 s afterwards. As photomultipliers (PMT) of the TPLSM saturate during light stimuli, our system was designed to automatically shut down the PMT when the light stimuli were presented, and an ImageJ/Fiji software (NIH) script subtracted any blank frames. Thus, a one- or three-second light stimulus was presented to allow the characterization of both ON and OFF RGCs (transient and sustained), as well as ON-OFF subtype as previously^35^. Accordingly, we presented a 1-s stimulus to register the RGC response. Then, to determine whether RGC responses were aligned with the light onset or with the light offset, we presented a 3-s stimulus and identified if the response only occurred after the stimulus (i.e., aligned with the light offset). Recordings were maintained in focus manually during the whole acquisition. X-Y movements were corrected with the “*TurboReg*” plug-in of ImageJ/Fiji software (NIH). Importantly, blood flow in each IPTNT-connected capillary was assessed as stated above. In this way, both blood flow dynamics and RGC responses could be observed in the same mouse, and we were able to study the association between ON/OFF areas and slower-faster capillaries.

### Polarity index analysis

#### In vivo

To assess the contribution of each RGC to the IPTNT-dependent pattern formation, we measured the distance from each RGC to the nearest IPTNT (Supplementary Fig. 14A–C). For this, we placed a 50 µm-diameter area centred at the proximal capillary precisely on the pericyte soma from where the IPTNT was formed, and another circular area centred at the distal capillary over the IPTNT-endfoot. RGCs falling within these circular regions were numbered for analysis and classified as ON, OFF, or ON-OFF RGCs based on calcium changes. The diffusion of a substance through the extracellular space is described by a Gaussian curve^109,110^ with a distance-dependent factor defined by:

1 $${e}^{-\left(\frac{{r}^{2}}{{4D}{*}}\right)}$$

where r is the distance from the source (i.e., RGC) and D* is the effective diffusion coefficient through the extracellular space of a substance (i.e., neurotransmitter). Accordingly, we measured the distance from each RGC soma to the centre of the circular area and subtracted 5 μm (to exclude soma size). As a major neurotransmitter in the retina^111^, we used the glutamate D* value (i.e., 450 µm^2^/s)^112,113^. Thus, each RGC was given a value following the equation:

2 $${RGC}{\prime} s\,{contribution}\,{to}\,{area}\,{polarity}\,{index}={e}^{-\left(\frac{{({{{\rm{distance}}}}\; ({{{\rm{\mu }}}}m)-5)}^{2}}{4*450}\right)}$$

ON-RGC related values were represented by positive values and grouped, and OFF-RGC related values by negative ones and grouped. ON-OFF-RGCs contributed equally to both groups. RGCs closer to a capillary provide a greater contribution to ON/OFF indexes. The contribution value for all RGCs within an area was summed to obtain a polarity index for each region. A positive and negative score represented an ON- or OFF-preferred area response, respectively. The difference between the values of both IPTNT-connected areas provided us with a polarity ON/OFF index associated with a pair of capillaries.

#### Ex vivo

Unbiased stereology was performed in fixed flat-mounted retinas with specific markers for subtypes of ON and OFF RGCs. Thus, we identified α-ON (i.e., Brn3a−/SMI-32+) and α-OFF (i.e., Brn3a+/SMI-32+) RGCs^35,38^, F-ON (i.e., Foxp1+/Foxp2+) and F-OFF (i.e., Foxp1−/Foxp2+) RGCs^40^, and ooDSGCs responding to opposite directions (i.e., dorsal: RBPMS^+^/Col25a1^+^/Calb1^−^; ventral: RBPMS^+^/Col25a1^+^/Calb1^+^)^43^. IPTNTs were selected using systematic uniform random sampling along the whole retina. We followed the same method as for in vivo analysis, but this time, RGCs falling within 50 µm-diameter regions were classified as ON or OFF RGCs depending on their markers. ON/OFF scores for each area and the polarity ON/OFF index associated with each pair of capillaries were calculated similarly to the in vivo analysis. Similarly, a positive and negative score represented a ventral-direction- or dorsal-direction-preferred area response, respectively.

### IPTNT location analysis, quantification of IPTNT and vessel density, IPTNT length, and capillary order

A Leica Stellaris 5 Inverted confocal microscope (CLSM) controlled by LAS X software (Leica, Wetzlar, Germany) was used to image the retinal, superior colliculus, and visual cortex preparations using a 20× air objective and a resolution of 1024 × 1024. For the retina, we used an unbiased stereological approach based on systematic uniform random sampling from 3D-disectors (25 ± 1.0 slices per stack; 2 µm between slices) across the entire retina (97 ± 3.2 3D-disectors with a field of view of 185 × 185 μm). The entire superior colliculus and visual cortex tissue was imaged throughout a 40-µm depth (20 slices). Using ImageJ/Fiji software (NIH), we identified the arteriole-capillary transition zone (TZ) as the vascular branches where robust αSMA expression progressed from high to low levels, then we classified IPTNT-connected pericytes as in if they were located before/within transition zone or as out if they were beyond the transition zone, as well as αSMA+ or αSMA−. We quantified the capillary segments and the intact and ruptured Lama2-positive IPTNTs, calculating density values. Disectors with ruptured tissue from the dissection process were not included in the analysis. For length measurement, IPTNTs were randomly selected using systematic uniform random sampling and measured using ImageJ/Fiji software (NIH). For comparison purposes with previous reports in the brain (where the 1st-order vessel is identified as the first branch division from a penetrating arteriole)^32,78^, the first-order vessel was defined as the first branch division from a whole radiating arteriole (i.e., zero-order vessel)^46,50^. Then, we assigned increasing orders for each branch until reaching IPTNT-connected capillaries and established whether they emanated from the same or different origin branches. In vivo vessel order was obtained using TPLSM to determine the direction of the blood flow in major radiating retinal vessels, which allowed the objective differentiation between arterioles (flow travelling from optic nerve head to the retinal periphery: centrifugal flow direction) and venules (flow travelling from retinal periphery to the optic nerve head: centripetal flow direction).

### In vivo laser-induced IPTNT ablation

Prior to laser ablation, light-evoked flux and velocity were measured in IPTNT-connected vessels. In the same vessels, disruption of their associated IPTNTs was performed using calibrated laser parameters (1020 nm; 100% power, 5 s) targeting a small area (1 μm^2^) that covered the IPTNT extent to cause rupture, which could be visualised in situ. Then, light-evoked flux and velocity were re-evaluated, and a comparison of before and after laser-induced ablation of IPTNT-connected vessels was performed.

### TUNEL assay

Following in vivo laser-induced IPTNT ablation, the terminal deoxynucleotidyl transferase dUTP Nick-End Labeling (TUNEL) assay was performed in ex vivo flat-mounted retinas to identify potential apoptotic cells induced by laser. The precise location of every IPTNT was determined in vivo by capturing large field-of-view scans. A suture was placed in the superior pole of the eyes to keep their orientation. Then, the animal was culled, and the eyes were fixed in 4% paraformaldehyde for 1 h, and then transferred to PBS. The retinas were dissected with the superior retinal petal being larger than the other ones to maintain orientation. The DeadEnd^TM^ Fluorometric TUNEL System (Promega, Madison, WI) was used to assay apoptosis following laser-induced IPTNT ablation. The retinas were re-fixed following dissection (4% PFA, 15 min), washed twice (PBS, 5 min) and incubated in proteinase K (100 µl, 20 µg/ml, 12 min). The retinas were then washed (PBS, 5 min), re-fixed (4% PFA, 15 min), re-washed (PBS, 5 min), placed on slides, and incubated in equilibration buffer (100 µl, 10 min). Incubation buffer containing equilibration buffer (45 µl), Nucleotide Mix (5 µl) and rTdT Enzyme (1 µl) was added per retina and covered with a plastic coverslip, and allowed to incubate at 37 °C for 1 h. The coverslips were removed, and the reaction was stopped using 2 × SSC (15 min). Lastly, the retinas were washed three times (PBS, 5 min), orientated, and mounted using SlowFade Diamond Anti-Fade mounting medium (Life Technologies Corporation). Using the in vivo field of view images, the in vivo laser-damaged IPTNTs were identified in the ex vivo retinas with a CLSM (Leica Stellaris 5, Leica, Wetzlar, Germany), and then z-stacks were acquired (red for TRITC-lectin and green for fluorescein-12-dUTP).

### Transient retinal ischemia-reperfusion

Transient ischemia was generated through careful dissection of the living eye to access the optic nerve and its dural sheath, which was longitudinally cut to expose the optic nerve. A fine (10-0) nylon suture was gently passed between the optic nerve and the sheath—where the ophthalmic vessels travel through—and tied around the sheath to interrupt retinal and choroidal blood supply without damaging the optic nerve. Ligature was tied for 60 min (i.e., ischemia) and then released, and blood was reperfused for one week (i.e., reperfusion).

### Quantification of MMP9 activation

A FITC-gelatin probe (DQ-Gelatin, D12054, Life Technologies) diluted to a concentration of 1 mg/ml in sterile PBS was co-injected intravitreally with TRITC-lectin (5 µg/ml, Sigma). Thirty minutes before ischemia induction, MMP9 inhibitor (20 µM, Merck-Millipore) or PBS was intravitreally injected. After one-hour ischemia or sham surgeries, animals were culled, eyes were collected and drop-fixed in 4% paraformaldehyde (PFA) overnight. Whole retinas were dissected and flat-mounted as described earlier and immediately imaged via CLSM (Leica). The superficial plexus was imaged using unbiased stereology, at 20× magnification, generating 3D-disectors (22 ± 0.5 slices per stack; 0.68 µm between slices) under identical laser settings for all the cohorts. FITC intensity dependent on MMP9 activation was quantified using Fiji software (NIH) following previous methods^53^. Accordingly, the stack images were sum-projected, and the FITC intensity was measured using a 2-µm diameter region of interest at the proximal, mid, and distal sites of each IPTNT. Then, the FITC intensity per slice was calculated by dividing the intensity values by the number of slices per stack. The background FITC intensity per slice was quantified by averaging the intensity of the whole imaged regions divided by the number of slices of each stack. Positive IPTNTs for FITC-gelatin were defined as IPTNTs with 35% greater fluorescence intensity than the background.

### Statistical analyses

Data was analyzed using unbiased stereological methods described above with automatic software routines. This method avoids biased analyses, ensuring random data collection from large samples and line-scan recordings. Data analysis for blood cell flow and velocity was performed without knowing the faster or slower vessel, characterization of RGCs for in vivo polarity ON/OFF index was performed after calculating calcium signals with automatic routines in ImageJ/Fiji software (NIH) and R, IPTNTs and capillaries used in the polarity ON/OFF index analysis were selected without knowing the type of cells surrounding vessels, and analysis of MMP9 activation was performed with automatic methods in ImageJ/Fiji software (NIH). The number of animals used in each experiment, as well as the number of IPTNTs, capillaries, cells, or areas quantified/analyzed is indicated in the figure legends. Statistical analysis was performed with Prism 10 (GraphPad, Boston, MA). All values are provided as the mean ± standard error of the mean (S.E.M.), and individual values are presented in each graph. We first evaluated all cohorts with normality (Shapiro–Wilk test) and variance (*F*-test) tests. A linear mixed-effects model was used to compare dependent cohorts. A paired *t*-test or Wilcoxon matched-pairs signed-rank test, where appropriate, was used to compare the same cohorts measured under different time/conditions (e.g., before vs. after light). An unpaired two-tailed Student’s *t*-test or Mann–Whitney *U* test, where appropriate, was used to compare two independent cohorts. We used Analysis of Variance (ANOVA) for multiple comparisons, followed by Tukey’s or Kruskal–Wallis test, where appropriate, to compare three or more independent groups. We used Spearman’s rank correlation coefficient to test the correlation between velocity and flux. A *P*-value ≤ 0.05 will be considered significant. Power analyses coupled with experience with previous recordings/experiments estimated the sample size of mice/group to achieve 80% power to detect statistical differences between groups (*α* = 0.05) when the study was designed. All trendlines of blood cell flux and velocity graphs were fit with the same order between the experimental and control groups.

### Reporting summary

Further information on research design is available in the Nature Portfolio Reporting Summary linked to this article.

## Supplementary information

Supplementary Information

Description of Additional Supplementary Files

Supplementary Movie 1

Supplementary Movie 2

Supplementary Movie 3

Supplementary Movie 4

Reporting Summary

Transparent Peer Review file

## Source data

Source Data

## Data Availability

Source data are provided with this paper. The data generated in this study are provided in the Supplementary Information/Source Data file. Source data are provided with this paper.

## Code availability

The code used for data analysis is included in the supplementary information files and via a link to Code Ocean: 10.24433/CO.0048713.v1.
