# Supplementary material for: Blood flow patterns in mice are regulated by interpericyte tunneling nanotubes connecting functionally-opposite neuronal areas
Source: Nat Commun. 2026 Apr 13;17:5141. doi: 10.1038/s41467-026-71804-2 (PMC13250102; doi:10.1038/s41467-026-71804-2)
Supplement: Supplementary file 1 — Supplementary Information [file 41467_2026_71804_MOESM1_ESM.pdf]

Supplementary Information – Figures

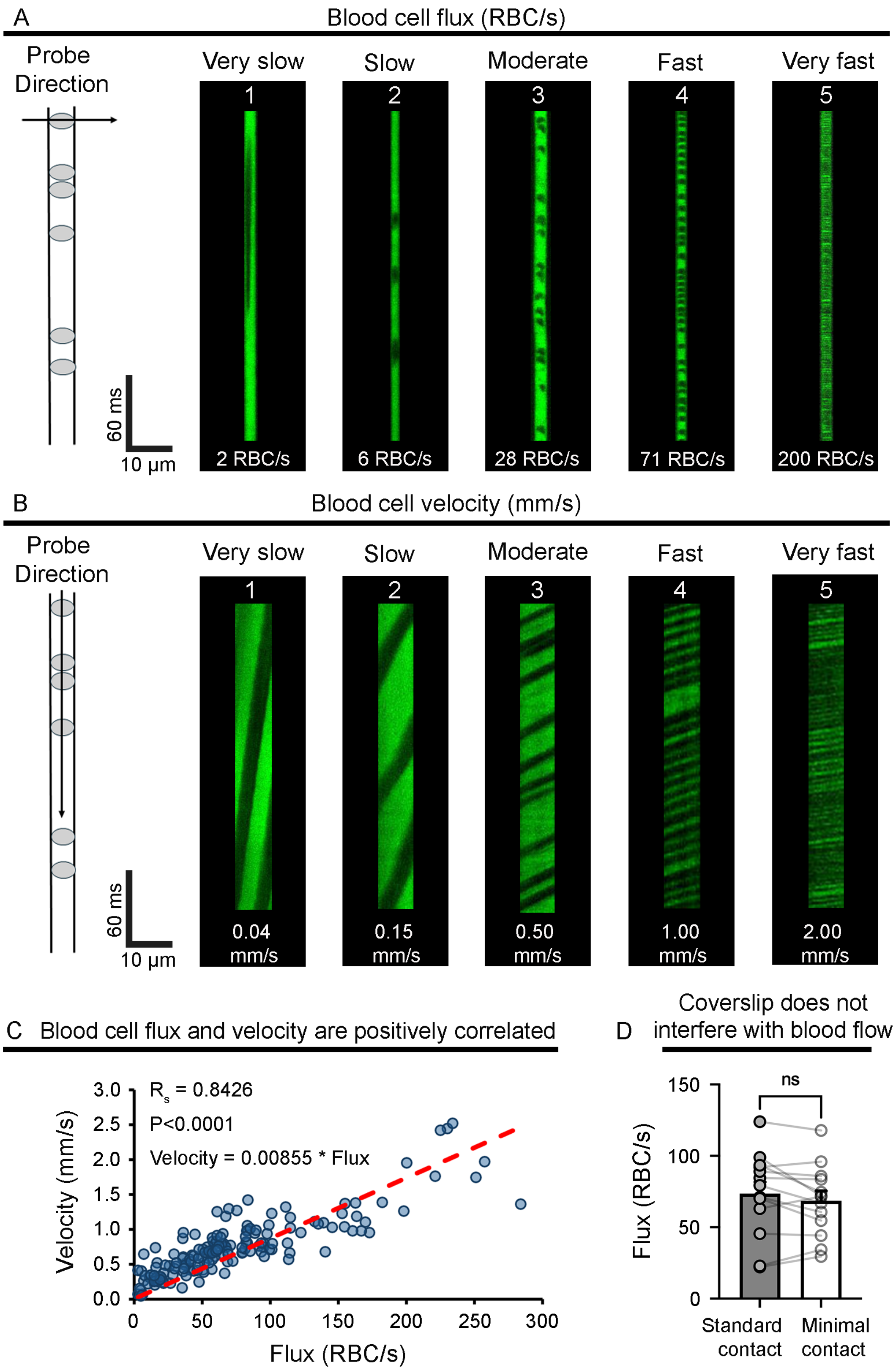

Supplementary Figure 1. Blood cell flux and velocity tend to increase/decrease together.

(A-B) *In vivo* two-photon line-scan recordings of capillaries labeled with an IP injection of fluorescein (green). We identified blood cells as shadows against the fluorescent plasma, as they do not take up fluorescein. Recordings to quantify the number of blood cells over time or blood cell flux were made with a line-scan probe (~800 Hz) perpendicular to the recorded vessel (A). Recordings to quantify the velocity of the blood cells were made with a line-scan probe parallel to the recorded vessel (B). Recordings of blood cell flux (A) and velocity (B) of five capillaries (1-5) with very slow, slow, moderate, fast, and very fast hemodynamics showing an increase of blood cell flux and velocity (A-B). (C) Graph showing that blood cell flux and velocity are positively correlated (n=127 capillaries, N=13 mice, Spearman's rank correlation coefficient R_s=0.8426, P<0.0001). (D) *In vivo* quantification of blood cell flux in the same capillaries with minimal and standard coverslip contact (n=13 capillaries, N=2 mice, two-tailed paired Student's *t*-test, ns: not significant). Intraocular pressure without coverslip: 11 mmHg; intraocular pressure with minimal contact: 11 mmHg, intraocular pressure with standard contact = 12 mmHg. Source data are provided as a Source Data file.

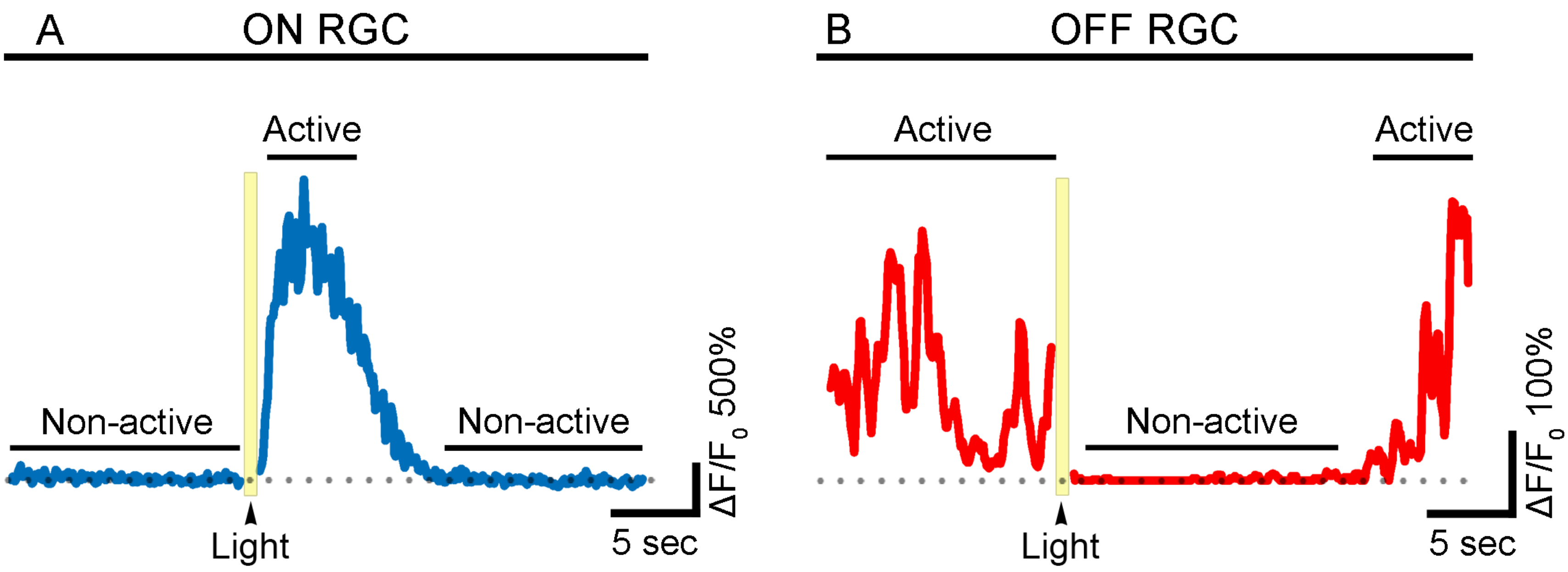

Supplementary Figure 2. RGC calcium responses. (A-B) An ON RGC (A) and an OFF RGC example (B) classified as previously [1]. Light evokes an increase (A) or decrease (B) of the calcium signal in the ON and OFF RGC, respectively.

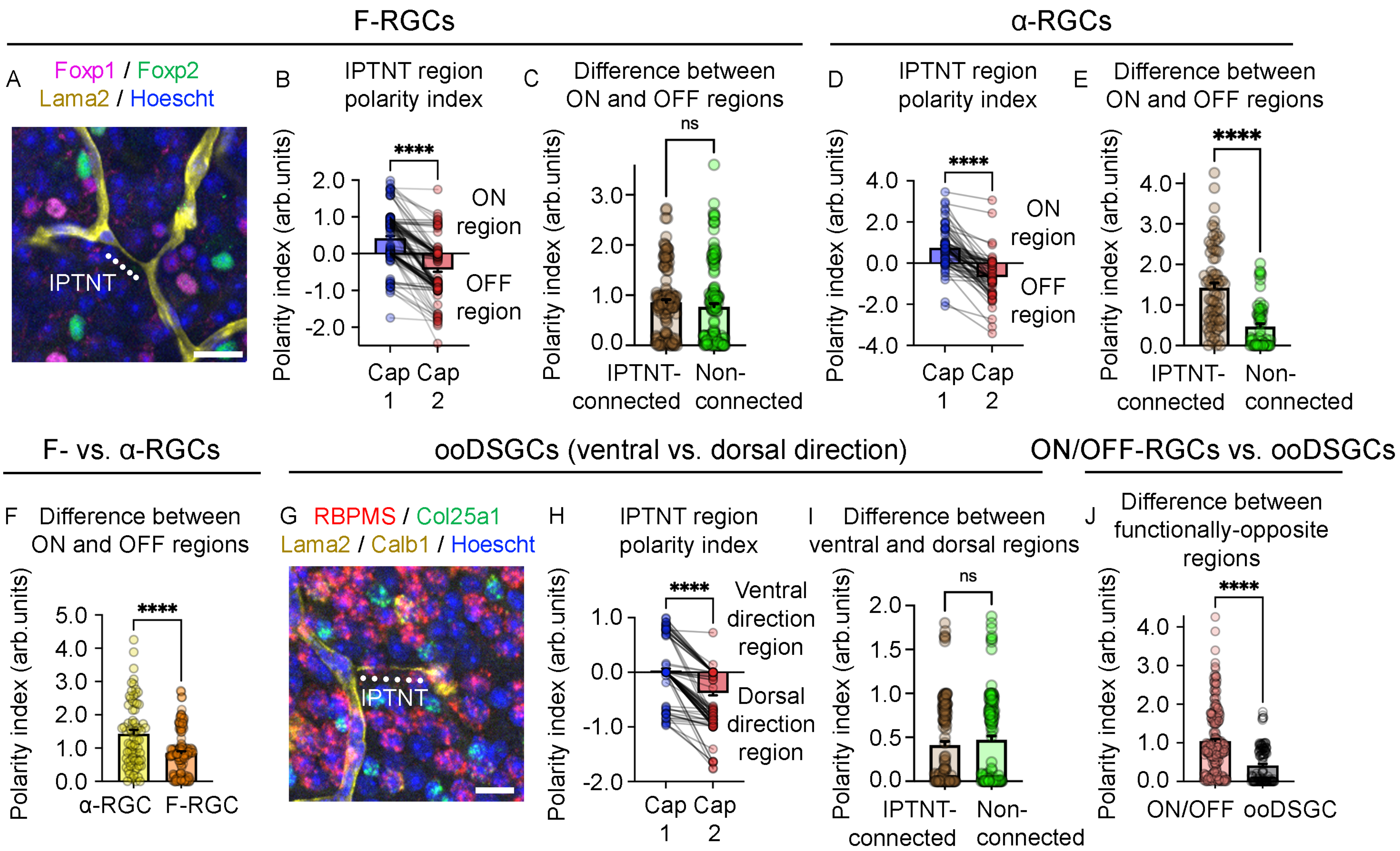
F-RGCs
α-RGCs
A Foxp1 / Foxp2 Lama2 / Hoescht
IPTNT
B IPTNT region polarity index
****
ON region
OFF region
Polarity index (arb.units)
2.0
1.0
0.0
-1.0
-2.0
Cap 1
Cap 2
C Difference between ON and OFF regions
ns
3.0
2.0
1.0
0.0
IPTNT-connected
Non-connected
D IPTNT region polarity index
4.0
2.0
0.0
-2.0
-4.0
E Difference between ON and OFF regions
F- vs. α-RGCs
ooDSGCs (ventral vs. dorsal direction)
ON/OFF-RGCs vs. ooDSGCs
F Difference between ON and OFF regions
5.0
4.0
α-RGC
F-RGC
G RBPMS / Col25a1 Lama2 / Calb1 / Hoescht
H IPTNT region polarity index
Ventral direction region
Dorsal direction region
I Difference between ventral and dorsal regions
1.5
0.5
J Difference between functionally-opposite regions
ON/OFF
ooDSGC

<u>Supplementary Figure 3. The magnitude of the opposite-functional areas connected by IPTNTs is dependent on the RGC subtypes.</u> (A) *Ex vivo* flat retina labeled with antibodies against Foxp1 (magenta), Foxp2 (green), and Lama2 (yellow) and the nuclear marker Hoechst (blue) (A) used to identify IPTNTs (dotted line) and F-ON (i.e., Foxp1+/Foxp2+) and F-OFF (i.e., Foxp1-/Foxp2+) RGCs as previously[2]. (B-C) *Ex vivo* polarity-index graph shows IPTNTs connecting opposite-functional neuronal areas along the whole retina for F-RGCs (n=306 areas, n=459 cells, N=3 mice, linear mixed-effect model analysis, ****p<0.0001) (B), which was similar to the index between areas surrounding non-connected vessels (n=306 areas, n=459 cells, N=3 mice in IPTNT-connected group; n=312 areas, n=459 cells, N=3 mice in non-connected group, linear mixed-effect model analysis, ns: not significant) (C). (D-E) *Ex vivo* polarity-index graph shows IPTNTs connecting opposite-functional neuronal areas along the whole retina for α-ON and α-OFF RGC analyzed (n=146 areas, n=300 cells, N=4 mice, linear mixed-effect model analysis, ****p<0.0001) (D), which was larger than the index between areas surrounding non-connected vessels (n=146 areas, n=300 cells, N=4 mice in IPTNT-connected group; n=128 areas, n=75 cells, N=3 mice in non-connected group, linear mixed-effect model analysis, ****p<0.0001) (E). (F) Polarity-index graph showing a larger opposite-functional index between IPTNT-connected areas for α-RGCs than F-RGCs (n=146 areas, n=300 cells, N=4 mice in α-RGC group; n=306 areas/group, n=459 cells, N=3 mice in F-RGC group, two-tailed Mann-Whitney U test, ****p<0.0001). (G) *Ex vivo* flat retina labeled with RNAscope-specific probes for RBPMS (red), Col25a1 (green), Calb1 (yellow) for the detection of mRNA expression used to identify ooDSGCs responding to opposite directions (i.e., dorsal direction: RBPMS$^+$/Col25a1$^+$/Calb1-; ventral direction: RBPMS$^+$/Col25a1$^+$/Calb1$^+$)[3], an antibody against Lama2 (yellow), and the nuclear marker Hoechst (blue). (H-I) *Ex vivo* polarity-

index graph shows IPTNTs connecting opposite-functional neuronal areas along the whole retina for ooDSGCs responding to ventral and dorsal directions (n=280 areas, n=124 cells, N=3 mice, linear mixed-effect model analysis, ****p<0.0001) (H), which was similar to the index between areas surrounding non-connected vessels (n=280 areas, n=124 cells, N=3 mice in IPTNT-connected group; n=274 areas, n=130 cells, N=3 mice in non-connected group, linear mixed-effect model analysis, ****p<0.0001) (I). (J) Polarity-index graph showing a larger opposite-functional index between IPTNT-connected areas for ON/OFF RGCs than ooDSGCs (n=452 areas, n=759 cells, N=7 mice in ON/OFF group; n=280 areas, n=124 cells, N=3 mice in ooDSGC group, two-tailed Mann-Whitney U test, ****p<0.0001). Scale bar in A and G = 20 µm. Source data are provided as a Source Data file. Arb. units=arbitrary units.

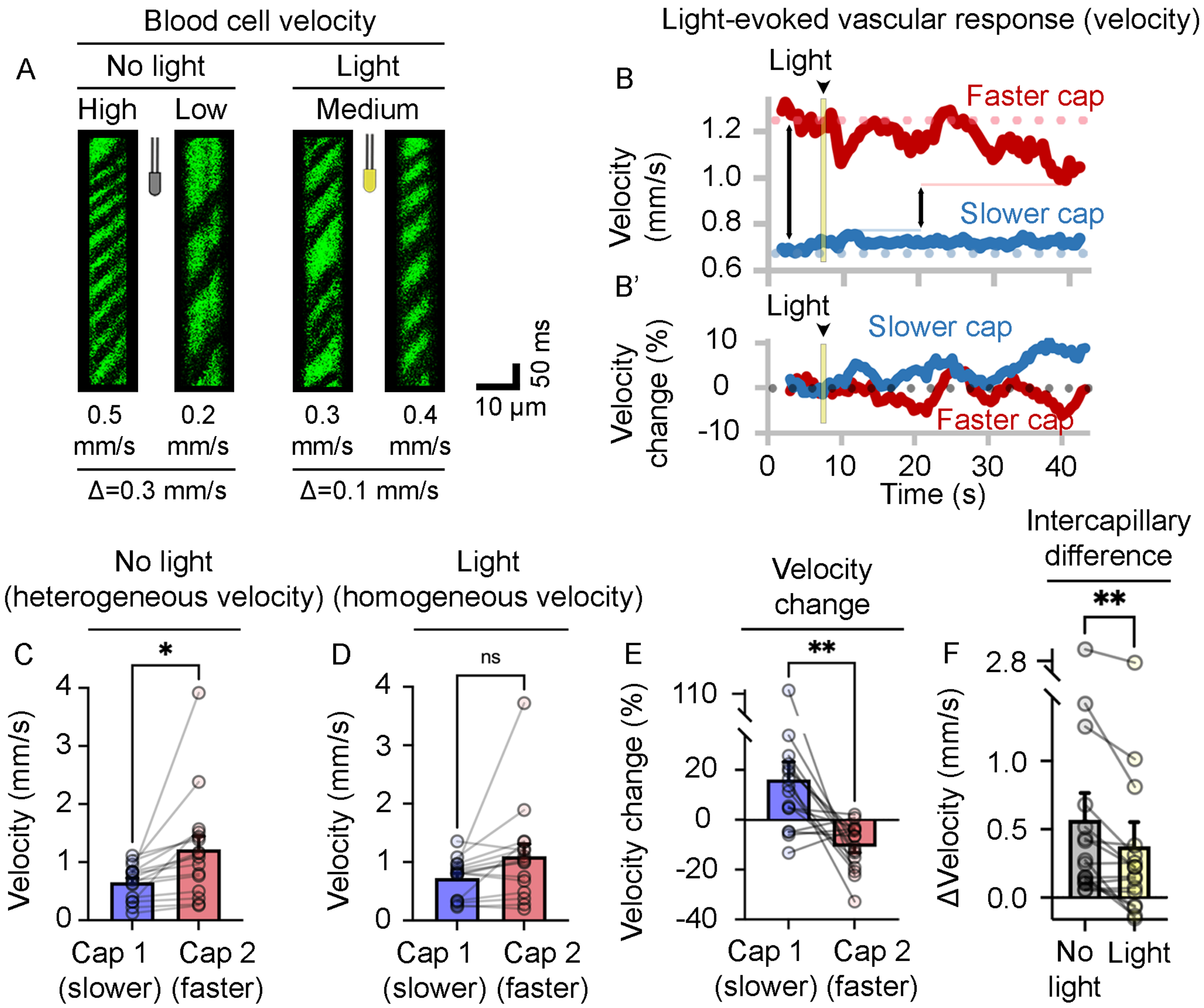

Blood cell velocity
A
No light
Light
High
Low
Medium
0.5 mm/s
0.2 mm/s
0.3 mm/s
0.4 mm/s
Δ=0.3 mm/s
Δ=0.1 mm/s
50 ms
10 µm
Light-evoked vascular response (velocity)
B
Light
Faster cap
Slower cap
Velocity (mm/s)
1.2
1.0
0.8
0.6
B'
Light
Slower cap
Faster cap
Velocity change (%)
10
0
-10
0
10
20
30
40
Time (s)
No light (heterogeneous velocity)
Light (homogeneous velocity)
C
*
Velocity (mm/s)
Cap 1 (slower)
Cap 2 (faster)
D
ns
Velocity (mm/s)
Cap 1 (slower)
Cap 2 (faster)
Velocity change
E
**
Velocity change (%)
110
20
0
-20
-40
Cap 1 (slower)
Cap 2 (faster)
Intercapillary difference
F
**
ΔVelocity (mm/s)
2.8
1.0
0.5
0.0
No light
Light

<u>Supplementary Figure 4. Light stimulation modifies blood cell velocity patterns of IPTNT-connected capillaries.</u>

(A-B) Examples of line-scan recordings before (no light) and after light stimulation (light) in IPTNT-connected vessels (A) used to calculate blood cell velocity (B) and velocity change traces over time (B') (average of 16 capillaries per trace, N=4 mice; double-headed arrows indicate intercapillary difference before and after light). (C-F) Maximum response graphs show that, prior to light, IPTNT-connected vessels have different velocities or heterogeneity (n=32 capillaries, N=4 mice, linear mixed-effect model analysis, *p=0.011) (C). After light, the velocity difference between capillaries disappears, leading to flux homogenization (n=32 capillaries, N=4 mice, linear mixed-effect model analysis, ns: not significant) (D) as slower and faster capillaries increase and decrease their velocity, respectively (E) (n=32 capillaries, N=4 mice, linear mixed-effect model analysis, **p=0.006). Accordingly, intercapillary velocity difference is significantly reduced after stimulation compared to values before light (n=32 capillaries, N=4 mice, two-tailed Wilcoxon matched-pairs signed-rank test, **p<0.002) (F). Source data are provided as a Source Data file.

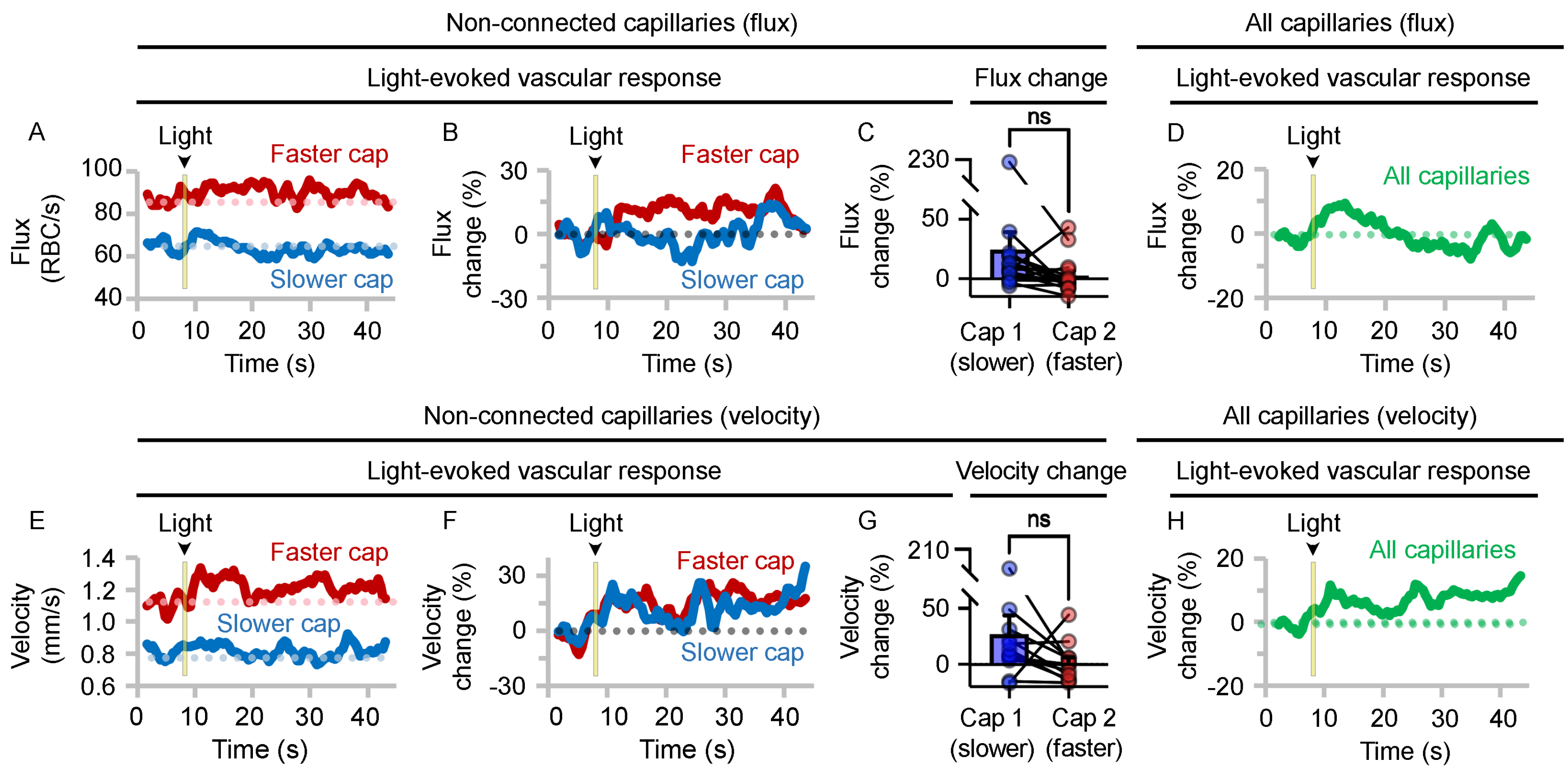

Non-connected capillaries (flux)
All capillaries (flux)
Light-evoked vascular response
Flux change
Light-evoked vascular response
A
Light
Faster cap
Slower cap
Flux (RBC/s)
Time (s)
B
Light
Faster cap
Slower cap
Flux change (%)
Time (s)
C
ns
Flux change (%)
Cap 1 (slower)
Cap 2 (faster)
D
Light
All capillaries
Flux change (%)
Time (s)
Non-connected capillaries (velocity)
All capillaries (velocity)
Light-evoked vascular response
Velocity change
Light-evoked vascular response
E
Light
Faster cap
Slower cap
Velocity (mm/s)
Time (s)
F
Light
Faster cap
Slower cap
Velocity change (%)
Time (s)
G
ns
Velocity change (%)
Cap 1 (slower)
Cap 2 (faster)
H
Light
All capillaries
Velocity change (%)
Time (s)

71

Supplementary Figure 5. Light stimulation increases blood cell flux and velocity in non-connected capillaries and globally in the retina. (A-D) Blood cell flux (A) and flux change traces over time (B) (average of 15 capillaries per trace, N=5 mice). After light, slower and faster capillaries increase their flux (n=30 capillaries, N=5 mice, linear mixed-effect model analysis, ns: not significant) (C), as well as globally in the retina (D) (average of 74 capillaries per trace, N=9 mice). (E-H) Blood cell velocity (D) and velocity change traces over time (E) (average of 12 capillaries per trace, N=4 mice). After light, slower and faster capillaries increase their velocity (n=24 capillaries, N=4 mice, linear mixed-effect model analysis, ns: not significant) (F), as well as globally in the retina (H) (average of 56 capillaries per trace, N=8 mice). Similar to IPTNT-connected capillaries (see Figure 3F and Supplementary Figure 4E), the maximum and minimum responses after light were calculated for slower and faster capillaries, respectively. Source data are provided as a Source Data file.

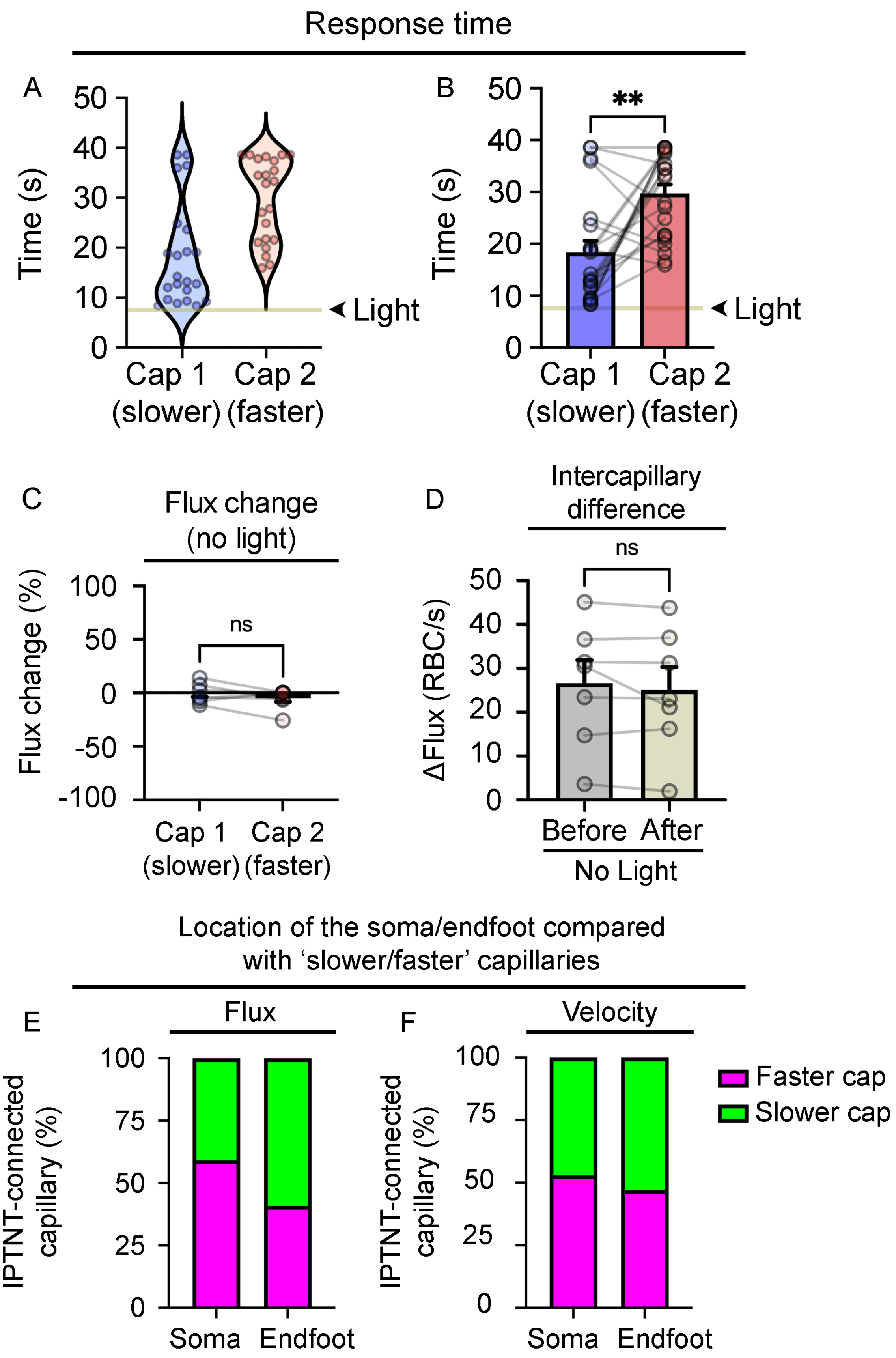
Response time
A
Time (s)
50
40
30
20
10
0
Light
Cap 1 (slower)
Cap 2 (faster)
B
**
Time (s)
Light
Cap 1 (slower)
Cap 2 (faster)
C
Flux change (no light)
Flux change (%)
100
50
0
-50
-100
ns
Cap 1 (slower)
Cap 2 (faster)
D
Intercapillary difference
ns
ΔFlux (RBC/s)
Before
After
No Light
Location of the soma/endfoot compared with 'slower/faster' capillaries
E
Flux
IPTNT-connected capillary (%)
100
75
50
25
0
Soma
Endfoot
F
Velocity
IPTNT-connected capillary (%)
Soma
Endfoot
Faster cap
Slower cap

85

Supplementary Figure 6. Light evokes quicker vascular changes in slower IPTNT-connected capillaries than in faster ones. (A-B) Response-time analysis for slower and faster IPTNT-connected capillaries showing the distribution of the time response (A, violin plot). Slower capillaries respond more quickly than faster capillaries to light stimulation (B, n=44 capillaries, N=7 mice, linear mixed-effect model analysis, **p=0.001). (C-D) Control experiment without light comparing recordings before and after ~10 sec leads to no blood cell flux changes (C, for comparison purposes, same y-axis as figure 3F; n=14 capillaries, N=5 mice, linear mixed-effect model analysis, ns: not significant) nor intercapillary differences changes (D, n=14 capillaries, N=5 mice, two-tailed paired Student's *t*-test, ns: not significant). (E-F) Graphs showing that the location of the soma and endfoot were not particularly associated with capillaries with faster/slower blood cell flux (E) or velocity (F). Source data are provided as a Source Data file.

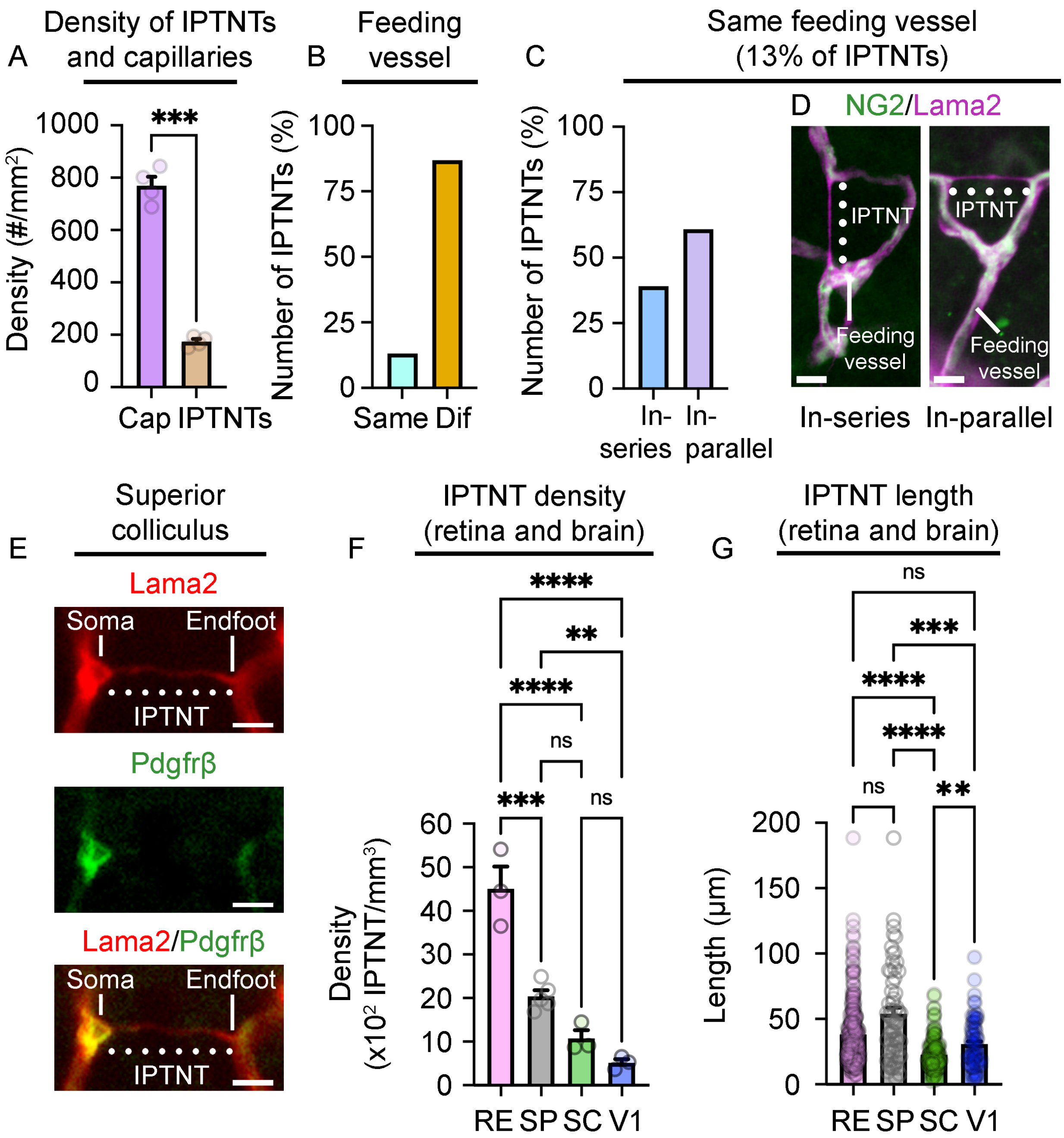

<u>Supplementary Figure 7. IPTNT's features in the retina and brain.</u> (A) Analysis of the density of IPTNTs vs. capillary segments in the retina shows a proportion of 1 IPTNT to 4 capillary segments (n=11006 segments, n=2365 IPTNTs, N=4 mice, linear mixed-effect model analysis, ***p=0.0005). (B-D) IPTNT-connected capillaries do not share the same feeding vessel the majority of the time (87% different feeding vessel vs. 13% same feeding vessel) (B), and those fed by the same vessel

were arranged more in-parallel (61% in-parallel vs. 39% in-series) (C) (n=106 IPTNT-connected capillaries, N=4 mice). Examples of IPTNT-connected capillaries with the same feeding vessel (D). (E-G) The retinorecipient region of the mouse brain, the superior colliculus (E), and visual cortex (V1) also presented IPTNTs connecting capillaries, but in less number (F, in volume units) (n=1727 IPTNTs, N=3 mice in RE group; n=445 IPTNTs, N=5 mice in SP group; n= 163 IPTNTs, N=3 mice in SC group; n=79 IPTNTs, N=3 mice in V1 group, one-way ANOVA Tukey's test test, **$p=0.006$, ***$p=0.0001$, ****$p<0.0001$, ns: not significant) and length (G) (n=292 IPTNTs, N=3 mice in RE group; n=61 IPTNTs, N=3 mice in SP group; n=154 IPTNTs, N=3 mice in SC group; n=79 IPTNTs, N=3 mice in V1 group, ANOVA Kruskal-Wallis test, **$p=0.007$, ***$p<0.0006$, ****$p<0.0001$, ns: not significant) compared to the retina. Scale bar in C, D, E = 50 µm; in K, L, and C, E inset = 10 µm; in D inset = 20 µm. RE: retina; SE: superficial plexus; SC: superior colliculus; V1: visual cortex. Source data are provided as a Source Data file.

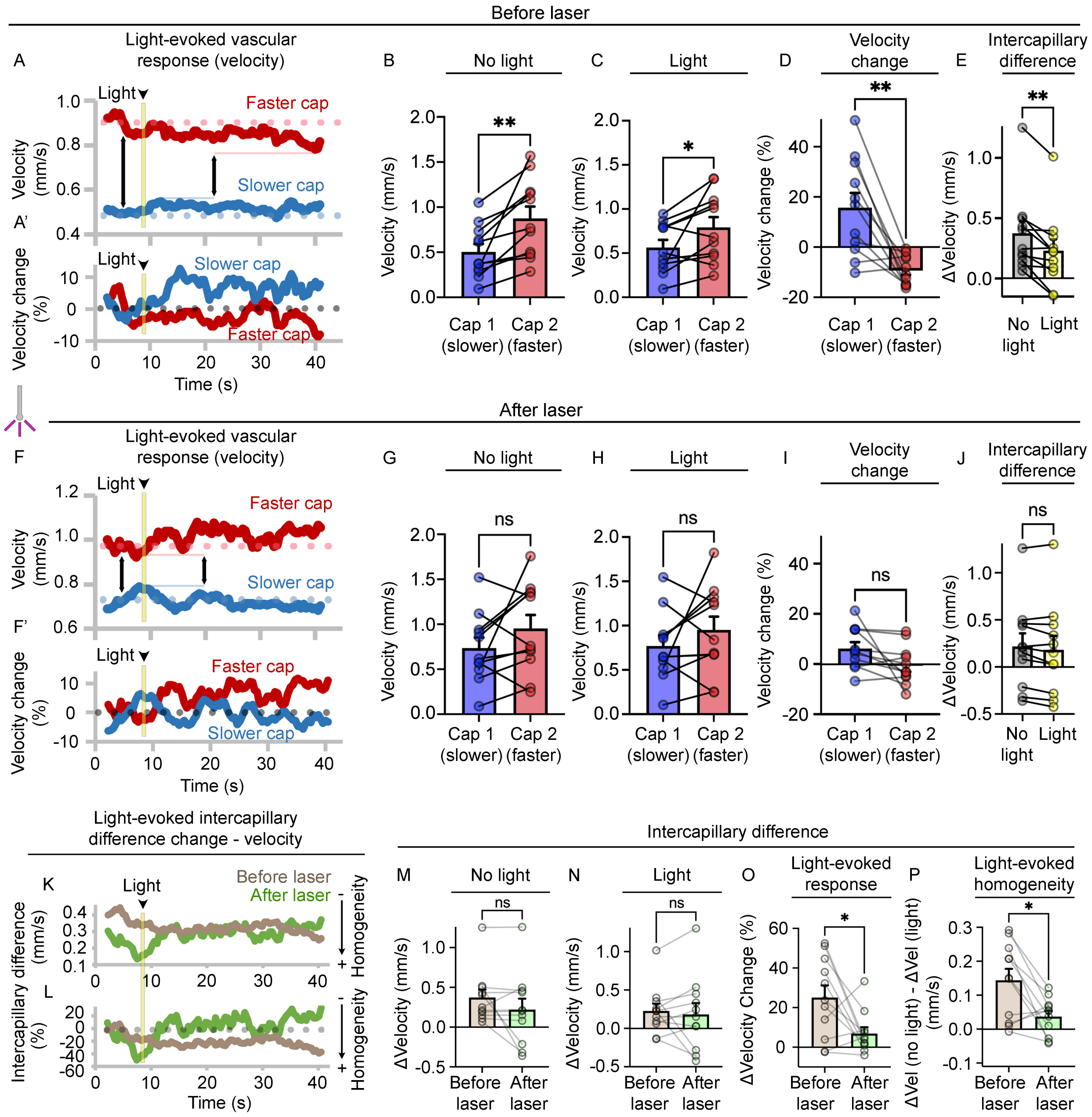

Supplementary Figure 8. Targeted IPTNT ablation disrupts blood velocity patterns.

(A-A') Light-evoked velocity response (A) and velocity change (A') show the ability of slower capillaries to increase their blood cell velocity (blue trace) and faster capillaries to decrease it (red trace) in response to light stimulation (arrowheads) (average of 11 capillaries per trace, N=7 mice; double-headed arrows indicate intercapillary difference before and after light). (B-E) Maximum

response graphs show blood cell velocity for the slower (blue) and faster (red) capillary before IPTNT ablation prior to (B) and after (C, D) light stimulus. Prior to light, IPTNT-connected vessels showed a significant velocity difference (B), which is reduced, but still significant, after light stimulation (n=22 capillaries, N=7 mice, linear mixed-effect model analysis, *p=0.037, **p=0.004) (C) by the selective increase of the velocity of blood cells in slower capillaries (blue, D) and the decrease of blood cell velocity in faster vessels (red, D) (n=22 capillaries, N=7 mice, linear mixed-effect model analysis, **p=0.002). The intercapillary difference graph shows a homogeneity capacity of the IPTNT-connected vessels evoked by light stimuli (n=22 capillaries, N=7 mice, two-tailed paired Student's *t*-test, **p=0.002) (E). (F-F') Laser-induced IPTNT damage eliminated the ability of slower capillaries to increase their blood cell velocity (F-F', blue trace) and faster capillaries to decrease their blood cell velocity (F-F', red trace) in response to light stimulation (arrowheads) (average of 11 capillaries per trace, N=7 mice; double-headed arrows indicate intercapillary difference before and after light). (G-H) Laser-induced IPTNT damage reduced the difference in velocity between connected capillaries before light (G), with no difference after light stimulation (n=22 capillaries, N=7 mice, linear mixed-effect model analysis, ns: not significant) (H). (I-J) IPTNT damage eliminates the ability of slower capillaries to increase their blood cell velocity (blue, I) and faster capillaries decrease it (red, I) after light (n=22 capillaries, N=7 mice, linear mixed-effect model analysis, ns: not significant). Accordingly, the intercapillary difference is similar prior to and after the light stimulus (n=22 capillaries, N=7 mice, two-tailed paired Student's *t*-test, ns: not significant) (J). (K, L) Graphs comparing the difference in velocity between capillaries (K) and percent-of-change difference (L) between connected capillaries over time before (brown trace) and after IPTNT ablation (green trace). IPTNT ablation markedly eliminates

the homogeneity ability after light stimulation (arrowhead) (average of 11 capillaries per trace, N=7 mice). (M-P) Graphs show laser-induced IPTNT damage leads to a reduced light-evoked response (O), and a markedly reduced ability to homogenize after light stimulation (P) but not to significant intercapillary velocity difference before (M) or after light stimulation (N) (n=22 capillaries, N=7 mice, two-tailed paired Student’s *t*-test, *p<0.05, ns: not significant). Source data are provided as a Source Data file.

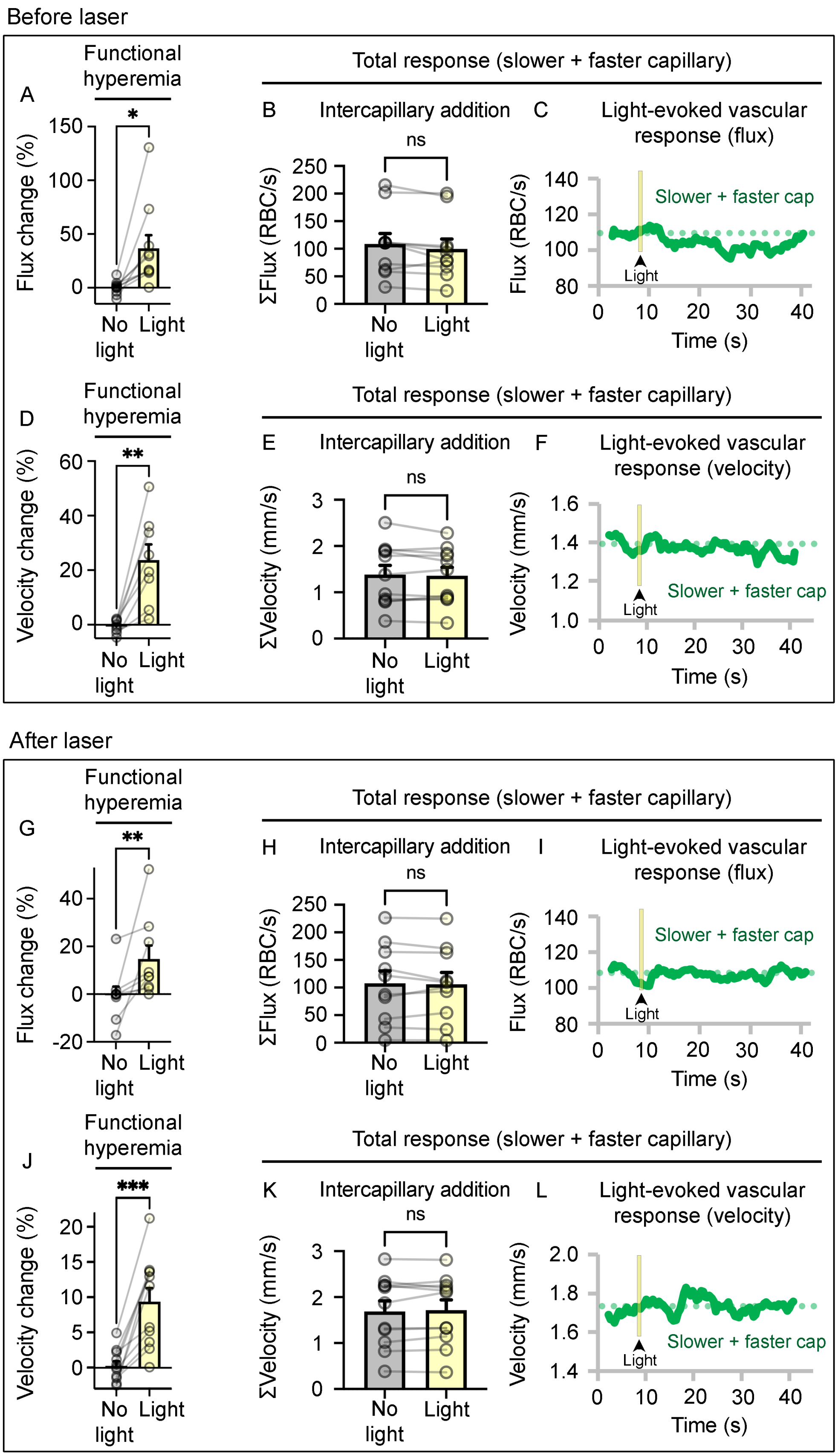
Before laser
A
Functional hyperemia
Flux change (%)
150
100
50
0
*
No light
Light
Total response (slower + faster capillary)
B
Intercapillary addition
ns
ΣFlux (RBC/s)
250
200
150
100
50
0
No light
Light
C
Light-evoked vascular response (flux)
Flux (RBC/s)
140
120
100
80
Slower + faster cap
Light
0
10
20
30
40
Time (s)
D
Functional hyperemia
Velocity change (%)
60
40
20
0
**
No light
Light
Total response (slower + faster capillary)
E
Intercapillary addition
ns
ΣVelocity (mm/s)
3
2
1
0
No light
Light
F
Light-evoked vascular response (velocity)
Velocity (mm/s)
1.6
1.4
1.2
1.0
Slower + faster cap
Light
0
10
20
30
40
Time (s)
After laser
G
Functional hyperemia
Flux change (%)
40
20
0
-20
**
No light
Light
Total response (slower + faster capillary)
H
Intercapillary addition
ns
ΣFlux (RBC/s)
250
200
150
100
50
0
No light
Light
I
Light-evoked vascular response (flux)
Flux (RBC/s)
140
120
100
80
Slower + faster cap
Light
0
10
20
30
40
Time (s)
J
Functional hyperemia
Velocity change (%)
20
15
10
5
0
***
No light
Light
Total response (slower + faster capillary)
K
Intercapillary addition
ns
ΣVelocity (mm/s)
3
2
1
0
No light
Light
L
Light-evoked vascular response (velocity)
Velocity (mm/s)
2.0
1.8
1.6
1.4
Slower + faster cap
Light
0
10
20
30
40
Time (s)

Supplementary Figure 9. Light solely changes the patterning flow without a net increase in blood flow flux/velocity in IPTNT-connected capillaries. (A-F) Light evoked functional hyperemia (i.e., positive % vascular change) in ~half of the capillaries (slower ones) but evoked no net changes (slower capillary changes + faster capillary changes) in blood cell flux (A, n=10 out of 20 capillaries, N=6 mice, paired Student's *t*-test, *p=0.012; B, n=20 capillaries, N=6 mice, two-tailed paired Student's *t*-test, ns: not significant; C, average of 20 capillaries, N=6 mice; arrowhead indicates light stimulus) or velocity (D, n=8 out of 22 capillaries, N=7 mice, two-tailed paired Student's *t*-test, **p=0.002; E, n=22 capillaries, N=7 mice, two-tailed paired Student's *t*-test, ns: not significant; F, average of 22 capillaries, N=7 mice; arrowhead indicates light stimulus). (G-L) IPTNT ablation did not lead to significant changes in the number of capillaries presenting functional hyperemia (although it was not specific for slower capillaries) or net changes in cell blood flow (G, n=9 out of 20 capillaries, N=6 mice, two-tailed Wilcoxon matched-pairs signed-rank test, **p=0.008; H, n=20 capillaries, N=6 mice, two-tailed paired Student's *t*-test, ns: not significant; I, average of 20 capillaries, N=6 mice; arrowhead indicates light stimulus) or velocity (J, n=11 out of 22 capillaries, N=7 mice, two-tailed paired Student's *t*-test, ***p=0.0003; K, n=22 capillaries, N=7 mice, two-tailed paired Student's *t*-test, ns: not significant; L, average of 22 capillaries, N=7 mice; arrowhead indicates light stimulus). Source data are provided as a Source Data file.

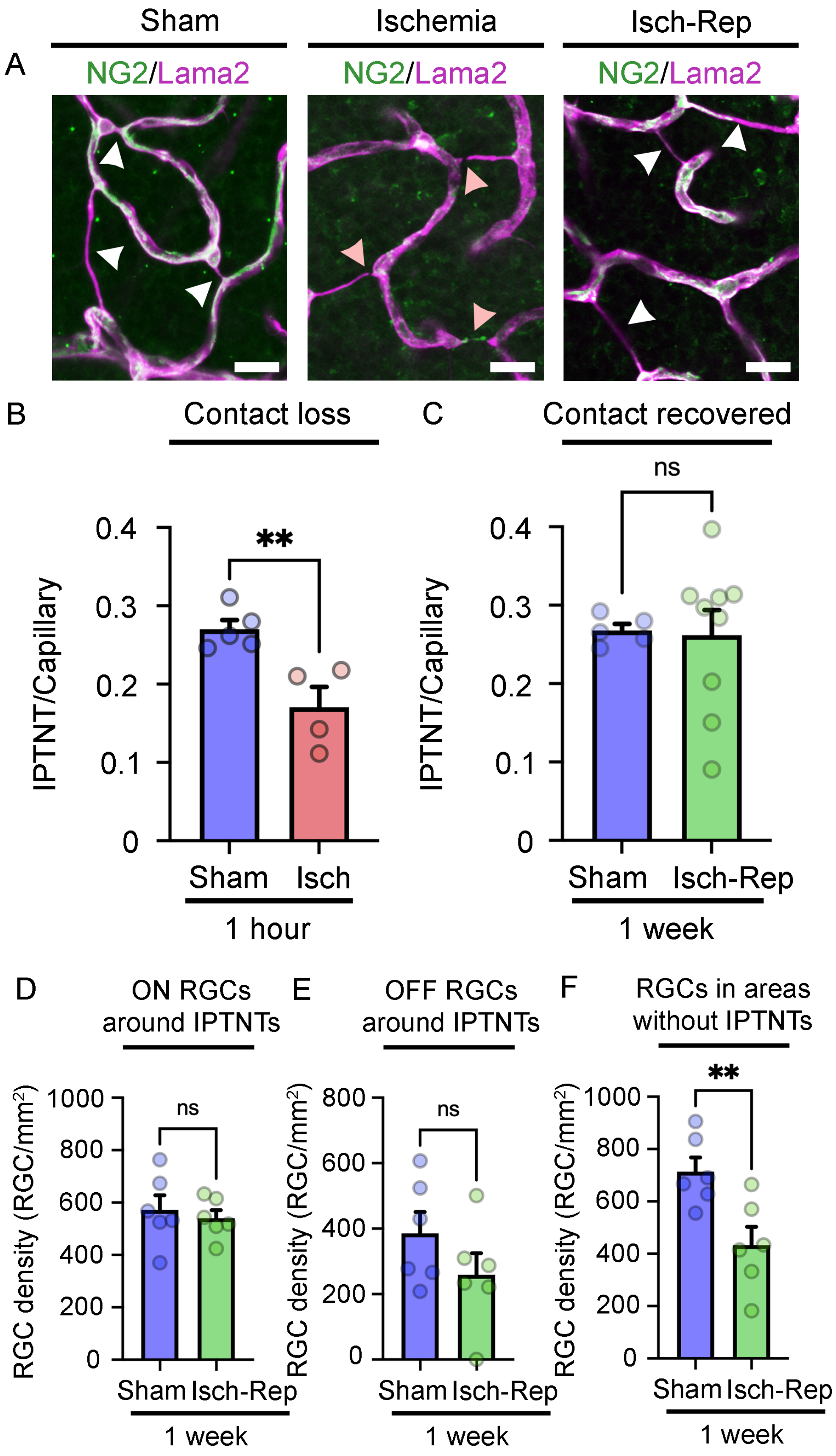
Sham
Ischemia
Isch-Rep
A
NG2/Lama2
NG2/Lama2
NG2/Lama2
B
Contact loss
C
Contact recovered
ns
**
IPTNT/Capillary
0.4
0.3
0.2
0.1
0
Sham
Isch
1 hour
Sham
Isch-Rep
1 week
D
ON RGCs around IPTNTs
E
OFF RGCs around IPTNTs
F
RGCs in areas without IPTNTs
RGC density (RGC/mm²)
1000
800
600
400
200
0
Sham
Isch-Rep
1 week

172

Supplementary Figure 10. Ischemia injury leads to a temporary contact loss of the IPTNTs. (A) Flat-mounted retinas labeled with antibodies against Lama2 (purple) and NG2 (green) showed ruptured IPTNTs (red arrowheads) after 60 minutes of ischemia compared to sham retinas. IPTNTs connect distal vessels after one-week reperfusion. (B-C) After ischemia, the ratio IPTNT:capillary decreases as a consequence of the IPTNT rupture (B, n=12714 segments, n=4152 IPTNTs, N=5 mice in sham group; n=9271 segments, n=2939 IPTNTs, N=4 mice in isch group, two-tailed Student’s *t*-test, **p=0.007), which returned to sham values after one-week reperfusion (C, n=12467 segments, n=4102 IPTNTs, N=5 mice in sham group; n=20352 segments, n=7319 IPTNTs, N=9 mice in isch-rep group, two-tailed Student’s *t*-test, ns: not significant). (D-E) After one-week reperfusion, the number of ON (D, n=218 α-cells, N=6 mice in sham group; n=365 α-cells, N=6 mice in isch-rep group, two-tailed Student’s *t*-test, ns: not significant) and OFF RGCs (E, n=146 α-cells, N=6 mice in sham group; n=190 α-cells, N=6 mice in isch-rep group, two-tailed Student’s *t*-test, ns: not significant) around IPTNT-connected capillaries was similar to sham controls. (F) Areas without IPTNTs presented a reduced number of RGCs after one-week reperfusion (n=247 α-cells, N=6 mice in sham group; n=123 α-cells, N=6 mice in sham group, two-tailed Student’s *t*-test, **p=0.0097). Scale bars in A = 20 μm. Source data are provided as a Source Data file.

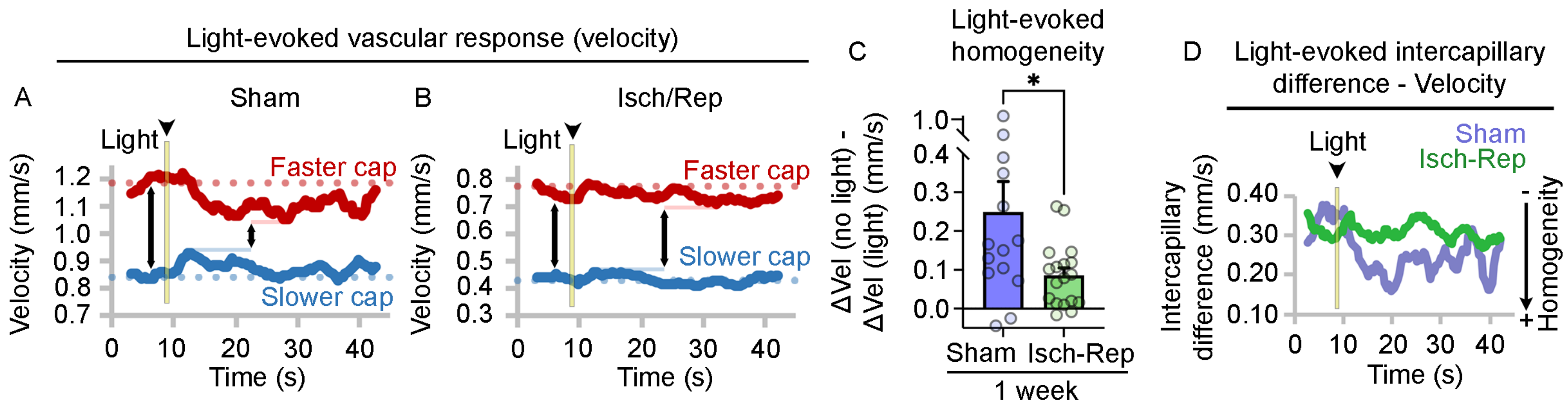

Supplementary Figure 11. Ischemia-reperfusion injury leads to the loss of light-evoked velocity homogeneity.

(A-B) Longitudinal light-evoked capillary velocity changes for sham (A) and ischemia-reperfusion (Isch-Rep) (B) mice. Ischemia-reperfusion eliminated the ability of slower capillaries to increase their blood cell velocity (blue traces) and of faster capillaries to decrease their velocity (red traces) after light stimulation (arrowhead) (average of 18 capillaries per trace, N=4 mice) compared to sham mice (average of 14 capillaries per trace, N=4 mice; double-headed arrows indicate intercapillary difference before and after light). (C-D) Light-evoked homogeneity graph (C) and velocity difference (D) between connected capillaries over time show a reduced ability for IPTNTs of Isch-Rep mice to homogenize their blood cell velocity (i.e., decreasing intercapillary difference) after light stimulation (arrowhead) (green trace) relative to sham controls (blue trace) (n=28 capillaries, N=4 mice in sham group; 36 capillaries, N=4 mice in isch-rep group, two-tailed Student's *t*-test, *p=0.031). Source data are provided as a Source Data file.

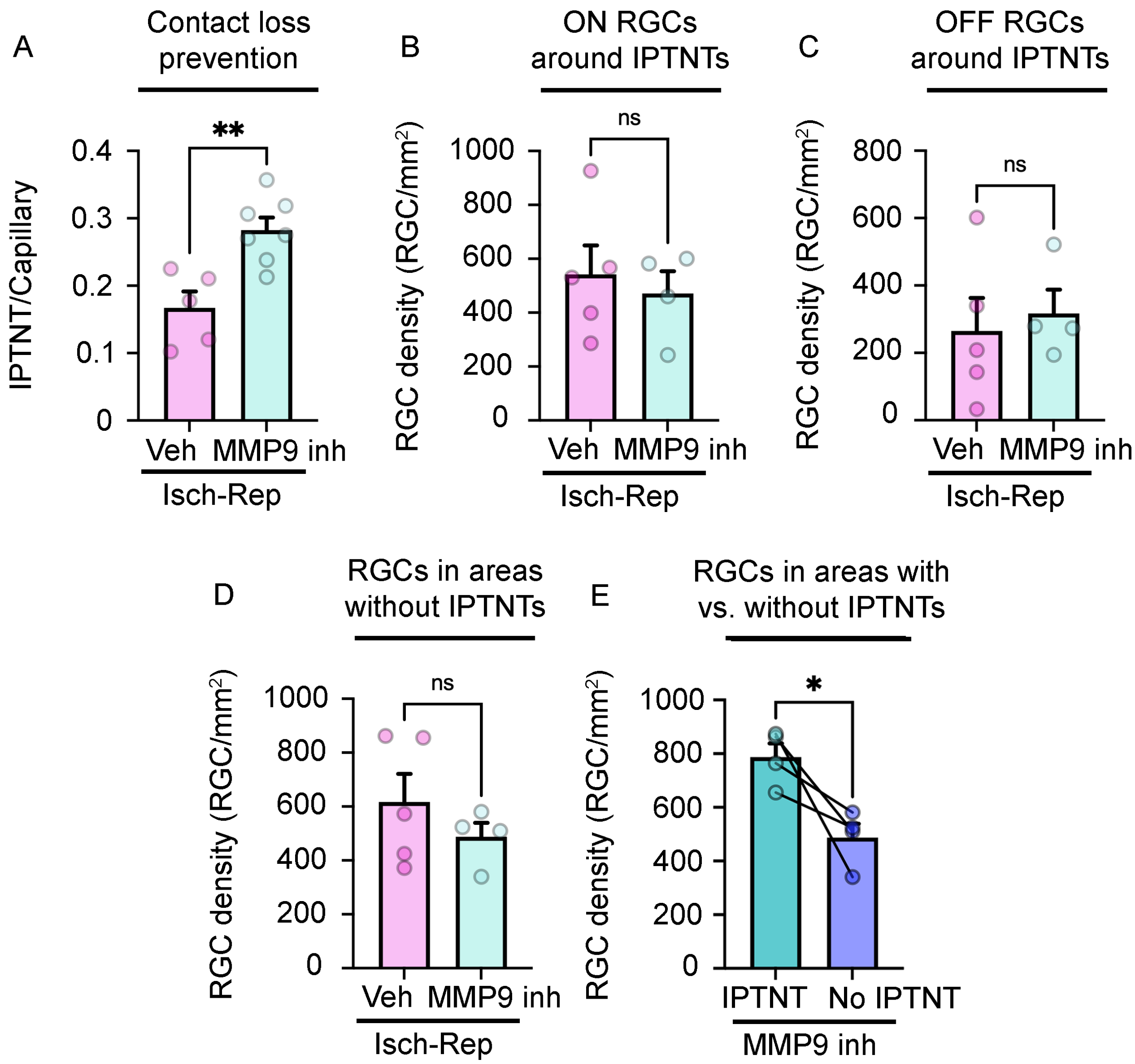

Supplementary Figure 12. Inhibition of MMP9 activation prevents IPTNT rupture during ischemia injury. (A) IPTNT density analysis in ischemic/reperfusion + vehicle and ischemic/reperfusion + MMP9 inhibitor retinas shows that MMP9 inhibitor prevented the loss of the density of IPTNTs connecting capillaries and, consequently, prevented the decrease of the ratio IPTNT:capillary (n=13089 segments, n=4932 IPTNTs, N=5 mice in veh group; n=17749 segments, n=7862 IPTNTs, N=7 mice in MMP9 inh group, two-tailed Student's *t*-test, **p=0.003). (B-D) The number of ON (B, n=193 α-cells, N=5 mice in veh group; n=139 α-cells, N=4 mice in

MMP9 inh group, two-tailed Student's *t*-test, ns: not significant) and OFF RGCs (C, n=105 α-cells, N=5 mice in veh group; n=97 α-cells, N=4 mice in MMP9 inh group, two-tailed Student's *t*-test, ns: not significant) around IPTNT-connected capillaries was similar between groups. Areas without IPTNTs also presented a similar number of RGCs between groups (n=133 α-cells, N=5 mice in veh group; n=128 α-cells, N=4 mice in MMP9 inh group, two-tailed Student's *t*-test, ns: not significant) (D). (E) The MMP9 inhibitor prevented the loss of RGCs only in areas with IPTNTs (n=236 α-cells, N=4 mice in IPTNT group; n=128 α-cells, N=4 mice in No IPTNT group, linear mixed-effect model analysis, *p=0.046). Source data are provided as a Source Data file.

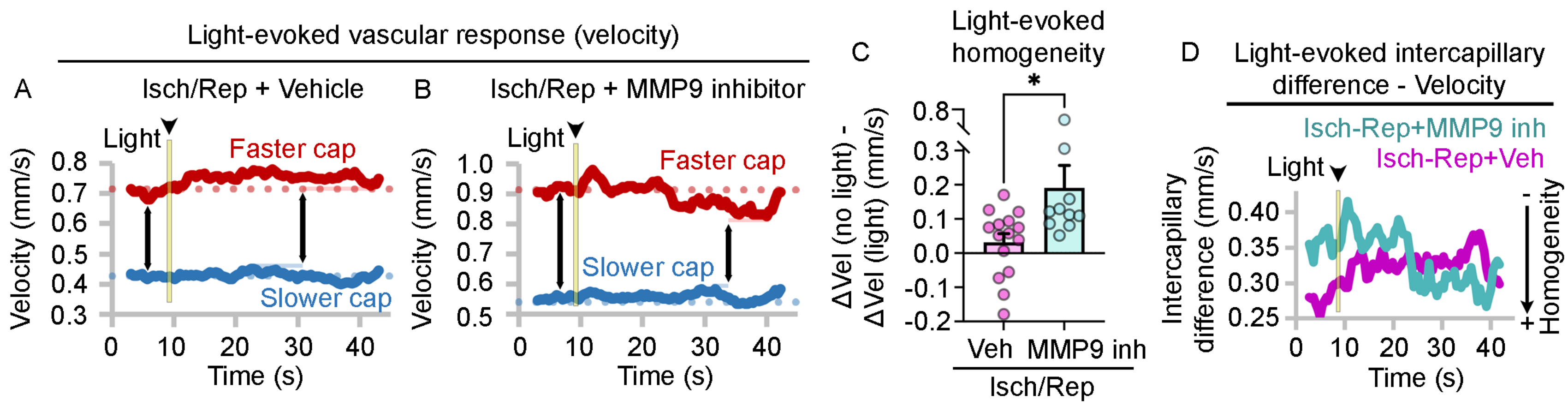

Supplementary Figure 13. MMP9 inhibitors prevent the loss of light-evoked velocity homogeneity after ischemia-reperfusion injury. (A-B) Changes over time in capillary velocity following a flash light stimulus (arrowhead) in mice subjected to Isch-Rep + vehicle (A) or Isch-Rep + MMP9 inhibitor (B) show preservation of the ability of faster capillaries to decrease their blood cell velocity (red traces) and of slower capillaries to increase their velocity (blue traces) after light (average of 10 capillaries per trace, N=4 mice) compared to Isch-Rep + vehicle mice (average of 15 capillaries per trace, N=4 mice; double-headed arrows indicate intercapillary difference before and after light). (C-D) Light-evoked homogeneity graph (C) and velocity difference (D) between connected capillaries over time show that the prevention of IPTNT rupture with MMP9 inhibitors prevented the loss of the ability to homogenize blood cell velocity between capillaries after light stimulation (arrowhead) (cyan trace) relative to vehicle controls (pink trace) (n=30 capillaries, N=4 mice in veh group; n=20 capillaries, N=4 mice in MMP9 inh group, two-tailed Student's *t*-test, *p=0.017). Source data are provided as a Source Data file.

A Different neurons contributing to each area

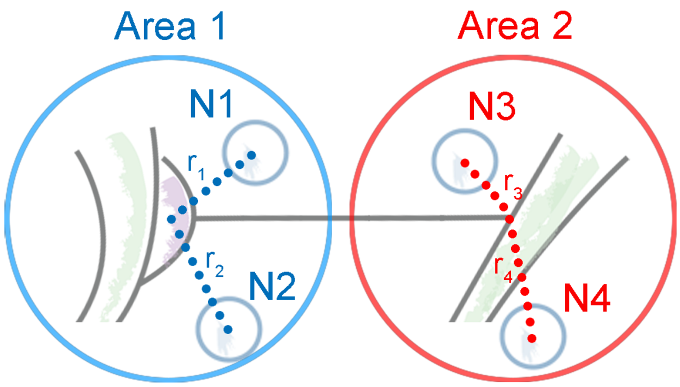

Neuron’s contribution to area polarity index (N) = $e^{-\left(\frac{r^2}{4D^*}\right)}$

Area 1 index = N1 + N2

Area 2 index = N3 + N4

Index associated with a pair of capillaries = Area 1 index - Area 2 index

B A neuron contributing unequally to both areas

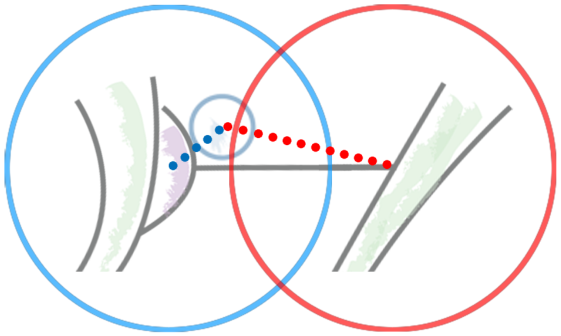

C A neuron contributing equally to both areas

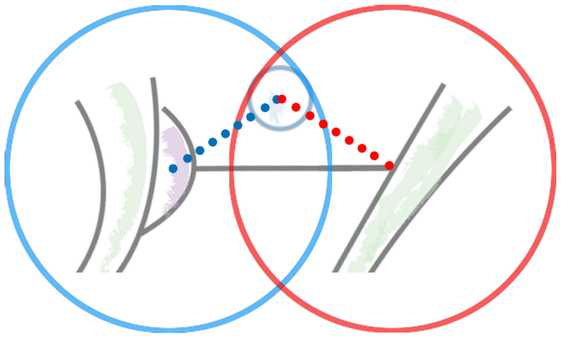

Supplementary Figure 14. Polarity index analysis. (A) Diagram describing the method used to calculate a neuron's contribution to each area (N) as well as the index of each area and the index associated with two IPTNT-connected capillaries (Area 1 index – Area 2 index). D* is the effective diffusion coefficient through the extracellular space of glutamate (i.e., 450 $\mu m^2/s$)[4,5]. (B-C) Particular situations where the same neuron contributes unequally (B) and equally (C) to both areas. Only 2% of the RGCs analyzed contribute equally to both areas.

References

1. Krieger, B., Qiao, M., Rousso, D. L., Sanes, J. R. & Meister, M. Four alpha ganglion cell types in mouse retina: Function, structure, and molecular signatures. *PLOS ONE* **12**, e0180091 (2017).
2. Rousso, D. L. *et al.* Two Pairs of ON and OFF Retinal Ganglion Cells Are Defined by Intersectional Patterns of Transcription Factor Expression. *Cell Rep.* **15**, 1930–1944 (2016).
3. Tran, N. M. *et al.* Single-Cell Profiles of Retinal Ganglion Cells Differing in Resilience to Injury Reveal Neuroprotective Genes. *Neuron* **104**, 1039-1055.e12 (2019).
4. Zheng, K., Scimemi, A. & Rusakov, D. A. Receptor actions of synaptically released glutamate: the role of transporters on the scale from nanometers to microns. *Biophys. J.* **95**, 4584–4596 (2008).
5. Zheng, K. *et al.* Nanoscale diffusion in the synaptic cleft and beyond measured with time-resolved fluorescence anisotropy imaging. *Sci. Rep.* **7**, 42022 (2017).
