# Supplementary material for: Blood flow patterns in mice are regulated by interpericyte tunneling nanotubes connecting functionally-opposite neuronal areas
Source: Nat Commun. 2026 Apr 13;17:5141. doi: 10.1038/s41467-026-71804-2 (PMC13250102; doi:10.1038/s41467-026-71804-2)
Supplement: Supplementary file 2 — Description of Additional Supplementary Files [file 41467_2026_71804_MOESM2_ESM.pdf]

Description of Additional Supplementary Files

Movie S1. IPTNTs connect capillaries with different blood cell flow/velocity. *In vivo* imaging of an IPTNT (white dotted line) visualized with intravitreal injection of TRITC-lectin (red) linking pericytes on distal capillaries labeled using IP fluorescein (green). The IPTNT connects a capillary showing slower blood flow/velocity (right vessel) with a capillary presenting faster blood flow (left vessel). The movie presents low-temporal resolution frames with line-scanning fast high-temporal resolution (i.e., ~800 Hz, insets). Shadows against the fluorescent plasma indicate individual RBCs (yellow dots). After light stimulation, there was an increase and a decrease in blood flow in the slower and faster capillary, respectively.

Movie S2. *In vivo* functional assessment of neurons surrounding IPTNT-connecting capillaries. *In vivo* imaging of an IPTNT (white dotted line) visualized with intravitreal injection of TRITC-lectin (red), blood flow with IP fluorescein (green), and RGCs (circles) expressing the genetically encoded calcium indicator GCaMP6f (green). Light was presented, and calcium changes were calculated based on signal extraction methods[6] that allow the characterization of the RGCs. Importantly, since unfocused RGCs may lead to misinterpretation of the results, we recorded light-evoked RGC responses after focusing on each IPTNT-connected capillary area.

Movie S3. *In vivo* z-stack of the retina of a mouse. *In vivo* imaging of three IPTNTs (arrowheads) visualized in the superficial plexus of a mouse retina after an intravitreal injection of TRITC-lectin (red).

<u>Movie S4. *In vivo* light-evoked diameter changes in IPTNT-connected capillaries.</u> *In vivo* imaging of two IPTNT-connected capillaries visualized with TRITC-lectin (mural labeling) and FITC (lumen labeling) showing simultaneous and opposite light-evoked diameter changes. Scale bar = 2 µm.

Supplementary Code 1: Code and two tutorial videos with file examples to use with the code.
