# Supplementary material for: Blood flow patterns in mice are regulated by interpericyte tunneling nanotubes connecting functionally-opposite neuronal areas
Source: Nat Commun. 2026 Apr 13;17:5141. doi: 10.1038/s41467-026-71804-2 (PMC13250102; doi:10.1038/s41467-026-71804-2)
Supplement: Supplementary file 7 — Reporting Summary [file 41467_2026_71804_MOESM7_ESM.pdf]

nature portfolio

Corresponding author(s): Luis Alarcon-Martinez

Last updated by author(s): Mar 5, 2026

Reporting Summary

Nature Portfolio wishes to improve the reproducibility of the work that we publish. This form provides structure for consistency and transparency in reporting. For further information on Nature Portfolio policies, see our Editorial Policies and the Editorial Policy Checklist.

Statistics

For all statistical analyses, confirm that the following items are present in the figure legend, table legend, main text, or Methods section.

n/a	Confirmed	
[ ]	[x]	The exact sample size (n) for each experimental group/condition, given as a discrete number and unit of measurement
[ ]	[x]	A statement on whether measurements were taken from distinct samples or whether the same sample was measured repeatedly
[ ]	[x]	The statistical test(s) used AND whether they are one- or two-sided<br>*Only common tests should be described solely by name; describe more complex techniques in the Methods section.*
[ ]	[x]	A description of all covariates tested
[ ]	[x]	A description of any assumptions or corrections, such as tests of normality and adjustment for multiple comparisons
[ ]	[x]	A full description of the statistical parameters including central tendency (e.g. means) or other basic estimates (e.g. regression coefficient) AND variation (e.g. standard deviation) or associated estimates of uncertainty (e.g. confidence intervals)
[ ]	[x]	For null hypothesis testing, the test statistic (e.g. F, t, r) with confidence intervals, effect sizes, degrees of freedom and P value noted<br>*Give P values as exact values whenever suitable.*
[x]	[ ]	For Bayesian analysis, information on the choice of priors and Markov chain Monte Carlo settings
[x]	[ ]	For hierarchical and complex designs, identification of the appropriate level for tests and full reporting of outcomes
[x]	[ ]	Estimates of effect sizes (e.g. Cohen's d, Pearson's r), indicating how they were calculated

Our web collection on statistics for biologists contains articles on many of the points above.

Software and code

Policy information about availability of computer code

Data collection	Olympus software (FV30S-SW, Olympus, Tokyo, Japan) or Zeiss software (Zen 2.0, Zeiss, Oberkochen, Germany) was used for in vivo imaging, including pericytes, IPTNTs, acquisition of blood flow, and calcium changes. Leica software (LAS X software, Wetzlar, Germany) was used for ex vivo imaging. Labchart 8 (ADInstruments, Colorado Springs, CO) was used to generate light stimuli during in vivo imaging.
Data analysis	Haemodynamic measurements (i.e., blood cell flux, velocity, and capillary diameter) were measured using ImageJ 1.53 (National Institute of Health). Data was exported to R and computed over time and as % of change related to values before the stimulus. In vivo calcium changes were calculated with Image J and based on signal extraction methods (Longden, T. A. et al. Sci. Adv. 7, eabh0101, 2021). ImageJ was used for vessel analyses, IPTNT and cell number, length measurement, polarity index analysis, and MMP9 expression analysis. Microsoft Excel 16.0 was used for the analysis of the data and for the generation of the traces. Statistical analysis was performed with Prism 10 (GraphPad Software, San Diego, CA).

For manuscripts utilizing custom algorithms or software that are central to the research but not yet described in published literature, software must be made available to editors and reviewers. We strongly encourage code deposition in a community repository (e.g. GitHub). See the Nature Portfolio guidelines for submitting code & software for further information.

Data

Policy information about availability of data

All manuscripts must include a data availability statement. This statement should provide the following information, where applicable:

- Accession codes, unique identifiers, or web links for publicly available datasets
- A description of any restrictions on data availability
- For clinical datasets or third party data, please ensure that the statement adheres to our policy

All the data analyzed in this study, including raw data, are provided in this published article and the supplementary information files. There are no restrictions on data availability. The code used for data analysis is included in the supplementary information files and via a link to Code Ocean.

Research involving human participants, their data, or biological material

Policy information about studies with human participants or human data. See also policy information about sex, gender (identity/presentation), and sexual orientation and race, ethnicity and racism.

Reporting on sex and gender	NA
Reporting on race, ethnicity, or other socially relevant groupings	NA
Population characteristics	NA
Recruitment	NA
Ethics oversight	NA

Note that full information on the approval of the study protocol must also be provided in the manuscript.

Field-specific reporting

Please select the one below that is the best fit for your research. If you are not sure, read the appropriate sections before making your selection.

☒ Life sciences ☐ Behavioural & social sciences ☐ Ecological, evolutionary & environmental sciences

For a reference copy of the document with all sections, see nature.com/documents/nr-reporting-summary-flat.pdf

Life sciences study design

All studies must disclose on these points even when the disclosure is negative.

Sample size	An appropriate sample size was determined by power analysis coupled with experience when the study was designed. For sample size estimates, power calculations were used to determine the number of animals required per group for any given experiment to achieve an 80% power to detect a statistically significant difference at alpha = 0.05. The detailed number of animals used in each group, as well as the number of IPTNTs, capillaries, areas, and cell analyzed, is indicated in the figure legends.
Data exclusions	Recordings that did not allow the visualization of individual red blood cells were not quantified. The exclusion criteria were pre-established. No other data were excluded.
Replication	Regarding potential biological variations between animals, we ensured that each experiment was replicated in multiple mice as indicated in the figure legends. Multiple analyses were replicated by different users.
Randomization	For the ex vivo experiments, we used an unbiased stereological approach based on systematic random sampling. This approach involved randomly placing 3D-disectors (stacks) across the entire retina. This approach ensured that our data was collected randomly from a large population available for analysis, therefore avoiding bias. For in vivo experiments, any IPTNT found in the retinal imaged area was included in the analysis.
Blinding	Investigators performed haemodynamic measurements blinded to the slower/faster vessel. IPTNT density analysis was carried out blinded with an unbiased stereological approach. For the polarity index, all cells in the region of interest were analyzed, and the analysis was performed blinded to either the cell type or to the distance to the IPTNT. Calcium responses and MMP9 expression analysis were not performed with blinding, as analyses were automated.

Reporting for specific materials, systems and methods

We require information from authors about some types of materials, experimental systems and methods used in many studies. Here, indicate whether each material, system or method listed is relevant to your study. If you are not sure if a list item applies to your research, read the appropriate section before selecting a response.

Materials & experimental systems

n/a	Involved in the study
	x Antibodies
x	Eukaryotic cell lines
x	Palaeontology and archaeology
	x Animals and other organisms
x	Clinical data
x	Dual use research of concern
x	Plants

Methods

n/a	Involved in the study
x	ChIP-seq
x	Flow cytometry
x	MRI-based neuroimaging

Antibodies

Antibodies used

The following validated antibodies were used in our study:

Primary Antibodies; Dilution; Catalog #; Company; Lot #

Lama2; 9.6 µg/ml; L0663; Merck-Millipore; 167335
SMI-32; 4 µg/ml; 801701; Biolegend; B353998
Brn3a; 1 µg/ml; SC8429AF488; Santa Cruz Biotechnology; E1122
NG2; 5 µg/ml; AB5320; Merck-Millipore; 3753286
Foxp1; 2 µg/ml; ab16645; Abcam; 1062765-2
Foxp2; 1 µg/ml; ab1307; Abcam; 1020864-4
αSMA; 10 µg/ml; A5228; Sigma; 0000348067
PDGFRb; 0.8 µg/ml; AF1042-SP; R&D Systems; GOV0523031

Secondary Antibodies; Dilution; Catalog #; Company; Lot #

donkey anti-rat 647nm; 6 µg/ml; 712-607-003; Jackson ImmunoResearch; 160581
donkey anti-mouse Cy3; 3 µg/ml; 715-167-003; Jackson ImmunoResearch; 156832
donkey anti-rabbit 488nm; 6.4 µg/ml; 711-547-003; Jackson ImmunoResearch; 159199
donkey anti-rabbit Cy3; 6 µg/ml; 711-167-003; Jackson ImmunoResearch; 158654
donkey anti-goat 488nm; 7.2 µg/ml; 705-547-003; Jackson ImmunoResearch; 156608
donkey anti-mouse 488nm; 7.2 µg/ml; 715-547-003; Jackson ImmunoResearch; 160320

Validation

The antibodies used in our study have been extensively validated by us and others as per the following publications:

1. Lama2:

Alarcon-Martinez L, Shiga Y, Villafranca-Baughman D, Belforte N, Quintero H, Dotigny F, Cueva Vargas JL, Di Polo A. Pericyte dysfunction and loss of interpericyte tunneling nanotubes promote neurovascular deficits in glaucoma. Proc Natl Acad Sci U S A. 2022 Feb 15;119(7):e2110329119. doi: 10.1073/pnas.2110329119. PMID: 35135877; PMCID: PMC8851476.

2. SMI-32

Gallego-Ortega A, Norte-Muñoz M, Di Pierdomenico J, Avilés-Trigueros M, de la Villa P, Valiente-Soriano FJ, Vidal-Sanz M. Alpha retinal ganglion cells in pigmented mice retina: number and distribution. Front Neuroanat. 2022 Dec 1;16:1054849. doi: 10.3389/fnana.2022.1054849. PMID: 36530520; PMCID: PMC9751430.

3. Brn3a:

Lin F, Lin ST, Wang J, Geisert EE. Optimizing retinal ganglion cell nuclear staining for automated cell counting. Exp Eye Res. 2024 May;242:109881. doi: 10.1016/j.exer.2024.109881. Epub 2024 Mar 28. PMID: 38554800; PMCID: PMC11055661.

3. NG2:

Alarcon-Martinez L, Yilmaz-Ozcan S, Yemisci M, Schallek J, Kılıç K, Can A, Di Polo A, Dalkara T. Capillary pericytes express α-smooth muscle actin, which requires prevention of filamentous-actin depolymerization for detection. Elife. 2018 Mar 21;7:e34861. doi: 10.7554/eLife.34861. PMID: 29561727; PMCID: PMC5862523.

4. Foxp1:

Suzuki-Kerr H, Baba Y, Tsuhako A, Koso H, Dekker JD, Tucker HO, Kuribayashi H, Watanabe S. Forkhead Box Protein P1 Is Dispensable for Retina but Essential for Lens Development. Invest Ophthalmol Vis Sci. 2017 Apr 1;58(4):1916-1929. doi: 10.1167/iovs.16-20085.

PMID: 28384713.

5. Foxp2:
Sato C, Iwai-Takekoshi L, Ichikawa Y, Kawasaki H. Cell type-specific expression of FoxP2 in the ferret and mouse retina. Neurosci Res. 2017 Apr;117:1-13. doi: 10.1016/j.neures.2016.11.008. Epub 2016 Nov 22. PMID: 27888071.

6. αSMA:

Alarcon-Martinez L, Yilmaz-Ozcan S, Yemisci M, Schallek J, Kılıç K, Can A, Di Polo A, Dalkara T. Capillary pericytes express α-smooth muscle actin, which requires prevention of filamentous-actin depolymerization for detection. Elife. 2018 Mar 21;7:e34861. doi: 10.7554/eLife.34861. PMID: 29561727; PMCID: PMC5862523.

7. PDGFRb:

Kovács-Öller T, Ivanova E, Szarka G, Tengölics ÁJ, Völgyi B, Sagdullaev BT. Imatinib Sets Pericyte Mosaic in the Retina. Int J Mol Sci. 2020 Apr 5;21(7):2522. doi: 10.3390/ijms21072522. PMID: 32260484; PMCID: PMC7177598.

Animals and other research organisms

Policy information about studies involving animals; ARRIVE guidelines recommended for reporting animal research, and Sex and Gender in Research

Laboratory animals	Experiments included BALB/c mice and mice expressing the red fluorescent protein under control of the NG2 (Cspg4) promoter (NG2-DsRed) (008241, Jackson Laboratory, Bar Harbor, ME) of 2–5 months of age and 20-35 g. Animals were housed in 12 h light/12 h dark cyclic light conditions, with an average in-cage illumination level of 10-20 lux and fed ad libitum.
Wild animals	No wild animals were used in the study.
Reporting on sex	Experiments included adult female and male mice.
Field-collected samples	No field collected samples were used in the study.
Ethics oversight	All animal procedures were approved by the ethical guidelines set by the animal ethics committees at St. Vincent’s Hospital (protocol #: 003.22; VIC, Australia) and The Florey Institute of Neuroscience and Mental Health (protocol #: 22-001-CERA; VIC, Australia), complying with the Prevention of Cruelty to Animals Act and the NHMRC Australian code.

Note that full information on the approval of the study protocol must also be provided in the manuscript.

Plants

Seed stocks	NA
Novel plant genotypes	NA
Authentication	NA
