# Supplementary material for: Blood flow patterns in mice are regulated by interpericyte tunneling nanotubes connecting functionally-opposite neuronal areas
Source: Nat Commun. 2026 Apr 13;17:5141. doi: 10.1038/s41467-026-71804-2 (PMC13250102; doi:10.1038/s41467-026-71804-2)
Supplement: Supplementary file 8 — Transparent Peer Review file [file 41467_2026_71804_MOESM8_ESM.pdf]

Peer Review File

Blood flow patterns in mice are regulated by interpericyte tunneling nanotubes connecting functionally-opposite neuronal areas

Corresponding Author: Dr Luis Alarcon-Martinez

This file contains all reviewer reports in order by version, followed by all author rebuttals in order by version.

Version 0:

Reviewer comments:

Reviewer #1

(Remarks to the Author)
This is an interesting paper that provides new insights into the control of neurovascular coupling and suggests an intriguing hypothesis about the origins and roles of divergent behaviors of neighboring capillaries. In a previous paper, these authors demonstrated the existence of “interpericyte tunneling nanotubes” that connect pericytes associated with capillaries and mediate neurovascular coupling between capillaries that different in flow rate (Alarcon-Martinez et al., Nature, 2020). They also showed that the coupled capillaries exhibit bidirectional changes in behavior following light flash. (…capillary pairs connected by IP-TNTs exhibit opposite responses concomitant with light stimulation: one capillary dilates to increase blood flow, while the other constricts to decrease blood availability.”) In the present paper, they provide further detail on this phenomenon and relate the differences in flow rate to the firing characteristics of neighboring retinal ganglion cells.

I have no technical criticisms but feel that the presentation could be improved. In particular, the analysis of ON and OFF cells seems to be central, yet it is introduced only after the description of light effect on vascular homogenization. I found this very confusing because it seemed to put emphasis on the value of homogenization, which may not in fact be the main issue. The beginning of this section says “…we considered whether this flux pattern might support a particular neuronal organization.” But the results and the later discussion of them raises the possibility that the flux patterns may be not a correlate but rather the direct result of the “neuronal organization.” As noted above, this seems to me to be is the major novel finding.

The simplest way to put it (perhaps a bit easier to understand than the way it is currently stated) is that OFF cells, which are active in the dark, are supplied by faster capillaries than ON cells, which are inactive in the dark. The flash activates ON cells, leading to greater flux in their slow capillaries, and deactivates OFF cells, leading to decreases flux in their fast capillaries. This is fascinating and important and should be introduced earlier and emphasized more. For example you could explain that there are ON and OFF cells that are differentially affected by a flash, then give the flash results, then come back to ask whether the result could be explain by the differential responses.

The ON/OFF issue also raises some new points that are worth discussing. (a) Could something similar underlie heterogeneity in other parts of the brain or is this special? (b) Might heterogeneity-homogeneity be an epiphenomenon of activity patterns? This would differ from the interpretation that the purpose of homogenization is to provide” a better oxygen supply into the extracellular space.” (c) Are there really ON and OFF areas in the retina and if so how large are they? The polarity index is a start but given the mosaic arrangement of ON and OFF RGC types it seems odd; we need more information on this point. (d) The measurement of ON and OFF areas by calcium imaging stands on its own but extension to specific RGC types is puzzling, since the few types tested (F and alpha) comprise only a small fraction of all RGCs. Does their association with ON/OFF areas imply a global principle that has been missed? I can understand that this isn’t the authors’ intended focus, but having introduced it, they are obliged to deal with it the best they can, hopefully with available data.

Specific comments

1) The paper is written in a very compressed manner that makes it difficult for nonspecialists to follow. The format suggests that it was prepared for Nature, which requires this sort of short format. However, Nature Communications does not. I urge

the authors to expand some of the explanations, move a few key panels from the supplement to the main text (for example S5A and S11), and perhaps turn the 4 current figures into 5 or 6. It would also help to show rather than just describe the location of the capillaries being imaged. Small sketches of a cross section and a flat mount could be added to Figure 2 for this purpose. This can all be done while keeping the total length under that of typical papers in this journal – especially if some material already covered in the 2020 paper is compressed.

2) Labeling on and legend for 1D could be expanded since this is the first of many panels of this sort. 100? 1? Flux? Velocity?

3) The significance of the results described in lines 121-135 (on aSMA) is completely obscure to this non-specialist, as is the reason the sentence starting on line 135 ("Before light….") is part of the same paragraph. Some explanation and interpretation is needed.

4) Some explanation of the small arrow in 2C is needed, since it compares different slow and fast time points.

(Remarks on code availability)

Reviewer #2

(Remarks to the Author)
This manuscript by Gardner-Russell et al provides an in-depth view of interpericyte tunneling nanotubule (IPTNT) function in the living retina using in vivo imaging. Using a combination of impressive techniques, including calcium imaging, optical ablations, and ischemia-reperfusion modeling, they conclude that IPTNTs are important for homogenization of flux between capillary segments needed for improved oxygen delivery to retinal tissues. The breadth of work is extensive and the general conclusions are supported by the data. Below, I provide several comments on presentation of the data and how specific details, if presented more clearly, could improve readability and support/refine the conclusions further.

Major:

- The Main figures were rather crowded and would benefit from splitting into separate Figures to better explain the experiments. There are more than 20 small panels in Figure 3 for example, and this makes it hard to appreciate the context and detail of each experiment.

- Some aspects of the supplement would be beneficial to move to main figures because they help understand the model system. For example, Supplementary Fig. 2 provides a better understanding of where IPTNTs are located within the network and would be good to provide as a main figure. Supplementary Fig. 2A is helpful in this regard, but this should be accompanied by a similar histogram of branch orders showing where alpha-SMA reduction occurs (end of transition zone). Prior work from the senior author (Alarcon-Martinez, Elife 2018) has shown that alpha-SMA expression extends up to 6th or 7th branch order in retina with proper fixation, but vessel branch ordering method differs than in the current manuscript. This should be clarified in relation to prior work.

- Capillary diameter information is essential to show clearly with representative examples in the Main figures. There are currently 2 panels in Supplementary Fig. 4. This is a core part of the hypothesis that there is some dynamic regulation of capillary diameter by single IPTNTs, with opposing effects in neighboring capillary branches. The changes are small (5-10%) and proof of the ability to resolve this change is needed. The alternate possibility is that flux homogenization is not dependent on fast pericyte contraction/relaxation, but a result of red blood cell conformation change or increased intraluminal pressure to force cells through the capillaries.

- Figure 1. Please report total flux among the two branches regulated by an IPTNT. Stimulation should lead to functional hyperemia and overall increased total blood flow. However, the graphs seems to suggest that total flux is similar if the slow capillary increases but the fast capillary decreases.

- Clarify whether Figures 1A-G are only for IPTNT contacted capillaries. This data should ideally be accompanied by capillary pairs that are not IPTNT-associated, so there is a comparison.

- Not all readers may be well versed in retinal neuron subtype identification (markers for ON and OFF RGCs). This part of the manuscript could provide more explanation.

- The polarity index metric is only briefly explained in the Results and it is hard to understand what the metric is showing. The Methods and supplement help somewhat, but more explanation in the Main figure and results would help readers follow the analysis.

- The laser ablation is helpful to test the specific role of IPTNT connections. Does this manipulation lead to death of the pericyte? Is there a control for the effects of laser induced injury itself?

- The authors say that the number of ON and OFF RGCs and their function are not changed with ischemia-reperfusion. It is surprising that neurons survive this insult and also maintain their network organization allowing the role of IPTNTs to be tested. Surely, the ischemia-reperfusion is causing major alterations to neuronal health/viability, unless the retina is more resilient than the brain. Some information on the neuronal consequences of the ischemia-reperfusion model would be warranted.

- The authors should be cautious in their interpretations with the MMP9 inhibits. This is a nice experiment, but MMP9 inhibition may have many beneficial effects unrelated to preservation of IPTNTs. It is not an IPTNT-selective experiment.

- The authors wish to extend their conclusions to the brain, but there is limited data. The data provided suggests a much lower density of IPTNTs in the superior colliculus and this is an argument against their contributions to brain capillary flow regulation. Clearly more data is needed, but the authors should be conservative and extrapolate their results to the brain (particularly in the abstract).

Minor/moderate
- The presentation of some scatter plots look blurred (example, Fig 1O, P), while others are not (Fig. 1K,L). The blurring effect makes it hard to see the data points.

- Although the authors mention that capillary flux return the baseline after stimulation, the data shown suggests that they can drift away from baseline (Fig. 1C, C'). Are these ideal representative examples?

(Remarks on code availability)

Reviewer #3

(Remarks to the Author)
The manuscript by Gardner-Russel, Haddara and colleagues presents an interesting concept arguing that retinal blood flow is critically regulated by nanotubes connecting pericytes (IPTNTs). The manuscript includes impressive imagining in vivo and ex vivo and introduces provocative concepts regarding blood flow modulation in the CNS. While the draft doesn't really tackle the concept in the brain, the authors highlight differences in abundance of IPTNTs and suggest that future studies would be warranted to assess the concepts presented here but in the brain. While impressive and provocative, I have several critical concerns and confusions that cast doubts on the conclusions that the authors made. In my opinion, addressing these concerns is critical and necessary. As it stands, the evidence provided here is insufficient to justify the conclusions.

- Capillary order and pericyte type: Studies exploring pericyte physiology have evolved (and many times contradicted one another). One major problem has been whether studies called different cells the same name or called the same cells different names. The capillary order starting with 1st order for the ones branching of the retinal arteriole and increasing till it reaches the venule (as shown in supplemental figure) has been a widely accepted standard. Here, the authors partially, but not sufficiently, address this point in Supplemental figure 2 using the alpha-smooth muscle actin (a-SMA) staining. I like Fig S2 and believe that it is very important and should even be moved to the main figures. All figures in the manuscript, however, should specify the orders of the capillaries where data were collected from. The authors could also include larger FOV images and then a zoomed-in illustration of the IPTNTs of interest.

- Related to the previous point, according to the presented calculations, ~80% of IPTNTs are beyond the transition zone (TZ), and of these IPTNTs more than half are aSMA positive. It seems to me that this cannot be correct based on the examples provided within. This also contradicts the data shown in Suppl. Fig. 2A, B where the authors argue that most nanotubes are around ~3rd to 6th order most of which one would expect absence of aSMA expression (e.g., PMID: 33051294). This is a major point for the authors to consider.

- It should be explicitly explained whether and how IPTNTs are distinct from string capillaries.

- Movie S2: The vessels shown seem too large to be actually capillaries.

- I am quite confused about the estimated high prevalence of IPTNTs and the fact that almost all example images show only very few. The authors should consider revising the calculations or explain why such huge discrepancy exist.

- It remains intriguing how IPTNTs could homogenize the blood flow between connected capillaries. How does this happen? At least this is warranted discussion.

(Remarks on code availability)
I haven't reviewed the code.

Reviewer #4

(Remarks to the Author)
This manuscript focuses on describing the functionality of blood flow regulation by interpericyte tunnelling in ON and OFF

ganglion cell regions of the rodent retina. Using in vivo two-photon laser scanning microscopy, the authors measured retinal blood flow and IPTNT in the retinas of transgenic mice. They demonstrated that IPTNT-connected capillary pairs exhibit greater flow polarity at rest and, during light-evoked functional hyperaemia, the faster capillaries reduce flow speed while the slower capillaries increase it, thereby transitioning from heterogeneous to homogeneous flow. The authors later linked this phenomenon with the functionally opposite ON and OFF neuronal cell regions. Finally, they demonstrated that ischemia-reperfusion damaged IPTNT, which could be repaired by MMP9.
This manuscript provides interesting and novel insights into the regulation of capillary blood flow by IPTNT, which is involved in the fine-tuning of microregional blood flow for different subtypes of neurons. However, the evidence supporting the conclusions on the specific role of IPTNT in regulating retinal blood flow is not strong enough. Furthermore, the underlying mechanism has not been investigated, although different hypotheses have been discussed in the manuscript.

All in vivo experiments were performed under anaesthesia. Previous studies have demonstrated that ketamine and isoflurane can significantly impact neurovascular dynamics by altering vascular tone and neurovascular mechanisms. Additionally, isoflurane is a potent vasodilator. The potentially fine-tuned capillary blood flow by IPTNTs is unnecessarily disturbed by the anaesthesia used by the authors. Further experiments are needed to demonstrate the functionality of IPTNTs in an awake state.

The authors demonstrated that the density of IPTNTs in the retina is approximately 4.5 times greater than in the cortex. Specifically, in Figure 3F, the authors used the superior colliculus as a representative region of the cortex, an area that is rarely studied in relation to cortical blood flow regulation. A more appropriate comparison would be the visual, somatosensory or motor cortex.

Does the eye coverslip interfere with retinal blood flow? There is external pressure pushing towards the mouse's sclera to create sufficient contact area and potentially deform it in order to facilitate two-photon imaging.
The definition of 'non-connected capillaries' as a control for IPTNT-connected capillaries is unclear. If the length of IPTNT varies extensively from less than 5 µm to more than 100 µm (see Supplementary Fig. 3G), how did the authors select the non-connected capillaries in an unbiased manner?
Furthermore, the location of each capillary segment within the vascular tree, local blood pressure and capillary diameter are important factors in determining flux in this capillary, which varies drastically across the entire vasculature. It is therefore interesting to note that non-connected capillaries exhibit more equalised blood flow (Fig. 1K–P) than IPTNT-connected ones. This hardly makes sense. Do the results suggest that retinal capillary blood flow is homogeneous across the entire capillary bed, except in segments connected and polarised by IPTNTs?

On page 5, lines 111–112, the authors claim that 'this suggests that IPTNTs are required for the regulation of local capillary blood flow patterns'. This claim is too strong. In regions without IPTNTs, there may be no need for heterogeneous capillary blood flow. Before discussing neuronal function in the results section, the authors can only claim that IPTNTs create a more heterogeneous pattern of capillary blood flow than regions without IPTNTs; they cannot claim that IPTNTs are 'required for regulation'.

To provide direct evidence that IPTNT regulates local capillary blood flow, light stimulation was applied to the entire region. This also affected the upstream arteries and arterioles, making it difficult to determine the specific function of IPTNT. It would therefore be necessary to manipulate IPTNTs individually and observe paired capillary flow changes, for example using optogenetic stimulation with laser scanning.

In contrast to the conclusions of the Nature Neuroscience article (2024), with the publication code: 39232066, this submission concludes that individual blood vessels are not an accurate indicator of neuronal activity in their immediate vicinity. TThe text fails to provide an explanation for the discrepancy between the results of the two manuscripts.

As stated in the previous Nature paper by the authors, IPTNT was reported at two other plexus layers, which were found to be denser than the superficial layer. Could you please clarify why only the superficial layer was investigated? Please could you confirm whether the functions of IPTNTs are located at the other layers of the retina, where ON and OFF bipolar cells are also present?

Paper 15, line 329, ‘hypoxia can occur at apparently normal blood flow levels’, it has been argued by many papers that baseline blood flow is sufficient for the elevated metabolic needs (PMID: 35995628).

In the ischemia-reperfusion experiments, the authors only compared IPTNT before and after ischemia-reperfusion, and regrowth of IPTNT, as well as the application of MMP9. However, the authors cannot exclude impairment of ordinary pericytes, which are much more abundant than IPTNT. In this case, it is not possible to determine the mechanism by which the recovery of the ordinary pericytes, endothelial cells and neurons could contribute to the regained flow polarity and intercapillary difference.

With regard to the statistical analysis, given the multiple areas, cells and mice involved, the authors should employ a linear mixed effect model to test for statistical significance. It should be noted that the current usage of paired and unpaired t-tests, as well as other relevant tests, assumes that each measurement is independent. However, this is not the case. Therefore, it is imperative that the statistical analysis is redone.

As demonstrated in Figures 2C, 3B and 4I, the authors observed a consistent and gradual decline in flux within the faster capillary up to the conclusion of the recording. In contrast, the slower capillary returned to its baseline much earlier. Have

authors an explanation for this result?

In Fig. 4B, did the authors revisit the same IPTNT after ischemia and further after reperfusion? Why do the diameter and distance of capillaries look very different from each other? Same question with IPTNT.

Fig. 1K representative two-photon xy image, which shows basically clogged and no flow in the capillary at all, which conflicts with the linescan data on the right.

Video 1 shows very well the paired capillaries linked with IPTNT, however, the important and novel discovery of this manuscript is that IPTNTs regulate blood flow during light stimulation from heterogeneity to homogeneity. I would envision the video should include the period after light stimulation and show linescan strips again.

Video 2 provides a clear illustration of the responses of the neighbouring neurons. However, it lacks an indication of which is the ON cell and which is the OFF cell. As you will see from the video, the final figure is not fully explained in the manuscript. Could you please clarify what 'neuronal length' refers to for the neuron in the video that has been specifically marked?

(Remarks on code availability)

Reviewer #5

(Remarks to the Author)
I co-reviewed this manuscript with one of the reviewers who provided the listed reports. This is part of the Nature Communications initiative to facilitate training in peer review and to provide appropriate recognition for Early Career Researchers who co-review manuscripts.

(Remarks on code availability)

Version 1:

Reviewer comments:

Reviewer #1

(Remarks to the Author)
My comments as Reviewer 1 were related more to style and presentation than to substance. The authors have done an excellent job of revising their paper in accordance with my suggestions. This is a terrific paper.

(Remarks on code availability)

Reviewer #2

(Remarks to the Author)
The authors have done a nice job addressing most of my comments. There are a few points related to two of my comments that were not fully addressed in my opinion:

- Related to original comment #3 on capillary diameter information: The authors show graphs, but the readers will want to see high-resolution, representative images of the actual changes in the diameter of the capillaries. This would provide the proof of differential control of diameter between two vessels contacted by an IPTNT that underlies flux homogenization. The authors say that the resolution to detect it is sufficient, and images should be provided. There is a supplementary movie, but the resolution is too low to see this change. I recommend a supplementary figure that clearly demonstrates this diameter change happening, preferably with multiple examples.

- Related to original comment #4 on total flux between capillary branches: I was expecting to see an increase of total flux with functional hyperemia, because retinal arterioles dilate in response to light stimulation. I think the authors need to clarify why this total blood flow increase is not seen, which is inconsistent with what we expect with functional hyperemia.

(Remarks on code availability)

Reviewer #3

(Remarks to the Author)
In the revised manuscript, the authors have done a good job addressing the comments I raised earlier. In particular, I find that the modifications of figures and text help clear confusions in the original previous version:

- The responses to the capillary order and aSMA staining are appropriate and accepted.
- The brief explanation of string vessels and how distinct they are from IPTNTs is useful.
- New figures and arrangements help streamline the presentation.
- The authors' explanation of the estimated high prevalence of IPTNTs is quite useful for the reader.
- Mechanistic speculations are welcome and reasonably presented in the discussion.

Overall, this is an intriguing study and I believe the the evidence presented is generally supported by data.

Osama Harraz, PhD

(Remarks on code availability)

Reviewer #4

(Remarks to the Author)
The revision has solidified the manuscript quite significantly. However, a few points still require clarification and improvement.

1) Ketamine is an effective antagonist of L-type Ca^{2+} channels (Cav1.2). Cav1.2 channels are highly expressed in pericytes and vascular smooth muscle cells, and ketamine may therefore unavoidably influence neurovascular coupling. In this context, simply stating that ketamine does not affect RGC activity may inadvertently downplay its potential impact on vascular signaling and neurovascular responses. Please discuss this limitation more explicitly and clarify how ketamine may (or may not) influence the conclusions related to neurovascular coupling.

2) I appreciate the new Supplementary Movie S2, which aims to demonstrate the identification of ON and OFF RGCs. However, I have concerns regarding both the representative video and the methodology, and further clarification is necessary.

Firstly, several neurons are labeled as “ON/OFF.” Do these represent a specific ON–OFF subtype, or are they cells that could not be unambiguously classified? If some RGCs cannot be confidently assigned as ON or OFF, how were they handled in the analysis and quantification?

Secondly, even with a 1 s or 3 s light stimulus, the PMT signal typically shows a marked increase or oversaturation artifact. Interestingly, the provided video appears to avoid obvious stimulation artifacts, despite the fact that the visual stimulus must be delivered to the same region being recorded by two-photon microscope. This suggests that the authors may have implemented a careful and novel experimental strategy. It would be valuable to describe this experimental design in detail so that readers can learn how stimulation artifacts were minimized.

Thirdly, calcium transients are long-lasting compared with action potentials and can persist for ~1–2 seconds (or longer) for a burst of spikes. As a result, determining whether transient onset is aligned with light onset versus light offset requires sufficiently high frame rate, spatial resolution, and signal-to-noise ratio. Based on the current data quality, it remains unclear to me how ON versus OFF classification can be reliably determined from the two-photon Ca^{2+} imaging traces alone. Please provide additional methodological details and quantitative criteria used for classification.

Fourthly, because these experiments are performed in vivo, small focus shifts along the z-axis (even subtle axial drift) can alter fluorescence intensity and could be misinterpreted as Ca^{2+} activity changes. How did the authors control for, detect, and/or correct z-motion artifacts in these recordings?

Lastly, in this video the authors applied two rounds of visual stimulation: the first to identify RGC subtypes surrounding the slower capillary, and the second to identify RGC subtypes surrounding the faster capillary. However, the response of the same RGC (marked with a red star) appears inconsistent between the two stimuli: the first stimulus suggests an ON-like response, whereas the second appears OFF-like. How do the authors interpret this discrepancy? Please clarify.

(Remarks on code availability)
NA

Reviewer #5

(Remarks to the Author)
I co-reviewed this manuscript with one of the reviewers who provided the listed reports. This is part of the Nature Communications initiative to facilitate training in peer review and to provide appropriate recognition for Early Career Researchers who co-review manuscripts.

(Remarks on code availability)

Version 2:

Reviewer comments:

Reviewer #2

(Remarks to the Author)
The authors have addressed all my comments. Congratulations on an excellent study.

(Remarks on code availability)

Reviewer #4

(Remarks to the Author)

(Remarks on code availability)

Reviewer #5

(Remarks to the Author)
I co-reviewed this manuscript with one of the reviewers who provided the listed reports. This is part of the Nature Communications initiative to facilitate training in peer review and to provide appropriate recognition for Early Career Researchers who co-review manuscripts.

(Remarks on code availability)

It seems that the authors used home-built R code for statistical data analysis. The R code is very well written. However, they didn't include the code to extract numbers from raw two-photon images and videos. And it is unclear to me which Figure plots / statistics the code are working with.

Open Access This Peer Review File is licensed under a Creative Commons Attribution 4.0 International License, which permits use, sharing, adaptation, distribution and reproduction in any medium or format, as long as you give appropriate credit to the original author(s) and the source, provide a link to the Creative Commons license, and indicate if changes were made.
In cases where reviewers are anonymous, credit should be given to 'Anonymous Referee' and the source.
The images or other third party material in this Peer Review File are included in the article's Creative Commons license, unless indicated otherwise in a credit line to the material. If material is not included in the article's Creative Commons license and your intended use is not permitted by statutory regulation or exceeds the permitted use, you will need to obtain permission directly from the copyright holder.
To view a copy of this license, visit https://creativecommons.org/licenses/by/4.0/

REVIEWER 1

Remarks to the Author

This is an interesting paper that provides new insights into the control of neurovascular coupling and suggests an intriguing hypothesis about the origins and roles of divergent behaviors of neighboring capillaries. In a previous paper, these authors demonstrated the existence of "interpericyte tunneling nanotubes" that connect pericytes associated with capillaries and mediate neurovascular coupling between capillaries that different in flow rate (Alarcon-Martinez et al., Nature, 2020). They also showed that the coupled capillaries exhibit bidirectional changes in behavior following light flash. (…capillary pairs connected by IP-TNTs exhibit opposite responses concomitant with light stimulation: one capillary dilates to increase blood flow, while the other constricts to decrease blood availability.") In the present paper, they provide further detail on this phenomenon and relate the differences in flow rate to the firing characteristics of neighboring retinal ganglion cells.

1) I have no technical criticisms but feel that the presentation could be improved. In particular, the analysis of ON and OFF cells seems to be central, yet it is introduced only after the description of light effect on vascular homogenization. I found this very confusing because it seemed to put emphasis on the value of homogenization, which may not in fact be the main issue. The beginning of this section says "…we considered whether this flux pattern might support a particular neuronal organization." But the results and the later discussion of them raises the possibility that the flux patterns may be not a correlate but rather the direct result of the "neuronal organization." As noted above, this seems to me to be is the major novel finding.

The simplest way to put it (perhaps a bit easier to understand than the way it is currently stated) is that OFF cells, which are active in the dark, are supplied by faster capillaries than ON cells, which are inactive in the dark. The flash activates ON cells, leading to greater flux in their slow capillaries, and deactivates OFF cells, leading to decreases flux in their fast capillaries. This is fascinating and important and should be introduced earlier and emphasized more. For example you could explain that there are ON and OFF cells that are differentially affected by a flash, then give the flash results, then come back to ask whether the result could be explain by the differential responses.

Response: We sincerely thank the reviewer for the feedback and agree with the comments. Accordingly, we have rearranged the manuscript following the reviewer's advice. Now, we have 8 main figures and 14 supplementary ones. We introduced earlier in the text the analysis of ON and OFF (now, Figure 2) and before the light-evoked homogenization (now, Figure 3).

2) The ON/OFF issue also raises some new points that are worth discussing. (a) Could something similar underlie heterogeneity in other parts of the brain or is this special?

Response: We thank the reviewer for this interesting question. Currently, our laboratory is studying whether there are similar IPTNT-dependent mechanisms in other parts of the CNS underlying blood flow heterogeneity (e.g., brain, optic nerve…). We think it is possible, as the brain also presents IPTNTs and neurons that can respond oppositely to the same stimuli. We have added it to the manuscript (lines 422-428) as follows:

"Particularly, the visual cortex and superior colliculus present orientation-selective neurons, which are preferentially activated by stimuli moving through one orientation, and inactivated when stimuli move orthogonally to it. Similar to the retina, IPTNTs may connect vessels supplying groups of neurons with opposite orientation selectivity, which could be impaired in disease. In vivo studies with transgenic mice expressing fluorescent IPTNTs are needed to clarify whether there is a general IPTNT-related mechanism underlying blood flow patterns in the CNS."

3) Might heterogeneity-homogeneity be an epiphenomenon of activity patterns? This would differ from the interpretation that the purpose of homogenization is to provide" a better oxygen supply into the extracellular space."

Response: We appreciate the reviewer's comment. We think that the idea of "*homogenization to provide a better oxygen supply into the extracellular space",* assuming that heterogeneity would not provide a good oxygenation, needs to be confirmed by further experiments. Actually, our results suggest that both homogeneity and heterogeneity are necessary to obtain the appropriate oxygen supply for the neurons activated during light and in the dark, respectively. We have updated the introduction (lines 66 and 71) and discussion (lines 318-320).

4) Are there really ON and OFF areas in the retina and if so how large are they? The polarity index is a start but given the mosaic arrangement of ON and OFF RGC types it seems odd; we need more information on this point.

Response: We thank the reviewer for bringing this important point. To our knowledge, ON and OFF areas have not been previously reported in the retina. However, it has been shown that the ON- and OFF RGC mosaics are consistently separated along the retina (PMID: 33692544). Thus, it is plausible that IPTNTs connect ON and OFF RGC mosaics at certain points where the polarity index between mosaics becomes larger, probably due to local variations in the distance between cells within a mosaic (intercellular distance range within a mosaic ~25-200 µm, PMID: 15312647; PMID: 36530520). Accordingly, we found that IPTNTs were mainly located in areas with a large ON-OFF difference. We have updated the text (lines 163-170) as follows:

"To our knowledge, ON and OFF areas have not been previously reported in the retina. Nevertheless, it has been shown that the ON- and OFF RGC mosaics are consistently separated along the retina. Thus, it is plausible that IPTNTs connect ON and OFF RGC mosaics at certain points where the polarity index between mosaics becomes larger. Accordingly, we found that 81% (35 out of 43) of pairs of RGC areas with a large ON-OFF difference (i.e., ≥ the mean of the ON-OFF difference between non-IPTNT connected areas + SD) were bridged by IPTNTs, suggesting that large opposite neuronal areas are equipped with IPTNTs to regulate differential blood flow."

5) The measurement of ON and OFF areas by calcium imaging stands on its own but extension to specific RGC types is puzzling, since the few types tested (F and alpha) comprise only a small fraction of all RGCs. Does their association with ON/OFF areas imply a global principle that has been missed? I can understand that this isn't the authors' intended focus, but having introduced it, they are obliged to deal with it the best they can, hopefully with available data.

Response: We sincerely appreciate the reviewer's feedback. *Ex vivo* identification of functionally-opposite neurons is challenging due to the limited number of available antibodies for this purpose. To overcome this

problem and confirm whether the functionally opposite areas around IPTNTs imply a global principle, we have used RNAscope to study another functional RGC type: the ON-OFF direction-selective RGCs (ooDSGCs). RNAscope uses probes for the detection of mRNA expression and can be done in thick tissue (PMID: 29974252), allowing us to identify ooDSGCs responding to opposite directions in the retina (i.e., dorsal direction: $RBPMS^{+}$/$Col25a1^{+}$/Calb1-; ventral direction: $RBPMS^{+}$/$Col25a1^{+}$/$Calb1^{+}$) (PMID: 31784286). We found a smaller polarity-index difference between IPTNT-connected areas for ventral/dorsal ooDSGCs compared to ON/OFF RGCs. We have updated the text (lines 170-178) and added new panels (Supplementary Figure S3G-J).

Specific comments

1) The paper is written in a very compressed manner that makes it difficult for nonspecialists to follow. The format suggests that it was prepared for Nature, which requires this sort of short format. However, Nature Communications does not. I urge the authors to expand some of the explanations, move a few key panels from the supplement to the main text (for example S5A and S11), and perhaps turn the 4 current figures into 5 or 6. It would also help to show rather than just describe the location of the capillaries being imaged. Small sketches of a cross section and a flat mount could be added to Figure 2 for this purpose. This can all be done while keeping the total length under that of typical papers in this journal – especially if some material already covered in the 2020 paper is compressed.

Response: We sincerely thank the reviewer for the feedback. Please see comment #1. We have also added a panel showing the area visualized in our *in vivo* experiments (Figure 1A) as well as a sketch showing the location of our *in vivo* imaging on the superficial plexus (Figure 3A).

2) Labeling on and legend for 1D could be expanded since this is the first of many panels of this sort. 100? 1? Flux? Velocity?

Response: Thank you. We have updated panel 1D and expanded its legend.

3) The significance of the results described in lines 121-135 (on αSMA) is completely obscure to this non-specialist, as is the reason the sentence starting on line 135 ("Before light….") is part of the same paragraph. Some explanation and interpretation is needed.

Response: We appreciate the reviewer's comment and apologize. Previous studies have suggested that vascular responses depend on the pericyte position within the vascular tree and the expression of αSMA. To clarify the significance of the results, we have relocated the figure (now, Figure 4) and updated the text (lines 205-220) as follows:

"*Due to the reported differences in vascular responses depending on the pericyte position within the vascular tree and the expression of α-smooth muscle actin (αSMA), we analyzed the location of pericytes expressing tunneling nanotubes as well as their αSMA expression. Importantly, rapid fixation methods are essential for proper αSMA detection post-mortem in the retina due to the quick actin depolymerization after death. Hence, differences between fixation protocols may introduce a large variability in αSMA expression between studies in the retina. In our study, we performed quick fixation via transcardial perfusion (see methods). The retinal superficial plexus was formed by 0th-order radial retinal arterioles branching from the central retina artery (CRA) and 1st-8th-order microvessels (Fig. 4A-E). IPTNTs were primarily located beyond the arteriole-capillary transition zone (TZ), identified as the zone where αSMA expression changes from high*

to low levels (Fig. 4F-H; see 'methods'). Thus, 20% of IPTNTs were located before/within the TZ (Fig. 4F-G, I) and 80% of IPTNTs were located beyond the TZ (Fig. 4H-I). We found that 57% of these IPTNTs beyond the TZ connect pericytes with at least one of them expressing some level of αSMA (Fig. 4J). This is in accordance with previous studies, where 70% of the IPTNTs connect at least one αSMA-positive pericyte in the superficial plexus."

4) Some explanation of the small arrow in 2C is needed, since it compares different slow and fast time points.

Response: Thank you so much for the comment. We have updated Figure 2C's legend (now, Figure 3C) to describe double-headed arrows as follows:

"[…] *small double-headed arrow shows the intercapillary difference after light calculated from the point of maximum increase in the slower capillary to the point of largest decrease in the faster capillary*"

REVIEWER 2

Remarks to the Author

This manuscript by Gardner-Russell et al provides an in-depth view of interpericyte tunneling nanotubule (IPTNT) function in the living retina using in vivo imaging. Using a combination of impressive techniques, including calcium imaging, optical ablations, and ischemia-reperfusion modeling, they conclude that IPTNTs are important for homogenization of flux between capillary segments needed for improved oxygen delivery to retinal tissues. The breadth of work is extensive and the general conclusions are supported by the data. Below, I provide several comments on presentation of the data and how specific details, if presented more clearly, could improve readability and support/refine the conclusions further.

Response: We sincerely thank the reviewer for the positive feedback.

1) The Main figures were rather crowded and would benefit from splitting into separate Figures to better explain the experiments. There are more than 20 small panels in Figure 3 for example, and this makes it hard to appreciate the context and detail of each experiment.

Response: We appreciate the reviewer's comments and agree. We have rearranged and updated the manuscript and figures. Now, we have 8 main figures and 14 supplementary ones. Please, see reviewer #1's main comment 1 and specific comment 1.

2) Some aspects of the supplement would be beneficial to move to main figures because they help understand the model system. For example, Supplementary Fig. 2 provides a better understanding of where IPTNTs are located within the network and would be good to provide as a main figure. Supplementary Fig. 2A is helpful in this regard, but this should be accompanied by a similar histogram of branch orders showing where alpha-SMA reduction occurs (end of transition zone). Prior work from the senior author (Alarcon-Martinez, Elife 2018) has shown that alpha-SMA expression extends up to 6th or 7th branch order in retina with proper fixation, but vessel branch ordering method differs than in the current manuscript. This should be clarified in relation to prior work.

Response: We thank the reviewer for bringing this important point. We have moved Supplementary Figure 2 to the main figures (now, Figure 4), including a new histogram of branch orders showing where αSMA reduction occurs (transition zone, TZ; Figure 4I). Please note that this does not mean that there is no αSMA after the transition zone, but a reduction.

We have also clarified (lines 921-922) that, in this study, the first-order vessel was defined as the first branch division from a whole radiating arteriole to compare with previous work in the brain, where the first-order vessel was identified as the first branch division from a penetrating arteriole.

3) Capillary diameter information is essential to show clearly with representative examples in the Main figures. There are currently 2 panels in Supplementary Fig. 4. This is a core part of the hypothesis that there is some dynamic regulation of capillary diameter by single IPTNTs, with opposing effects in neighboring capillary branches. The changes are small (5-10%) and proof of the ability to resolve this change is needed. The alternate possibility is that flux homogenization is not dependent on fast pericyte contraction/relaxation, but a result of red blood cell conformation change or increased intraluminal pressure to force cells through the capillaries.

Response: We thank the reviewer for bringing these important comments. We have moved the diameter panels to the main figures (Figure 3H-I). Importantly, we have confirmed that our system has the ability to resolve the light-evoked diameter changes. For this, we have quantified the maximum/minimum responses and confirmed that the change in each vessel is larger than the spatial resolution of our system (1 pixel = 0.3 µm). We have updated methods accordingly (lines 775-778).

4) Figure 1. Please report total flux among the two branches regulated by an IPTNT. Stimulation should lead to functional hyperemia and overall increased total blood flow. However, the graphs seems to suggest that total flux is similar if the slow capillary increases but the fast capillary decreases.

Response: We thank the reviewer for this important comment. We assume that the reviewer's comment is about the former Figure 2 (instead of former Figure 1), now Figure 3.

Please note that Supplementary Figure 9A, D show vessels with functional hyperemia, and Supplementary Figure 9B, C, E, F show the total flux/velocity (i.e., sum of slower and faster vessels). We found no overall increase in the total flux/velocity after light stimulation, but a redistribution of the blood flow. On the other hand, the total flux of non-IPTNT connected capillaries shows an overall increase in total blood flow (new Supplementary Figure S5).

5) Clarify whether Figures 1A-G are only for IPTNT contacted capillaries. This data should ideally be accompanied by capillary pairs that are not IPTNT-associated, so there is a comparison.

Response: We appreciate the reviewer's comment. We have added new panels for light-evoked flux/velocity changes in non-IPTNT connected capillaries (new Supplementary Figure S5). We have updated the text (lines 196-198).

6) Not all readers may be well versed in retinal neuron subtype identification (markers for ON and OFF RGCs). This part of the manuscript could provide more explanation.

Response: We thank the reviewer for the comment. We have expanded the explanation of RGC identification, justifying the study of RGC subpopulations in *ex vivo* retinas (lines 149-153 and lines 173-176).

7) The polarity index metric is only briefly explained in the Results and it is hard to understand what the metric is showing. The Methods and supplement help somewhat, but more explanation in the Main figure and results would help readers follow the analysis.

Response: We appreciate the reviewer's comment. We have added more explanation regarding the polarity index in the results (lines 137-143) and the legend (line 1090) of the main figure (now, Figure 2).

8) The laser ablation is helpful to test the specific role of IPTNT connections. Does this manipulation lead to death of the pericyte? Is there a control for the effects of laser induced injury itself?

Response: We thank the reviewer for allowing us to address this comment. To interrogate whether laser ablation leads to pericyte damage, we have performed *in vivo* IPTNT-laser ablation, identified the same IPTNTs in *ex vivo* flat-mounted retinas and applied a TUNEL assay to detect apoptosis. We found no signs of apoptosis. We replicated it in 7 IPTNTs of 4 mice. We have updated the text (lines 258-259) and added new panels (Figure 6F-G).

9) The authors say that the number of ON and OFF RGCs and their function are not changed with ischemia-reperfusion. It is surprising that neurons survive this insult and also maintain their network organization allowing the role of IPTNTs to be tested. Surely, the ischemia-reperfusion is causing major alterations to neuronal health/viability, unless the retina is more resilient than the brain. Some information on the neuronal consequences of the ischemia-reperfusion model would be warranted.

Response: We sincerely thank the reviewer for this important comment. It is reported that 60-minute retinal ischemia leads to the death of ~50% of the RGCs at 7 days after reperfusion (PMID: 11784707; PMID: 24970264). Since our analysis was focused on RGCs around IPTNTs, we wonder if our results may be influenced by it. Thus, we quantified the number of RGCs in areas without IPTNTs. We found that non-IPTNT areas show a reduction in the number of RGCs after reperfusion (Supplementary Figure 10F). Interestingly, after the prevention of the IPTNT rupture via MMP9 inhibition, areas with IPTNTs show a larger number of RGCs compared to regions without IPTNTs, suggesting a localized neuroprotective effect (Supplementary Figure 12D-E). We have updated the text (lines 275-280 and lines 293-297) and figures (Supplementary Figure 10F and Supplementary Figure 12D-E).

10) The authors should be cautious in their interpretations with the MMP9 inhibits. This is a nice experiment, but MMP9 inhibition may have many beneficial effects unrelated to preservation of IPTNTs. It is not an IPTNT-selective experiment.

Response: We thank the reviewer for the comment and totally agree. We have updated the text (lines 411-413), suggesting that pharmacological MMP9 inhibition may have beneficial effects via other cells.

11) The authors wish to extend their conclusions to the brain, but there is limited data. The data provided suggests a much lower density of IPTNTs in the superior colliculus and this is an argument against their contributions to brain capillary flow regulation. Clearly more data is needed, but the authors should be conservative and extrapolate their results to the brain (particularly in the abstract).

Response: We thank the reviewer for the comment and agree. We have lowered the tone of the extension of our findings to the brain. Thus, we have updated the abstract and discussion.

Minor/moderate

12) The presentation of some scatter plots look blurred (example, Fig 1O, P), while others are not (Fig. 1K,L). The blurring effect makes it hard to see the data points.

Response: We thank the reviewer for the comment and apologize. We have updated Figures 2H-I, 4B, 7H, 8E, S3B-F, S7G, S10D-E, and S12B-C.

13) Although the authors mention that capillary flux return the baseline after stimulation, the data shown suggests that they can drift away from baseline (Fig. 1C, C'). Are these ideal representative examples?

Response: We appreciate the comment. These are averaged traces. We have added longer recordings to the main figures to clearly show the flux return after stimulation (Figure 3J).

REVIEWER 3

Remarks to the Author

The manuscript by Gardner-Russel, Haddara and colleagues presents an interesting concept arguing that retinal blood flow is critically regulated by nanotubes connecting pericytes (IPTNTs). The manuscript includes impressive imagining in vivo and ex vivo and introduces provocative concepts regarding blood flow modulation in the CNS. While the draft doesn't really tackle the concept in the brain, the authors highlight differences in abundance of IPTNTs and suggest that future studies would be warranted to assess the concepts presented here but in the brain. While impressive and provocative, I have several critical concerns and confusions that cast doubts on the conclusions that the authors made. In my opinion, addressing these concerns is critical and necessary. As it stands, the evidence provided here is insufficient to justify the conclusions.

1) Capillary order and pericyte type: Studies exploring pericyte physiology have evolved (and many times contradicted one another). One major problem has been whether studies called different cells the same name or called the same cells different names. The capillary order starting with 1st order for the ones branching of the retinal arteriole and increasing till it reaches the venule (as shown in supplemental figure) has been a widely accepted standard. Here, the authors partially, but not sufficiently, address this point in Supplemental figure 2 using the alpha-smooth muscle actin (a-SMA) staining. I like Fig S2 and believe that it is very important and should even be moved to the main figures. All figures in the manuscript, however, should specify the orders of the capillaries where data were collected from. The authors could also include larger FOV images and then a zoomed-in illustration of the IPTNTs of interest.

Response: We sincerely thank the reviewer for bringing these important comments. We have moved Supplementary Figure S2 to the main figures (now, Figure 4).

We have also quantified and added small panels showing the order of the capillaries where the IPTNTs were located for each condition (i.e., intact, ischemia, sham ischemia, ischemia + MMP9 inhibitor, ischemia-reperfusion, sham ischemia-reperfusion, ischemia-reperfusion + vehicle, ischemia-reperfusion + MMP9 inhibitor). We have updated the panels accordingly (Figures 3C, 7I, 7J, 8A, 8F, 8G).

We have added a larger FOV image (new Figure 6A, which also shows multiple IPTNTs – arrowheads) as well as the location of the *in vivo* recordings identified in *ex vivo* retinas and a sketch of the usual *in vivo* area of recording (Figure 1A).

2) Related to the previous point, according to the presented calculations, ~80% of IPTNTs are beyond the transition zone (TZ), and of these IPTNTs more than half are aSMA positive. It seems to me that this cannot be correct based on the examples provided within. This also contradicts the data shown in Suppl. Fig. 2A, B where the authors argue that most nanotubes are around ~3rd to 6th order most of which one would expect absence of aSMA expression (e.g., PMID: 33051294). This is a major point for the authors to consider.

Response: We thank the reviewer for these important comments, and we are very grateful for having the chance to address them clearly.

Rapid fixation methods are essential for proper αSMA detection post-mortem due to the quick actin depolymerization after death. Accordingly, in the retina, we have previously demonstrated that pericytes located in 3rd and 6th-order capillaries can express αSMA, and its detection is dependent on the prevention of actin depolymerization with quick fixation techniques (PMID: 29561727). This has been corroborated in different studies in mouse and human retina (e.g., PMID: 32245120; PMID: 31675074; PMID: 39540858). Hence, differences between fixation protocols may explain the discrepancy in αSMA expression reported between studies in the retina. Particularly, in the reference that the reviewer kindly provided (i.e., PMID: 33051294), the authors firstly killed the animals and isolated the retinas, and then fixed the retinas for 15 min. In our study, we performed quicker fixation via PFA transcardial perfusion, keeping the retinas in PFA for another hour.

Here, we report that 80% of IPTNTs are beyond the transition zone (TZ, identified as the zone where αSMA expression changes from high to low levels), but <u>with still αSMA expression</u> in some pericytes (including those in 3rd-6th-order capillaries). Accordingly, our data shows that 57% of IPTNTs beyond the TZ connect pericytes with at least one of them expressing some level of αSMA. This is in accordance with previous studies from other labs, where it was found that 70% of the IPTNTs connected at least one αSMA-positive pericyte in the superficial plexus (PMID: 39540858).

We have updated the text to clarify this important point (lines 208-212 and lines 217-220).

3) It should be explicitly explained whether and how IPTNTs are distinct from string capillaries.

Response: We appreciate the reviewer's comment. IPTNTs are distinct from string capillaries (also named empty basement membrane tubes, empty sleeves, acellular capillary strands, intercapillary bridges, intervascular connective tissue strands, regressed vessels…) (PMID: 20634580). In contrast to string capillaries (PMID: 20634580), IPTNTs are not empty and contain organelles (PMID: 32788726). Furthermore, IPTNTs do not form a continuum with capillaries or endothelial cells and, instead, link pericytes in a soma-to-process or soma-to-soma configuration. Additionally, the diameter of IPTNTs (~500 nm; PMID: 32788726) is several-fold thinner than string capillaries (PMID: 25884288; PMID: 34675436; PMID: 12417377). We have updated the text (lines 85-90).

4) Movie S2: The vessels shown seem too large to be actually capillaries.

Response: We thank the reviewer for the comment and apologize. We have added a new video with the appropriate scale.

5) I am quite confused about the estimated high prevalence of IPTNTs and the fact that almost all example images show only very few. The authors should consider revising the calculations or explain why such huge discrepancy exist.

Response: We sincerely thank the reviewer for the comment and apologize for any confusion. We have revised our calculations, confirming our results. Our images are focused on the superficial plexus of the retina, where the RGCs are located. Here, the IPTNTs are less abundant than the other plexuses (PMID: 32788726). Please note that, although it is difficult to detect them at low magnification, Figure 4D shows five IPTNTs (arrowheads and the inset), and the new Figure 6A also shows five IPTNTs (arrowheads). Furthermore, during *in vivo* experiments, we need to focus each IPTNT precisely to detect it. To clearly show the prevalence of IPTNTs in our *in vivo* imaging, we have added a new *in vivo* z-stack video showing several IPTNTs in living recordings (Supplementary Movie S3). We have updated the text (lines 188-191) and figures (Supplementary figure S7F) to clarify this point.

6) It remains intriguing how IPTNTs could homogenize the blood flow between connected capillaries. How does this happen? At least this is warranted discussion.

Response: We appreciate the reviewer's comment. We have updated the discussion accordingly (lines 344-363 and 374-385).

REVIEWER 4

Remarks to the Author

This manuscript focuses on describing the functionality of blood flow regulation by interpericyte tunnelling in ON and OFF ganglion cell regions of the rodent retina. Using *in vivo* two-photon laser scanning microscopy, the authors measured retinal blood flow and IPTNT in the retinas of transgenic mice. They demonstrated that IPTNT-connected capillary pairs exhibit greater flow polarity at rest and, during light-evoked functional hyperaemia, the faster capillaries reduce flow speed while the slower capillaries increase it, thereby transitioning from heterogeneous to homogeneous flow. The authors later linked this phenomenon with the functionally opposite ON and OFF neuronal cell regions. Finally, they demonstrated that ischemia-reperfusion damaged IPTNT, which could be repaired by MMP9. This manuscript provides interesting and novel insights into the regulation of capillary blood flow by IPTNT, which is involved in the fine-tuning of microregional blood flow for different subtypes of neurons. However, the evidence supporting the conclusions on the specific role of IPTNT in regulating retinal blood flow is not strong enough. Furthermore, the underlying mechanism has not been investigated, although different hypotheses have been discussed in the manuscript.

1) All *in vivo* experiments were performed under anaesthesia. Previous studies have demonstrated that ketamine and isoflurane can significantly impact neurovascular dynamics by altering vascular tone and neurovascular mechanisms. Additionally, isoflurane is a potent vasodilator. The potentially fine-tuned capillary blood flow by IPTNTs is unnecessarily disturbed by the anaesthesia used by the authors. Further experiments are needed to demonstrate the functionality of IPTNTs in an awake state.

Response: We thank the reviewer for bringing this important point. Unfortunately, due to eye movements and ethical concerns (the retinal setup is different to the one used for brain imaging, where a

holder can be implanted), we can not perform these experiments in awake mice. We sincerely apologise for it. Importantly, note that we do not use isoflurane in our *in vivo* experiments (see Methods, lines 708-709). As the reviewer mentioned, isoflurane is a potent vasodilator, which may prevent further vasodilation during neuronal stimulation (PMID: 6391530; PMID: 20350606). Furthermore, isoflurane inhibits the retinal ganglion cell (RGC) function (PMID: 28638951), which will impact the neurovascular response in the retina.

On the other hand, ketamine does not inhibit RGC function (e.g., PMID: 28638951; PMID: 20011633) and induces minimal cardio-respiratory depression (PMID: 20350606). These are the reasons why we use ketamine in our *in vivo* experiments.

We have updated the methods (lines 706-708) and text to discuss the potential effect of anesthetics on our results (lines 385-395).

2) The authors demonstrated that the density of IPTNTs in the retina is approximately 4.5 times greater than in the cortex. Specifically, in Figure 3F, the authors used the superior colliculus as a representative region of the cortex, an area that is rarely studied in relation to cortical blood flow regulation. A more appropriate comparison would be the visual, somatosensory or motor cortex.

Response: We thank the reviewer for the comment. We have added quantifications for V1 (Supplementary Figure S7F-G). We also kept the values of the superior colliculus, as in the mouse, 90% of RGC axons project to it (PMID: 27169509).

3) Does the eye coverslip interfere with retinal blood flow? There is external pressure pushing towards the mouse's sclera to create sufficient contact area and potentially deform it in order to facilitate two-photon imaging.

Response: We thank the reviewer for the comment. To confirm that the coverslip did not interfere with blood flow, we have measured the blood flow in the same vessels with minimal contact area, with no increase of intraocular pressure (IOP) (11 mmHg; IOP without coverslip = 11 mmHg) and with our standard contact area (IOP = 12 mmHg). We found that there were no changes in blood flow between minimal and standard contact area (Supplementary Figure S1D). We have updated the text (lines 110-111), methods (lines 737-741), and added a new panel (Supplementary Fig. S1D).

4) The definition of 'non-connected capillaries' as a control for IPTNT-connected capillaries is unclear. If the length of IPTNT varies extensively from less than 5 µm to more than 100 µm (see Supplementary Fig. 3G), how did the authors select the non-connected capillaries in an unbiased manner?

Response: We thank the reviewer for the comment. We used vessels located at a distance = ~average of IPTNT length (i.e., ~50 µm). We have updated the text to clarify this point (lines 756-757).

5) Furthermore, the location of each capillary segment within the vascular tree, local blood pressure and capillary diameter are important factors in determining flux in this capillary, which varies drastically across the entire vasculature. It is therefore interesting to note that non-connected capillaries exhibit more equalised blood flow (Fig. 1K–P) than IPTNT-connected ones. This hardly makes sense. Do the results suggest that retinal capillary blood flow is homogeneous across the entire capillary bed, except in segments connected and polarised by IPTNTs?

Response: We appreciate the reviewer's comment. Please note that our study provides new insights into the formation of opposing behaviors of neighboring capillaries. Our results suggest that IPTNT-connected capillaries present a larger difference in blood flow/velocity than nearby non-connected capillaries. To clarify this point, we have updated the text (lines 105-109).

6) On page 5, lines 111–112, the authors claim that 'this suggests that IPTNTs are required for the regulation of local capillary blood flow patterns'. This claim is too strong. In regions without IPTNTs, there may be no need for heterogeneous capillary blood flow. Before discussing neuronal function in the results section, the authors can only claim that IPTNTs create a more heterogeneous pattern of capillary blood flow than regions without IPTNTs; they cannot claim that IPTNTs are 'required for regulation'.

Response: We appreciate the comment and completely agree. We have updated the text accordingly (lines 116-117).

7) To provide direct evidence that IPTNT regulates local capillary blood flow, light stimulation was applied to the entire region. This also affected the upstream arteries and arterioles, making it difficult to determine the specific function of IPTNT. It would therefore be necessary to manipulate IPTNTs individually and observe paired capillary flow changes, for example using optogenetic stimulation with laser scanning.

Response: We thank the reviewer for this comment and agree that an experiment where we can dissociate the responses of IPTNTs and upstream vessels will help to confirm the actual function of the IPTNTs. Please note that to provide direct evidence that IPTNT regulates local capillary blood flow, we measured light-evoked blood flow changes in the same IPTNT-connected capillaries before and after the depletion of the IPTNT. However, we agree with the reviewer that laser ablation may have an impact on the function of the upstream feeding vessels. To interrogate it, we have added a new experiment, where we measured the function of the common feeding vessel of a pair of IPTNT-connected capillaries before and after the laser-ablation of their associated IPTNT (new Figure 6). Our results show that upstream feeding vessels work similarly before and after the laser ablation of the IPTNT-connecting downstream capillaries, but light-evoked blood flow patterns do not occur appropriately anymore. We have updated the text (lines 258-259) and added a new figure (Figure 6).

8) In contrast to the conclusions of the Nature Neuroscience article (2024), with the publication code: 39232066, this submission concludes that individual blood vessels are not an accurate indicator of neuronal activity in their immediate vicinity. The text fails to provide an explanation for the discrepancy between the results of the two manuscripts.

Response: We sincerely thank the reviewer for the reference and agree that this excellent study should be considered in the discussion of our results (apologies!). We have updated the text (lines 415-428) as follows:

"*In contrast to our findings in the retina, a recent study in the mouse barrel cortex concluded that hemodynamic changes of single vessels were not accurate indicators of surrounding neuronal activity. The discrepancy between results may be due to the fact that our study focused on vessels connected with IPTNTs and their associated neuronal areas. On the other hand, Martineau et al did not identify vessels connected by IPTNTs or functionally opposite neuronal areas, which are important indicators for the location of IPTNTs. Furthermore, it is also possible that not all regions of the brain present IPTNTs. Thus, specific zones of the CNS may be more convenient for the study of the IPTNTs. Particularly, the visual cortex and superior colliculus present orientation-selective neurons, which are preferentially activated by stimuli moving*

through one orientation, and inactivated when stimuli move orthogonally to it. Similar to the retina, IPTNTs may connect vessels supplying groups of neurons with opposite orientation selectivity, which could be impaired in disease. In vivo studies with transgenic mice expressing fluorescent IPTNTs are needed to clarify whether there is a general IPTNT-related mechanism underlying blood flow patterns in the CNS."

9) As stated in the previous Nature paper by the authors, IPTNT was reported at two other plexus layers, which were found to be denser than the superficial layer. Could you please clarify why only the superficial layer was investigated? Please could you confirm whether the functions of IPTNTs are located at the other layers of the retina, where ON and OFF bipolar cells are also present?

Response: We thank the reviewer for the comment. We studied the superficial plexus because it is the one feeding the RGCs (lines 188). Our animals express GCaMP6 in RGCs but not in bipolar cells. Thus, further studies should analyze whether IPTNT-connecting vessels in other retinal plexuses also feed functionally-opposite neuronal areas. We have updated the text to clarify this point (lines 326-328).

10) Paper 15, line 329, 'hypoxia can occur at apparently normal blood flow levels', it has been argued by many papers that baseline blood flow is sufficient for the elevated metabolic needs (PMID: 35995628).

Response: We thank the reviewer for the comment and apologize for the misunderstanding. With our sentence, "*If blood flow patterns do not change appropriately during neuronal activity, hypoxia can occur at apparently normal blood flow levels*", we tried to describe that abnormal blood flow patterns, and not global changes in blood flow, could lead to hypoxia due to an inappropriate blood distribution. We have updated the text to clarify this point as follows (lines 396-402):

"Previous studies have suggested that basal blood flow is sufficient to supply oxygen and nutrients during neuronal activation. It is in accordance with our findings, where we show that no overall changes occurred after light stimulation, but, instead, a redistribution of the blood flow. Thus, precise regulation of blood flow patterns ensures that the right amount of oxygen is delivered to each group of neurons, homogenizing during functional activation and correlating with the stimulus intensity. Accordingly, if blood flow patterns do not change appropriately during neuronal activity, hypoxia may occur."

11) In the ischemia-reperfusion experiments, the authors only compared IPTNT before and after ischemia-reperfusion, and regrowth of IPTNT, as well as the application of MMP9. However, the authors cannot exclude impairment of ordinary pericytes, which are much more abundant than IPTNT. In this case, it is not possible to determine the mechanism by which the recovery of the ordinary pericytes, endothelial cells and neurons could contribute to the regained flow polarity and intercapillary difference.

Response: We thank the reviewer and agree with the comment that pharmacological MMP9 inhibition may have beneficial effects unrelated to preservation of IPTNTs. We have updated the text accordingly (lines 411-413). Additionally, to provide some clarity to this question, we have quantified the number of RGCs in areas with and without IPTNTs. We found that non-IPTNT areas show a clear reduction in the number of RGCs after ischemia-reperfusion injury (Supplementary Figure 10F), and that the prevention of the IPTNT rupture via MMP9 inhibition shows a localized neuroprotective effect in areas with IPTNTs compared to areas without IPTNTs (Supplementary Figure 12D-E). We have updated the text (lines 275-277 and lines 293-297) and figures (Supplementary Figure 10F and Supplementary Figure 12D-E).

12) With regard to the statistical analysis, given the multiple areas, cells and mice involved, the authors should employ a linear mixed effect model to test for statistical significance. It should be noted that the current usage of paired and unpaired t-tests, as well as other relevant tests, assumes that each measurement is independent. However, this is not the case. Therefore, it is imperative that the statistical analysis is redone.

Response: We sincerely thank the reviewer for the comment and for helping us to improve the statistics of the paper. As requested, we have redone all statistical analyses, including the use of a linear mixed-effects model for comparisons where measures were dependent. Please note that a paired t-test (also known as "*dependent t-test*") and Wilcoxon matched-pairs signed-rank test are used for dependent measurements (e.g., https://doi.org/10.1007/978-3-662-69107-6_14; https://doi.org/10.1007/978-981-97-3385-9_8). Accordingly, we used:

- A linear mixed-effects model for dependent cohorts (e.g., pairs of capillaries, polarity index of two related areas): Figure 1I, 1J, 1O-R, 2D, 2E, 2H-I, 3D-F, 3I, 5C-E, 5I-K, 6C'-D', Supplementary Figure S3B-E, S3H-I, S4C-E, S5C, S5F, S6B-C, S7A, S8B-D, S8G-I, S12E.
- Paired t-test or Wilcoxon matched-pairs signed-rank test (conditional on normality) to compare the same samples measured under different time/conditions (e.g., before vs. after light): Figure 3G, 5F, 5L, 5O-R, 6E', Supplementary Figure S1D, S4F, S6D, S8E, S8J, S8M-P, S9A-B, S9D-E, S9G-H, S9J-K.
- Unpaired two-tailed Student's t-test or Mann-Whitney U test (conditional on normality) to compare two independent cohorts (e.g., data from different animals): Figure 4B, 7E-F, 7H, 7K, 8B-E, 8H, Supplementary Figure S3F, S3J, S10B-F, S11C, S12A-D, S13C.
- Analysis of Variance (ANOVA) for multiple comparisons, followed by Tukey's tests or Kruskal-Wallis tests (conditional on normality), to compare 3 or more independent groups: Figure 7G, Supplementary Figure S7F-G.

We have updated the methods (lines 1001-1007), figures (Fig. 1I-J, 1O-Q, 2E, 5C, Supplementary Fig. S6B, S7A, S8B-C), and the legends of the figures.

13) As demonstrated in Figures 2C, 3B and 4I, the authors observed a consistent and gradual decline in flux within the faster capillary up to the conclusion of the recording. In contrast, the slower capillary returned to its baseline much earlier. Have authors an explanation for this result?

Response: Thank you for the comment. Disparities in the time of response may result from differences in the mechanism of regulation of intracellular calcium of each connected pericyte. We have updated the text as follows (lines 374-385):

"Furthermore, we found that IPTNT-connected capillaries showed different response times. This disparity in time probably reflects differences in the mechanism regulating intracellular calcium changes in each pericyte, such as the redistribution of intracellular calcium between connected pericytes via gap junctions, or the activation/deactivation of membrane receptors by products released from the ON- and OFF-areas. Thus, it is plausible that a different combination of mechanisms regulates the intracellular calcium in each connected pericyte, modifying the proportion of activated myosin light chain kinase (MLCK) and myosin light chain phosphatase (MLCP), and leading to disparate response times. Future studies should determine whether rapid vascular responses recorded at IPTNT-connected high-order capillaries in the retina of living mice are initiated in upstream arterioles/proximal pericytes with larger αSMA expression, and if they are also involved in the differences in the time of response found between 'slower' and 'faster' capillaries."

14) In Fig. 4B, did the authors revisit the same IPTNT after ischemia and further after reperfusion? Why do the diameter and distance of capillaries look very different from each other? Same question with IPTNT.

Response: Thank you. Please note that former Figure 4B (now, Figure 7B) shows *ex vivo* flat-mounted retinas labeled with antibodies (i.e., they are different retinas and IPTNTs).

15) Fig. 1K representative two-photon xy image, which shows basically clogged and no flow in the capillary at all, which conflicts with the linescan data on the right.

Response: Thanks for the comment. Please note that Figure 1K is the sum of multiple frames to facilitate the visualization of IPTNTs (it is not clogged). We have clarified it in the legend (line 1079).

16) Video 1 shows very well the paired capillaries linked with IPTNT, however, the important and novel discovery of this manuscript is that IPTNTs regulate blood flow during light stimulation from heterogeneity to homogeneity. I would envision the video should include the period after light stimulation and show linescan strips again.

Response: We thank the reviewer for the comment. We have added a new video showing the changes after light stimulation (new Supplementary Movie S1).

17) Video 2 provides a clear illustration of the responses of the neighbouring neurons. However, it lacks an indication of which is the ON cell and which is the OFF cell. As you will see from the video, the final figure is not fully explained in the manuscript. Could you please clarify what 'neuronal length' refers to for the neuron in the video that has been specifically marked?

Response: We appreciate the reviewer's comments. We have added a new video (new Supplementary Movie S2) identifying ON and OFF neurons, as well as the 'faster' and 'slower' capillaries. Neuronal length refers to the largest axis of the neuronal soma. We have excluded it to avoid confusion, as no axis analysis was performed in this study.

Reviewer #5 (Remarks to the Author):

I co-reviewed this manuscript with one of the reviewers who provided the listed reports. This is part of the Nature Communications initiative to facilitate training in peer review and to provide appropriate recognition for Early Career Researchers who co-review manuscripts.

Response: We sincerely appreciate the comments and help. Thank you!

REVIEWER 1

Remarks to the Author

My comments as Reviewer 1 were related more to style and presentation than to substance. The authors have done an excellent job of revising their paper in accordance with my suggestions. This is a terrific paper.

Response: We sincerely thank the reviewer for the positive feedback and all the help with the manuscript.

REVIEWER 2

Remarks to the Author

The authors have done a nice job addressing most of my comments. There are a few points related to two of my comments that were not fully addressed in my opinion:

1) Related to original comment #3 on capillary diameter information: The authors show graphs, but the readers will want to see high-resolution, representative images of the actual changes in the diameter of the capillaries. This would provide the proof of differential control of diameter between two vessels contacted by an IPTNT that underlies flux homogenization. The authors say that the resolution to detect it is sufficient, and images should be provided. There is a supplementary movie, but the resolution is too low to see this change. I recommend a supplementary figure that clearly demonstrates this diameter change happening, preferably with multiple examples.

Response: We sincerely thank the reviewer for the comment and apologize for the misunderstanding. To address this point clearly, we have added two examples: a new panel (Figure 3I) and a new video (Supplementary Movie S4) showing the opposite and simultaneous light-evoked diameter changes in two IPTNT-connected capillaries. We have updated the text (line 196) and the legends (lines 1147-1149; Supplemental Information, lines 236-238) accordingly.

2) Related to original comment #4 on total flux between capillary branches: I was expecting to see an increase of total flux with functional hyperemia, because retinal arterioles dilate in response to light stimulation. I think the authors need to clarify why this total blood flow increase is not seen, which is inconsistent with what we expect with functional hyperemia.

Response: We thank the reviewer for this important comment and appreciate the opportunity to address it. Previous studies with other retinal imaging techniques have shown global functional hyperemia after light stimuli (e.g., PMID: 20941368). To elucidate whether we can identify a global functional hyperemia in the retina after light stimulation, we have pooled all capillaries measured, including IPTNT-connected and non-connected ones. Our results show global

functional hyperemia after light stimulation. We have updated the text (lines 198-199), figures (Supplementary Figure S5), and legends (Supplemental Information, lines 67-77).

REVIEWER 3

Remarks to the Author

In the revised manuscript, the authors have done a good job addressing the comments I raised earlier. In particular, I find that the modifications of figures and text help clear confusions in the original previous version:

- The responses to the capillary order and aSMA staining are appropriate and accepted.
- The brief explanation of string vessels and how distinct they are from IPTNTs is useful.
- New figures and arrangements help streamline the presentation.
- The authors' explanation of the estimated high prevalence of IPTNTs is quite useful for the reader.
- Mechanistic speculations are welcome and reasonably presented in the discussion.

Overall, this is an intriguing study and I believe the evidence presented is generally supported by data.

Response: We sincerely appreciate all the feedback. Thank you!

REVIEWER 4

Remarks to the Author

The revision has solidified the manuscript quite significantly. However, a few points still require clarification and improvement.

Response: We sincerely thank the reviewer for the time and feedback.

1) Ketamine is an effective antagonist of L-type Ca^{2+} channels (Cav1.2). Cav1.2 channels are highly expressed in pericytes and vascular smooth muscle cells, and ketamine may therefore unavoidably influence neurovascular coupling. In this context, simply stating that ketamine does not affect RGC activity may inadvertently downplay its potential impact on vascular signaling and neurovascular responses. Please discuss this limitation more explicitly and clarify how ketamine may (or may not) influence the conclusions related to neurovascular coupling.

Response: We thank the reviewer for this important comment. To our knowledge, only high concentrations of ketamine (e.g., 500 µg/ml) directly infused during *in vitro* conditions on smooth muscle cells may totally inhibit L-type calcium channels (PMID: 32859919). Please note that the concentration of ketamine in tissue in our study is lower than this dose (PMID: 27144355).

Furthermore, previous studies with ketamine-anesthetized mice using a similar dose to ours (i.e., 90mg/Kg) show blockage of neurovascular coupling after nimodipine (L-type voltage-gated calcium channel blocker, Cav1.2), demonstrating functional L-type channels in ketamine-anesthetized mice (PMID: 40404520). We have updated the methods (lines 718-719) and the text (lines 396-399) as follows:

"*Additionally, it is important to mention that ketamine at high concentrations may block L-type calcium channels*[94]*. Although in our study we do not use these high concentrations*[95] *, and previous studies have shown functional L-type calcium channels in mice anesthetized with ketamine*[88]*, we may expect larger light-evoked responses in awake mice.*"

2) I appreciate the new Supplementary Movie S2, which aims to demonstrate the identification of ON and OFF RGCs. However, I have concerns regarding both the representative video and the methodology, and further clarification is necessary.

2.1) Firstly, several neurons are labeled as "ON/OFF." Do these represent a specific ON–OFF subtype, or are they cells that could not be unambiguously classified? If some RGCs cannot be confidently assigned as ON or OFF, how were they handled in the analysis and quantification?

Response: Thanks for the comment. Correct. Neurons labelled as "*ON/OFF*" represent a specific ON–OFF subtype. They contributed equally to the ON (positive) and OFF (negative) values for the polarity index of a region. We have updated the text, movie (we now use "*ON-OFF*" instead of "*ON/OFF*"), and methods (lines 906-907) as follows:

"ON-RGC related values were represented by positive values and grouped, and OFF-RGC related values by negative ones and grouped. ON-OFF-RGCs contributed equally to both groups."

2.2) Secondly, even with a 1 s or 3 s light stimulus, the PMT signal typically shows a marked increase or oversaturation artifact. Interestingly, the provided video appears to avoid obvious stimulation artifacts, despite the fact that the visual stimulus must be delivered to the same region being recorded by two-photon microscope. This suggests that the authors may have implemented a careful and novel experimental strategy. It would be valuable to describe this experimental design in detail so that readers can learn how stimulation artifacts were minimized.

Response: We appreciate the reviewer's comments. Correct. Our system is designed to automatically shut down the PMT when the light stimuli are presented. Then, an ImageJ/Fiji script subtracts any blank frames. We have updated the methods accordingly (lines 876-878) as follows:

"As photomultipliers (PMT) of the TPLSM saturate during light stimuli, our system was designed to automatically shut down the PMT when the light stimuli were presented, and an ImageJ/Fiji software (NIH) script subtracted any blank frames."

2.3) Thirdly, calcium transients are long-lasting compared with action potentials and can persist for ~1–2 seconds (or longer) for a burst of spikes. As a result, determining whether transient onset is aligned with light onset versus light offset requires sufficiently high frame rate, spatial resolution, and signal-to-noise ratio. Based on the current data quality, it remains unclear to me how ON versus OFF classification can be reliably determined from the two-photon Ca^{2+} imaging traces alone. Please provide additional methodological details and quantitative criteria used for classification.

Response: We thank the reviewer for the comment. To confirm whether RGC responses were aligned with the light offset, we presented a 3-second stimulus and identified responses only occurring after the stimulus. We have updated the method section accordingly (lines 878-883) as follows:

"*Thus, a one- or three-second light stimulus was presented to allow the characterization of both ON and OFF RGCs (transient and sustained), as well as ON/OFF subtype as previously[35]. Accordingly, we presented a 1-second stimulus to register the RGC response. Then, to determine whether RGC responses were aligned with the light onset or with the light offset, we presented a 3-second stimulus and identified if the response only occurred after the stimulus (i.e., aligned with the light offset).*"

2.4) Fourthly, because these experiments are performed in vivo, small focus shifts along the z-axis (even subtle axial drift) can alter fluorescence intensity and could be misinterpreted as Ca^{2+} activity changes. How did the authors control for, detect, and/or correct z-motion artifacts in these recordings?

Response: We appreciate the reviewer's comment. Animals were placed in a mouse holder to avoid movement. Recordings were maintained in focus manually throughout the whole acquisition period. Additionally, X-Y movements were corrected with the "*TurboReg*" plug-in of ImageJ/Fiji. We have updated the methods accordingly (lines 883-885) as follows:

"*Recordings were maintained in focus manually during the whole acquisition. X-Y movements were corrected with the "TurboReg" plug-in of ImageJ/Fiji software (NIH).*"

2.5) Lastly, in this video the authors applied two rounds of visual stimulation: the first to identify RGC subtypes surrounding the slower capillary, and the second to identify RGC subtypes surrounding the faster capillary. However, the response of the same RGC (marked with a red star) appears inconsistent between the two stimuli: the first stimulus suggests an ON-like response, whereas the second appears OFF-like. How do the authors interpret this discrepancy? Please clarify.

Response: We appreciate the reviewer's comments. We could not find the mentioned "*red star*". We re-analyzed the RGC responses and confirmed our results.

Importantly, when analyzing RGC responses, since each IPTNT-connected vessel is usually located at slightly different depths, we must focus on one capillary when presenting the light stimulus (which is why there are two stimuli in the video – one for each capillary's focus point). Unfocused RGCs may lead to misinterpretation of the results; thus, we analysed the response from RGCs when they were in focus. We have updated the legend of Supplementary Movie S2 to clarify this (Supplemental Information, lines 229-230).

Reviewer #5 (Remarks to the Author):

I co-reviewed this manuscript with one of the reviewers who provided the listed reports. This is part of the Nature Communications initiative to facilitate training in peer review and to provide appropriate recognition for Early Career Researchers who co-review manuscripts.

Response: We sincerely appreciate the comments and help. Thank you!

We hope we have satisfactorily addressed the reviewers' comments. Thank you very much for helping us to improve our study and for considering our revised manuscript.

We sincerely thank the reviewer, editor, and editorial team for their time. Below is our point-by-point response to the reviewer's comments. Direct verbatim quotations from the referees are shown in italics. We have also addressed the editorial requests, including the inclusion of the exact p-values and N values in all the figures. We have made minor changes to Figure 3D, 3H, 5I, and Supplementary Figure 7G. All changes are indicated by line number, and the text is red in the revised manuscript.

REVIEWER 2

Remarks to the Author

The authors have addressed all my comments. Congratulations on an excellent study.

Response: We sincerely thank the reviewer for the positive feedback and all the help with the manuscript.

Reviewer #5 (Remarks to the Author):

I co-reviewed this manuscript with one of the reviewers who provided the listed reports. This is part of the Nature Communications initiative to facilitate training in peer review and to provide appropriate recognition for Early Career Researchers who co-review manuscripts.

Response: We sincerely appreciate the time, comments, and help. Thank you!

Reviewer #5 (Remarks on code availability):

It seems that the authors used home-built R code for statistical data analysis. The R code is very well written. However, they didn't include the code to extract numbers from raw two-photon images and videos. And it is unclear to me which Figure plots / statistics the code are working with.

Response: Thanks for the comment. We did not use R code for statistical analysis. All statistics were performed with Prism 10 (GraphPad, Boston, MA), as described in lines 758-759.

For extracting raw data from TPLSM recordings, we have provided the code and two tutorial videos with file examples to use with our code (everything included in "Code.zip"). Furthermore, we have uploaded a CodeOcean capsule available with the article, where the readers can run the code online, obtaining the raw data from representative examples of our recordings. This code was used to obtain the raw data for panels: Figure 1G-J, 1M-T; Figure 3C-G, K; Figure 5B-F, 5H-R; Figure 6C', 6D',6E-E'; Figure 7I-L; Figure 8F-I; Supplementary Fig. 1C-D; Supplementary Fig. 4B-F; Supplementary Fig. 5A-H; Supplementary Fig. 6A-F; Supplementary Fig. 8A-P; Supplementary Fig. 9A-L; Supplementary Fig. 11A-D; Supplementary Fig. 13A-D. Calcium signal extraction was described in lines 613-615.

We hope we have satisfactorily addressed the reviewers' comments. Thank you very much for helping us to improve our study and for considering our revised manuscript.
